# Disease-economy trade-offs under alternative epidemic control strategies

**DOI:** 10.1038/s41467-022-30642-8

**Published:** 2022-06-09

**Authors:** Thomas Ash, Antonio M. Bento, Daniel Kaffine, Akhil Rao, Ana I. Bento

**Affiliations:** 1 Department of Economics, University of Southern California, Los Angeles, CA 90007 USA; 2 Sol Price School of Public Policy, University of Southern California, Los Angeles, CA 90007 USA; 3 National Bureau of Economic Research, Cambridge, MA 02138 USA; 4 Department of Economics, University of Colorado Boulder, Boulder, CO USA; 5 Department of Economics, Middlebury College, Middlebury, VT USA; 6 Department of Epidemiology & Biostatistics, School of Public Health, Indiana University, Bloomington, IN USA; 7 Pandemic Prevention Institute, Rockefeller Foundation, New York, NY USA

**Keywords:** Environmental economics, Population dynamics, Epidemiology, SARS-CoV-2

## Abstract

Public policy and academic debates regarding pandemic control strategies note disease-economy trade-offs, often prioritizing one outcome over the other. Using a calibrated, coupled epi-economic model of individual behavior embedded within the broader economy during a novel epidemic, we show that targeted isolation strategies can avert up to 91% of economic losses relative to voluntary isolation strategies. Unlike widely-used blanket lockdowns, economic savings of targeted isolation do not impose additional disease burdens, avoiding disease-economy trade-offs. Targeted isolation achieves this by addressing the fundamental coordination failure between infectious and susceptible individuals that drives the recession. Importantly, we show testing and compliance frictions can erode some of the gains from targeted isolation, but improving test quality unlocks the majority of the benefits of targeted isolation.

## Abstract (web summary)

Pandemic control policy requires balancing economic and disease outcomes. This study develops a joint modeling approach that allows both aspects to be considered simultaneously and shows that targeted isolation is superior economically and can achieve similar disease outcomes to voluntary isolation or blanket lockdowns.

## Introduction

To date, over 448 million individuals have been infected with SARS-CoV-2 and more than 6 million have died worldwide, with around 15% of these deaths happening in the United States, and only around 50% of the world’s population has received at least one vaccination^1^. The pandemic also triggered the sharpest economic recession in modern American history. According to the US Department of Commerce, during the second quarter of 2020 US Gross Domestic Product shrank at an annual rate of 32.9%^2^. The COVID-19 pandemic’s global repercussions exposed a need for coupled-systems frameworks that link epidemiological and economic models and assess potential disease-economy trade-offs. Such frameworks allow individuals’ adaptive responses to infection risks to be captured and can reveal important features of control strategies, such as the role of targeted isolation strategies that can overcome the fundamental coordination failure between infectious and susceptible individuals that drives the economic recession.

Broadly, four areas of study have informed the assessment of control strategies. Epidemiological studies evaluate disease dynamics and consider the heterogeneity of impacts resulting from control strategies^3–12^. Epi-economics studies consider the microfoundations of human behavior as drivers of the disease, as well as the costs and benefits of alternative control strategies^13–23^. An emerging literature on the macroeconomic consequences of pandemics considers the impacts of COVID-19 and various control strategies, either by embedding these behaviors in a broader economy with disease dynamics^24,25^ or by conducting detailed macroeconomic projections in the absence of disease dynamics^26,27^. In addition, numerous statistical analyses have examined the relationship between disease-related behaviors and economic activity^28–30^. Several knowledge gaps remain. For example, structurally mapping economic activities to contacts in a tractable fashion that retains the underlying heterogeneity of the population presents various challenges. One major challenge is how to calibrate this mapping using epidemiological social contact surveys, which contain data on potentially disease-transmitting contacts between individuals. Further, detailed individual economic behavior and epidemiological transmission mechanisms have typically not been embedded into models that consider the broader economy. Finally, the set of control strategies considered in coupled-systems models remains limited and overly simplified. To date, these models have not included individual-focused targeted isolation strategies, and the conditions under which these may overcome disease-economy trade-offs are unknown.

**Fig. 1:**
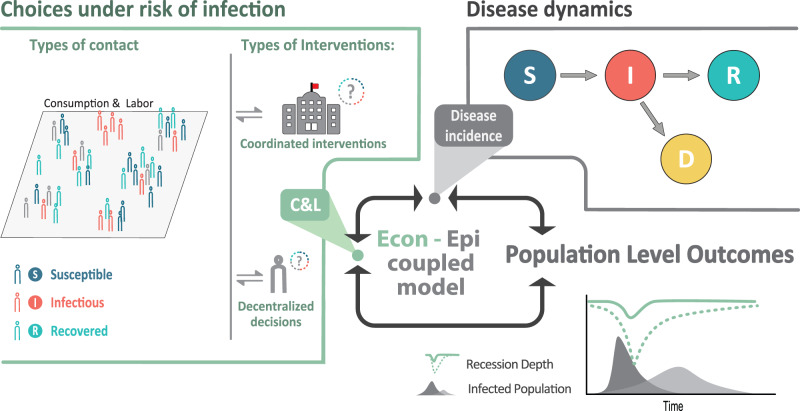
Coupled system schematic. Individuals make consumption (*C*) and labor-leisure (*L*) choices, considering the risk of infection through contacts with others. Individual choices and resulting contacts affect and are affected by the disease dynamics. Individual economic choices drive population-level outcomes such as disease prevalence and economic recessions. Under decentralized approaches, individuals optimize their behaviors based on their own preferences and health status. Under coordinated approaches, individuals' behaviors are optimized based on how they affect population-level outcomes.

To address these gaps, we develop a tractable coupled epi-economic model of individual microeconomic behavior embedded within the broader economy. Figure 1 presents a schematic representation of our model. Dynamic, forward-looking consumption and labor-leisure choices that account for the risk of infection are made by either (a) decentralized individuals or (b) coordinated policy interventions, in order to maximize perceived well-being (utility). These choices generate contacts that evolve endogenously in the model—i.e., contact rates affect and are affected by the disease dynamics. Depending on the activity, contacts can be avoidable or unavoidable. For example, in the U.S. economy, the average individual has around 7.5 contacts at their place of work during an 8-hour workday. These are avoidable contacts if the individual can alter their labor supply. In contrast, contacts such as those that occur at home are unavoidable and carry a risk of infection^31^. For analytical tractability, the framework initially assumes individuals have full information about their health status, and the presence of pre-symptomatic and asymptomatic individuals is reflected in the calibration of productivity losses as only individuals with no or mild symptoms will be able to work.

We model choices as reflecting individual preferences over time spent working/not working, how much to consume, and how to balance infection risk against the need to work for money and consume for well-being. The model solves this dynamic optimization problem of balancing risk and activity at the individual level and aggregates the solution across the population in order to determine economic recession and disease outcomes. This allows for direct calculation of disease-economy trade-offs that are grounded in individual behavior. Solutions to these types of dynamic optimization problems describe forward-looking individual behavior^32^, which is critical to modeling expectations during a pandemic. This does impose steep computational costs as the dimension of the state space increases^33^, necessitating simplifying assumptions for elements not central to our analysis.

In a decentralized choice setting, infectious individuals put susceptible individuals at risk and bear no direct consequences for this imposition (in economics terminology, an infectious individual creates a *negative externality* on a susceptible individual). Thus, a key challenge for infection control and avoiding economic losses is the inability of susceptible individuals to coordinate with infectious individuals and encourage them to reduce their activities and contacts^34^. Absent such coordination, susceptible individuals bear the full burden of adjusting their consumption and labor choices to minimize personal risk—we refer to this decentralized control strategy as “voluntary isolation” (a behavior documented in prior epidemics such as H1N1^35^,^36^). While not a policy intervention, this is still a “control strategy” because susceptible individuals manage their own infection risk. In contrast, a true “no control” strategy would (unrealistically) entail individuals making no adjustments to their behavior in response to infection dynamics (see SI 2.5).

Recognizing this coordination failure, we consider a policy intervention whereby a governing body (a *social planner* in economics) optimally coordinates labor and consumption choices in order to maximize aggregate well-being (utility), while still accounting for individual preferences. The “social planner” is a commonly-used methodological construct in economics to identify optimal strategies and inform policy design. This optimal coordination of labor and consumption generates a control strategy that targets infectious individuals—"targeted isolation”. In a world where the coordination failure is resolved, e.g., by paying infectious individuals to isolate, susceptible individuals can still consume, work, and engage in contacts, minimizing individual economic losses and the resulting recession. Importantly, to illustrate the general benefits of such targeting strategies, we abstract from many aspects of individual heterogeneity, often captured in epidemiological studies (e.g.,^9,37–39^). This is reasonable, since the fundamental coordination failure is itself independent of heterogeneity (see SI 2.6).

In real-world terms, a targeted isolation policy encompasses interventions aimed at encouraging infectious individuals to isolate themselves, with susceptible individuals isolating only if necessary to suppress infection growth. Such a policy could contain features like incentive payments to encourage individuals to obtain tests following possible exposure or symptoms, incentive payments for individuals to isolate following positive tests (e.g., compensation for lost wages), randomized compliance checks and penalties for individuals caught breaking isolation, etc. Implementation of such targeted isolation policies may be imperfect; we examine compliance scenarios to assess the degree to which such issues may limit the performance of targeted isolation policies.

We calibrate our model to pre-pandemic economic and social mixing data, using 2017 contact survey data from^6^ and next-generation matrix methods^40^ to generate a contact function linking different economic activities to contacts and to calibrate the transmission rate (see SI 2.3). Alternative parameter choices consider the role of online shopping and work that may have altered the underlying relationships between activities and contacts (see SI 5.4). The results described below hold across a wide range of plausible economic and epidemiological parameters.

We study the disease-economy trade-offs that result from three alternative control strategies: voluntary isolation, targeted isolation, and a blanket lockdown (see “Methods”). Under voluntary isolation, decentralized individuals continue to optimize their personal behavior based on preferences and health status. Some may isolate, others will not. By contrast, a policy of targeted isolation of infectious individuals is able to address the coordination failure, effectively separating susceptible individuals from infectious ones. Finally, to contrast these results against a commonly imposed control strategy, we also consider a blanket lockdown, whereby all individuals are forced to isolate, independent of their disease status. In the USA, for example, by April 15, 2020, more than 95% of the population was under a stay-at-home order^29,41^; such social distancing policies have been noted for their large economic costs and social disruption. Our focus is on the behavioral channels each control strategy utilizes to deliver disease control and economic benefits. Our coupled model makes it possible to identify these optimal strategies, and our calibration of key economic and epidemiological variables makes it possible to examine and quantify differences between these control strategies.

Our first contribution is the development of the tractable coupled epi-economic model described above that highlights the mechanisms and benefits that targeted isolation strategies have the potential to deliver. As noted, this model must prioritize the key mechanisms (e.g., the link between contacts and economy; the coordination problem) and impose valid assumptions to remain tractable in the face of steep computational costs. Of course in practice, implementing such targeted isolation strategies comes with challenges, particularly as our key assumption of full information regarding disease status may not hold in the early stages of an emerging disease epidemic. Thus, our second contribution is to apply the above model to consider a number of important frictions in a tractable way—for example, we allow for initial tests to be slow and of low specificity and sensitivity, which then improve over the course of the epidemic. This application demonstrates how our modeling approach can be applied to address some of the challenges associated with integrated disease models identified in ref. ^42^.

We show that the widely-used control strategies of voluntary isolation or blanket lockdowns suppress the epidemic nearly as effectively as targeted isolation, but are economically costly and impose a much deeper recession. Targeted isolation strategies avoid these sharp disease-economy trade-offs by incentivizing infectious individuals to isolate. This allows susceptible individuals to continue to consume and work, carrying the economy through the epidemic with a milder recession. Using targeted isolation strategies instead of voluntary isolation strategies can avert substantial costs—up to the order of *$*3.5 trillion in averted recessionary losses. Importantly, we show that relevant frictions (testing information, compliance) can erode some of the gains from targeted isolation, but availability of high-quality tests unlocks the majority of the benefits of targeted isolation. An implication of these findings is that the relative merits of targeted isolation versus blanket lockdowns at any given point in time depend on the test environment and other features of the emerging disease system.

In the following sections, we begin by abstracting from information- and compliance-related frictions in order to illustrate the key model mechanisms and our methodological contributions. That is, we assume all agents perfectly know their health status and the current distribution of health statuses across the population, and fully comply with all policy mandates. Having established the underlying mechanisms, we then analyze model applications that introduce lags in test reporting, uncertainty due to limited test quality, and partial compliance with lockdown strategies and targeted isolation.

## Results

### Methodological contributions

**Fig. 2:**
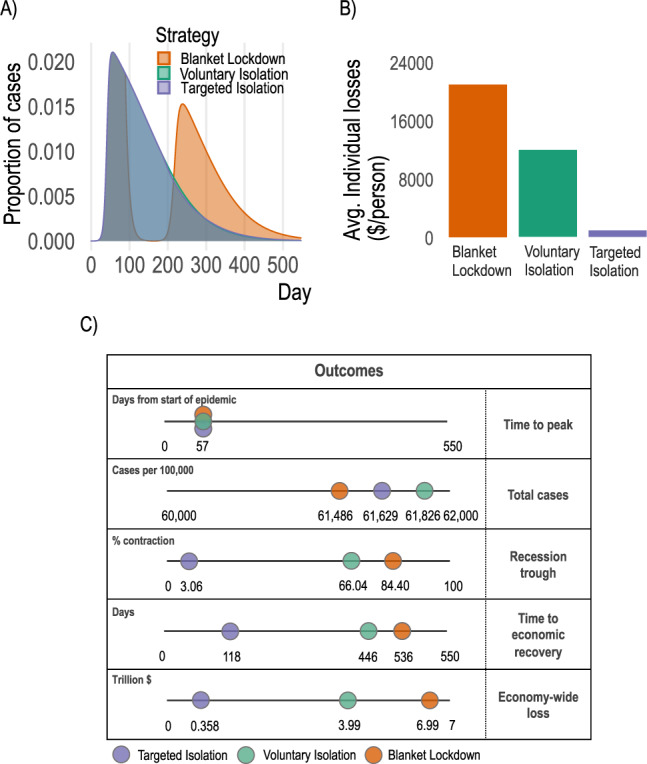
Disease dynamics and economic outcomes under voluntary isolation, blanket lockdown, and targeted isolation. **A** Proportion of population infected over time under each strategy. Voluntary isolation and targeted isolation curves are almost-entirely overlapping, indicating nearly-identical disease dynamics. **B** Individual losses incurred under each strategy (targeted isolation averts 95% of voluntary isolation individual economic losses). **C** Key aggregate disease and economy outcomes under each strategy. See SI 2.5 for comparison with a “no control” approach.

Our first contribution is methodological: we construct a data-driven, theoretically-consistent coupled epi-economic model which can be used to study important properties of novel pathogens in economies. We emphasize two core model features. First, regardless of control strategy, in our model the SARS-CoV-2 epidemic spreads rapidly in the population, with peak daily incidence early in the epidemic (Fig. 2A, C), and final proportions of the population exposed (Fig. 2C) are largely unaltered. A plausible blanket lockdown designed to minimize total cases (see SI 4.1) can indeed reduce cases relative to targeted or voluntary isolation strategies, however it leads to a rebound (Fig. 2A) when the lockdown is relaxed. This rebound is observed in all blanket lockdown scenarios considered, including when the lockdown is combined with additional non-pharmaceutical interventions such as shifting to more online activity—SI Figs. S7, S11 and S13. In the discussion we describe how blanket lockdowns may still have useful complementarities with targeted isolation despite the potential for rebounds. All “control” strategies nevertheless significantly outperform a “no control” strategy where neither individuals nor a social planner optimize behavior (shown in SI Fig. S3). Second, under a targeted isolation strategy, disease control does not come at as large an economic cost as under voluntary isolation or a case-minimizing blanket lockdown (Fig. 2B, C). In aggregate terms, targeted isolation converts an historically severe recession (66% peak-to-trough contraction under voluntary isolation, 84% under the blanket lockdown) to a mild and not-atypical one (3% peak-to-trough contraction). By coordinating individuals’ behavior over the course of the epidemic, targeted isolation can minimize the disease-economy trade-off imposed by voluntary isolation and blanket lockdown strategies.

**Fig. 3:**
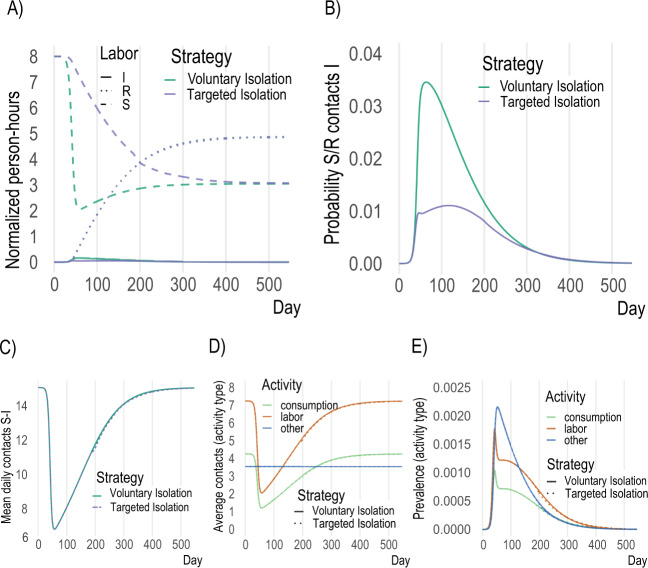
Key model mechanisms. **A** In voluntary isolation, susceptible individuals withdraw from economic activity due to the presence of infectious individuals (green dashed), while under targeted isolation susceptible agents engage in much more economic activity (blue dashed). **B** More infectious agents at activity sites under voluntary isolation lead to higher probability of infection throughout epidemic. **C**–**E** overall contacts, contacts by activity and prevalence (% infectious) do not change meaningfully across voluntary and targeted isolation, as the same infection outcomes are achieved despite enabling far more activity by susceptible individuals with targeted isolation.

The large economic savings (91% of individual economic losses averted) and the marked difference in the probability of contact (Fig. 3B) from the targeted isolation strategy arise primarily from shifting the burden of isolating from susceptible to infectious individuals (Fig. 3A). Under a voluntary isolation strategy, some infectious individuals continue to work and consume despite the risk they impose on others^43–45^. This is the key coordination failure that increases the probability of infection (Fig. 3B) and forces susceptible individuals to work and consume less to avoid infection (Fig. 3A). Since susceptible individuals are the majority of the population in a novel epidemic, this approach to disease control comes at a large economic cost. By contrast, targeting isolation at infectious individuals dramatically changes the composition of the pool of people working and consuming (Fig. 3A & B). Voluntary isolation at the epidemic peak leads to about 3 fewer hours spent at consumption activities and 6 fewer hours spent at labor activities per day by *susceptible* individuals, while targeted isolation reduces *infectious* individuals’ activities by similar amounts (Table S2). This does not cause changes in mean daily contacts between strategies (Fig. 3C, D), nor prevalence by activity type (Fig. 3E), even though many more susceptible individuals are able to work and consume. As a consequence, targeting delivers small improvements in infection outcomes but massive economic savings.

Further infection reductions are *possible* under these control strategies, however reducing cases even by a small amount quickly increases economic costs (SI Fig. S5; to achieve the minimum level of cases, economic losses are multiplied nearly tenfold). This result, that further disease reductions are only possible with extreme economic losses, is an intuitive consequence of an optimized solution.

### Model applications

Our second contribution is to apply the model to COVID-19 in the USA and study how key frictions with plausible magnitudes may affect the model mechanisms and resulting policy conclusions. We focus on two types of frictions which are particularly relevant to novel epidemics: limited or delayed health status information, and individual non-compliance with policy directives. These frictions are modeled as particular scenarios (see “Methods”). Importantly, we deliver a tractable and plausible analysis of these frictions in a coupled epi-economic model, though we acknowledge that there is much research to be done on the microeconomic foundations of individual behavior in the face of a novel pathogen.

**Fig. 4:**
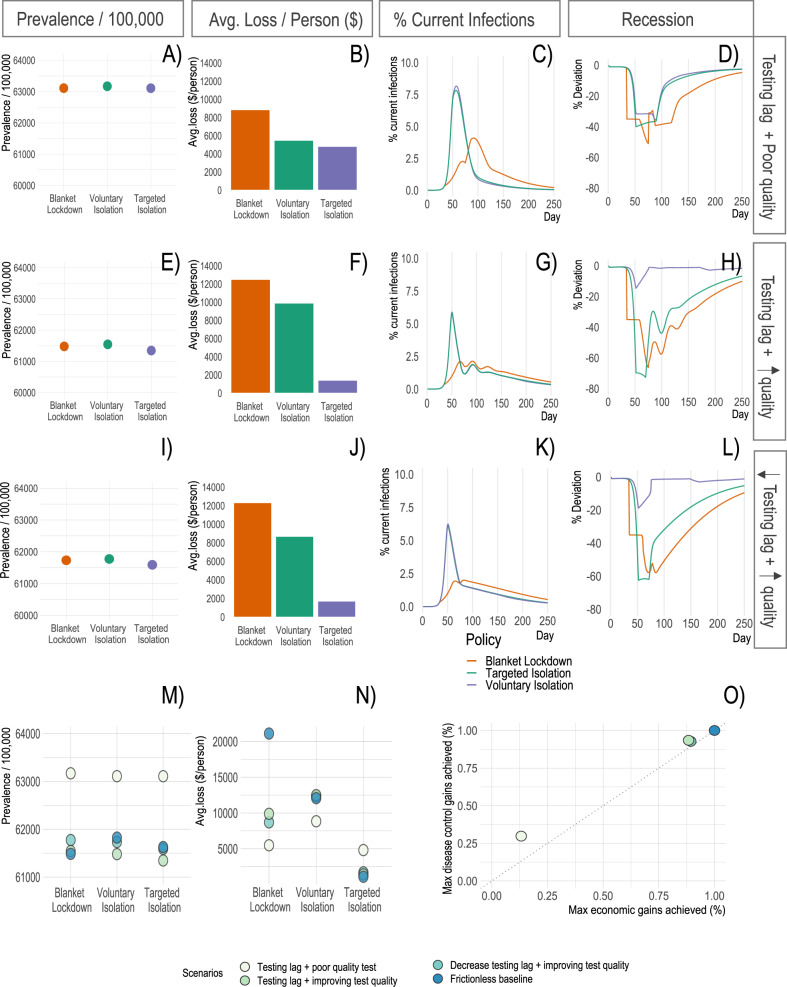
Model outcomes with different information frictions. **A**–**D** Key model outcomes under 10% test quality and an 8-day lag between testing and reporting. **E**–**H** These outcomes when test quality linearly improves from 10% to 95% quality by day 75 under a constant 8-day test reporting lag. **I**-**L** These outcomes when test quality linearly improves as before, and the test reporting lag reduces from 8 days to 5 days at day 60 then from 5 days to 3 days at day 75. **M**, **N** How disease-economy outcomes vary across these scenarios. **O** The disease-economy outcomes under these scenarios relative to the baseline in Fig. 2.

Our first scenarios vary test quality and delays in detecting infectiousness (Fig. 4). The above results in Fig. 2 assume full knowledge of infection status (i.e., regular and accurate testing) for both voluntary and targeted isolation. Here, we consider scenarios where (a) individuals take a test that correctly reveals their infection status with X% probability (X determined by the scenario), and (b) the test result is received only Y days (Y determined by the scenario) after the individual actually becomes infectious. The latter could be either because the test is taken some time after infection, or there is a lag between taking the test and receiving the results. These two dimensions cover a broad range of population-level testing strategies, though a detailed modeling of all possible testing strategies is beyond the scope of this paper (see SI Fig. S7 for cases where individuals are uncertain over their own infection status and respond to each others’ uncertainty).

In the “limited and delayed testing” scenario (10% test quality, 8-day test lag), targeted isolation can only recover around 13% of the economic benefits from targeted isolation in the baseline scenario, and only around 30% of the infection control benefits from targeting. This scenario demonstrates that the benefits of targeted isolation are conditional on the quality of the testing regime. In the “improving test quality” scenario (95% test quality after 75 days, 8 day test lag), targeted isolation can recover nearly 92% of the economic benefits from targeting in the baseline scenario, and nearly 94% of the infection control benefits; note this scenario is intended to be consistent with the observed evolution of testing capability during the COVID-19 pandemic. These results are largely unchanged in the “improving test quality and delays” scenario (95% test quality after 75 days, 5 day test lag after 60 days, and 3 days after day 75). The improvement in test timeliness has very little effect over and above the effect of improved test quality, and the changes in transient dynamics can even reduce some of the economic and infection control benefits. In all cases, the voluntary or targeted isolation policies deliver substantial economic benefits over blanket lockdowns, while blanket lockdowns deliver greater infection control benefits. We explore the robustness of these results to alternative assumptions on equilibrium behavior under low-quality information, finding it does not change the ranking of policies in terms of economic benefits but may alter the ranking over infection control benefits (see SI 3.3.4).

These results highlight two important channels through which targeted isolation delivers improvements over voluntary isolation. First, as expected, better information tends to enable better implementation of targeted isolation. Second, however, better information can also worsen the recession under voluntary isolation. Intuitively, poor information mitigates the coordination failure by leading individuals uncertain about their health type to act as though they are a different type. Many infectious individuals who would otherwise impose externalities on others end up acting as though they are susceptible and reducing their labor supply and consumption. Similarly, many susceptible individuals act as though they are asymptomatic or recovered and continue to supply labor and consume, mitigating the recession severity.

**Fig. 5:**
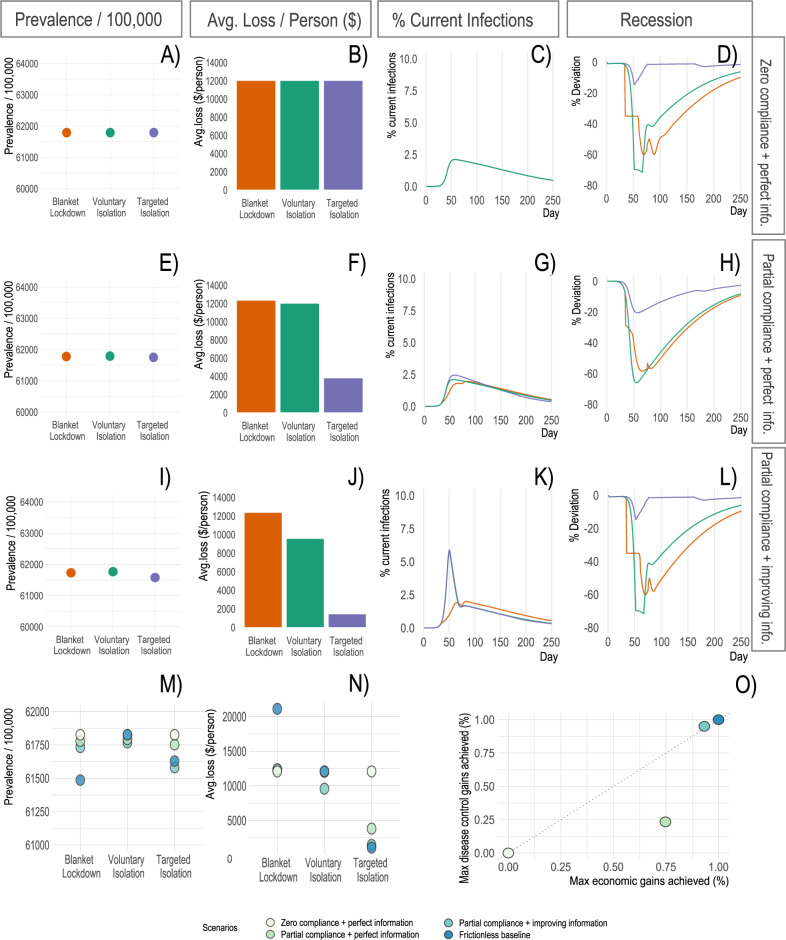
Model outcomes with different compliance rates. **A**–**D** Key model outcomes under 0% compliance and no information frictions. **E**–**H** These outcomes under 75% compliance and no information frictions. **I**–**L** These outcomes when test quality linearly improves from 10% to 95% by day 75 and the test reporting lag reduces from 8 days to 5 days at day 60 then from 5 days to 3 days at day 75. **M**, **N** How disease-economy outcomes vary across these scenarios. **O** The disease-economy outcomes under these scenarios relative to the baseline in Fig. 2.

Our next scenarios vary the fraction of individuals who comply with policy mandates such as blanket lockdowns or targeted isolation (Fig. 5). In the “low compliance and perfect information” scenario (0% compliance rate, no information frictions), targeted isolation is ineffective. In the “partial compliance and perfect information” scenario (75% compliance, no information frictions), targeted isolation recovers just over 76% of the economic benefits from targeting in the baseline scenario, and nearly all of the infection control benefits. These results are intuitive given the properties of targeted and voluntary isolation in the baseline model: since the non-compliant share of the population behaves as they would under voluntary isolation, the benefits realized are a convex combination of those from voluntary and targeted isolation. This result also helps to clarify the role of altruism, for example^43,46^ show that altruistic motives can induce some infectious individuals to isolate without control strategies. However, if there is still a large enough portion not acting altruistically (as documented by ref. ^46^) sizeable targeted isolation benefits remain.

Finally, in the “partial compliance and improving information scenario” (75% compliance, 95% test quality after 75 days, 5 day test lag after 60 days), targeted isolation recovers roughly 95% of the economic benefits from targeting in the baseline scenario, and roughly 95% of the infection control benefits as well. Although compliance is unchanged, the percentage of benefits obtained improve for the same reason as in the purely information scenarios—worse information at certain levels can improve outcomes as infectious individuals act as susceptibles, solving the coordination failure. Taken together, our results provide qualitative insights into the importance of information quality, information timeliness, and compliance on the benefits of targeted isolation policies.

### Robustness to assumptions on coupling parameters and functions

To assess the robustness of our conclusions to these modeling choices, we conduct sensitivity analysis over several relevant model parameters. The main functional form for the contact function is assumed to be linear, such that additional labor and consumption activities increase (infection-risking) contacts proportionally. However, the types of social networks that individuals belong to and the nature of their interactions affects the mapping between activities and contacts^47^, and therefore we test other functional forms that aggregate how different network structures could affect this mapping. Next, we test the sensitivity of the calibration of the contact function, which is based on pre-pandemic contact data. This calibration aggregates detailed data on individuals’ social network structures up to model features like overall contacts at different activities. Finally, because we incorporate the impact of asymptomatic individuals through productivity losses (see “Methods”), we test the sensitivity of our model to the share of asymptomatic individuals by varying the productivity losses from infection. In the following sensitivity analyses, we focus on the core model, devoid of information- or compliance-related frictions, to identify how these modeling choices affect the maximum possible gains from targeted isolation.

**Fig. 6:**
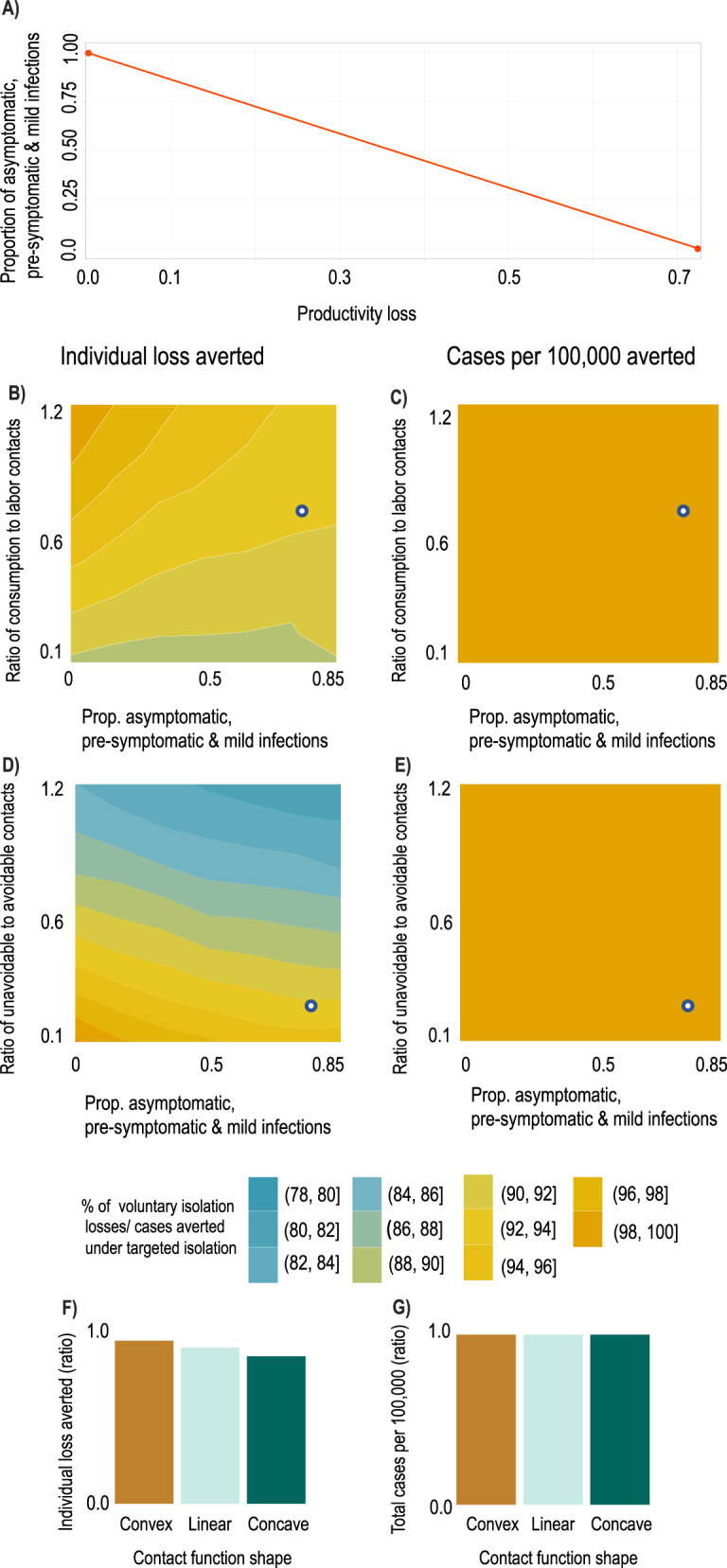
Result sensitivity to key model parameters. We plot ratios of outcomes under targeted vs. voluntary isolation to highlight the relative variation in outcomes under each strategy. The white dots in panels B–E show the baseline parameterization. **A** Mapping between productivity losses and implied share of the population which is pre-symptomatic, asymptomatic, or has mild symptoms (i.e., infectious individuals able to work). A productivity loss of 0.85 implies approximately 80% of the population are pre-symptomatic, asymptomatic, or have mild symptoms. Ratio of individual losses averted (**B**) and ratio of cases per 100k averted (**C**) under targeted isolation vs. voluntary isolation as proportion of contacts at consumption relative to labor activities increases (a value of 1 means equal number of contacts at consumption and labor) and as the asymptomatic share increases. Ratio of individual losses averted (**D**) and ratio of cases per 100k averted (**E**) under targeted isolation vs. voluntary isolation as proportion of unavoidable contacts (e.g., home) relative to avoidable contacts (consumption & labor) increases (a value of 1 means an equal number of contacts at home as at consumption & labor) and as the asymptomatic share increases. Ratio of individual losses averted (**F**) and ratio of cases per 100k averted (**G**) under targeted isolation vs. voluntary isolation as contact functional form varies. Convex contact functions imply high-contact activities are easiest to avoid, while concave contact functions imply low-contact activities are easiest to avoid (see “Methods”).

First, we show the mapping between prevalence of asymptomatic individuals and productivity losses from infection in Fig. 6A. Our productivity parameter is a weighted average of those experiencing no symptoms when infected (asymptomatic; able to work unimpeded without a loss to productivity), and those experiencing symptoms (less able to work during infection and thus incurring a productivity loss). A lower productivity loss in the figure implies more asymptomatic individuals. Figure 6B–E demonstrates the robustness of our conclusion that targeted isolation reduces economic losses without changing disease outcomes—varying asymptomatic infections through productivity (i.e., moving horizontally in the charts) does not significantly change the shading.

Second, because structural changes in the economy during the pandemic may have reduced the number of contacts per unit of activity (e.g., increased prevalence of contactless goods delivery, increased mask use or other non-pharmaceutical interventions), we examine our findings’ robustness by altering the ratios of contacts at different activities. Figure 6B-E show how our conclusions about targeted isolation relative to voluntary isolation change as we vary the contact structure of the economy and the proportion of non-severely-diseased individuals. From the white dots (main model calibration), moving to the left in Fig. 6B–E shows that lower prevalence of asymptomatic individuals will increase the economic effectiveness of targeted isolation without affecting the relative number of cases averted. Moving up the vertical axis in Fig. 6B, C shows that increasing the share of contacts that occur at consumption rather than labor activities (e.g., if remote work becomes more common while bars and restaurants remain open) would again increase the economic effectiveness of targeted isolation without affecting the relative number of cases averted. Moving up the vertical axis of Fig. 6D, E shows that increasing the share of contacts at unavoidable activities (e.g., if consumption and labor become increasingly contactless, or if more contacts occur during unavoidable activities such as religious or family gatherings) will reduce the economic effectiveness of targeted isolation without affecting the relative number of cases averted.

Lastly, the functional form of the contact function allows us to examine heterogeneity in contact rates—Fig. 6F, G. The mapping between activities and contacts will be an aggregation of individuals’ social network structures when consuming and working. Different functional forms approximate different mappings. Convex contact functions emerge when high-contact activities (individuals) are reduced (isolated) first and concave functions emerge when high-contact activities (individuals) are reduced (isolated) last. Targeted isolation accounting for these choices is likely to produce convex contact functions if high-contact activities (individuals) are reduced (isolated) first. We find such variations have a modest impact on the economic effectiveness of targeted isolation, but do not affect its disease control properties. We discuss these forms further in “Methods” and the SI and show the sensitivity of our findings to plausible variations in other structural parameters in SI Fig. S9.

## Discussion

Close to two years into the SARS-CoV-2 pandemic, it is increasingly clear economic concerns cannot be neglected^48^. We show that even in scenarios with imperfect testing and compliance, a targeted isolation approach emerges from our model as an optimal strategy, which balances disease spread and economic activities. Our predicted infection rates and economic responses are broadly consistent with observed patterns (see SI 2.7), and thus our results likely capture the correct order of magnitude and capture the key qualitative features of the epidemic and recession.

Recent studies suggest the COVID-19 recession was driven by voluntary reductions in consumption in response to increasing infection risk^28,49,50^. We show this drop in consumption is driven by a coordination failure: infectious individuals do not face the full social costs of their activities, leading susceptible individuals to withdraw from economic activity. This coordination failure resembles the classical problem of the tragedy of the commons in natural resources and the environment^51–53^, underscoring the lack of property rights in the market for infection-free common spaces. It also shares similarities with coordination issues that emerge in climate change, fisheries, orbit use and other settings^54–57^. Correcting this coordination failure via a targeted isolation strategy that internalizes the costs infectious individuals impose on susceptible individuals delivers substantial economic savings (Figs. 2 and 3).

Our conclusions arise from a data-driven method to calibrate the mapping between disease-transmitting contacts and economic activities. Compartmental models of infectious diseases typically segment activities based on population characteristics like age and student status (e.g.,^7,37,38,58,59^) rather than economic choices like consumption and labor. We build on prior work in this area (e.g.^15,16,18^) to address two long-standing challenges: appropriately converting units of disease-transmitting contacts into units of economic activities (contacts into dollars and hours), and calibrating the resulting contact function to produce the desired ${{{{{{{{\mathcal{R}}}}}}}}}_{0}$. We address these challenges in three steps (see “Methods” and SI).

First, we use contact matrices from ref. ^6^ to construct age-structured contact matrices at consumption, labor, and unavoidable other activities (Fig. S1). We then use next-generation matrix methods to calculate the mean number of contacts, adjusted for how individuals of different ages mix with each other, at each activity. Finally, we use these values with pre-epidemic consumption and labor supply levels to map contacts to dollars and hours in the contact function, and then calibrate the ${{{{{{{{\mathcal{R}}}}}}}}}_{0}$. This approach provides a behaviorally-grounded perspective on why contacts occur. Understanding the structure and benefits of targeted isolation requires this mapping between economic activities and contacts. We calibrate the model to pre-pandemic economic behavior. We validate the model’s performance along aggregate disease-economy dimensions in SI 2.8 and Fig. S5.

Our results also serve to highlight an important benefit of making high-quality testing for novel pathogens widely available early on^60–63^—it facilitates targeted isolation approaches which reduce economic losses. Targeted isolation provides the greatest benefits when information quality and compliance are high, with testing lags playing a relatively minor role. These results suggest that by enabling targeted isolation policies, early provision of high-quality testing combined with incentives to comply with policy directives can unlock some infection control benefits and substantial economic benefits. When testing is low-quality throughout the epidemic, the targeted isolation solution resembles a blanket lockdown (see Fig. 4).

While we have shown that information frictions and non-compliance can be important factors limiting policy effectiveness, we stress the fundamental problem is an inability to coordinate among susceptible individuals. In an ideal world, a market would exist allowing susceptible individuals, who do not want to be exposed to infection, to club together to pay infectious individuals to stay out of common spaces (e.g., gyms, restaurants, supermarkets). Even in the face of information and compliance issues, this would solve the fundamental problem and allow susceptible individuals to continue working and consuming, removing the disease-economy trade-off inherent in lockdown approaches. In reality, this is not possible, since susceptible individuals cannot coordinate (from economic theory, no “property rights” exist determining specifically whether susceptible or infectious individuals have a right to enter these spaces and should therefore be paid to access them, unlike most markets)^64^. Since this ideal or first best solution is not possible, targeted isolation is a policy solution to solve this coordination problem.

To implement targeted isolation, governments can provide incentives and encouragement for infectious individuals to remove themselves from these public spaces. In our model, paying individuals to stay home while infectious would require spending on the order of *$*428 billion (two weeks pay times the total number of infected), to purchase the gain of an avoided recession on the order of *$*4 trillion—total savings of up to *$*3.5 trillion relative to voluntary isolation (see Fig. 2), not including additional averted costs from long-term negative public health outcomes^27^. These findings are also net of the costs of implementing testing, since both targeted isolation and voluntary isolation control strategies require some level of testing and knowledge of infection status. Our focus here is on the benefits of targeted isolation strategies rather than the details of how to implement them, or on cross-regional comparisons of implemented strategies (see SI 5.5.1 for more discussion); designing such incentive mechanisms presents its own challenges, e.g.,^10,14,65^, and is an important area for future research. However, through our compliance scenarios we also demonstrate the effect of improperly implemented targeted isolation.

Given the appealing features of targeted isolation strategies, is there still a role for blanket lockdowns? Particularly when new research indicates their effects may partially be driven by voluntary isolation^50^? On the one hand, blanket lockdown strategies can reduce burdens on hospital systems, particularly in the initial phase,^58,66^, while on the other hand, the rebound effects may still induce substantial strains on hospital systems later on (SI Fig. S10). The excessive costs and rebound effects are robust features of blanket lockdowns, both in our model (Figs. S7 and S11) and confirmed in previous studies, e.g.,^11,67,68^. The rebound size in our model is also large—nearly 100% of cases averted during the blanket lockdown reoccur later on. While “targeted lockdowns” that lockdown areas or businesses burdened with higher transmission rates^69,70^ avoid some of the excess costs of blanket lockdowns, they are still blunt instruments compared to targeted isolation. Nonetheless, blanket lockdowns and targeted isolation strategies may be complementary—blanket lockdowns reduce hospitalization burdens in the early days when test quality is low, and targeted isolation manages rebound effects by correcting the coordination problem once test quality has increased. Our analysis suggests the optimal time to switch from blanket lockdowns to targeted isolation will depend critically on test quality. Finally, while ensuring compliance with targeted isolation may be more costly than ensuring compliance with blanket lockdowns, we show that for many plausible lockdown designs the targeted isolation compliance costs would have to be very large to overturn the cost savings from targeted isolation (see Figs. 5F and 2B—between $8,000 and $20,000 per person).

As vaccines are being deployed, new SARS-CoV-2 variants are now circulating in many countries^71^, and breakthrough infections have been observed. Thus it continues to be critical to avoid premature relaxation of disease-economy management measures^30^. Our results carry insights for vaccine deployment, to the extent vaccination limits infectiousness. Since our model shows that one infectious individual failing to isolate will induce many susceptible individuals to withdraw, our model insights are consistent with prioritizing vaccines to individuals who, when infectious, are least likely or able to isolate (and therefore most likely to contribute to spread). Using targeted isolation throughout vaccine delivery can further reduce economic costs and disease burden.

There remain many opportunities and open challenges in coupled-systems modeling of disease control and economy management. There is important heterogeneity in transmission, infectiousness, and exposure (e.g., superspreading events and crowding^72,73^), though explicitly incorporating such heterogeneity into the coupled systems is non-trivial. Our model applications have demonstrated one way to tractably introduce such features into a rational epidemic setting. As greater amounts of high-fidelity mobility data become available, it is important to build data-driven mappings between mobility, contacts, and economic activities within transmission models—^10,74–76^ offer promising steps in this direction. However, connecting mobility to contact rates and infection probabilities (given a contact) will require further consideration. Such extensions to the calibration methodology are essential to study disease-economy impacts of heterogeneity in individual behaviors, abilities to isolate and work from home across economic sectors, and regional policies. Finally, it is critically important to consider how to design incentives and measure the costs of implementing targeted isolation programs that can sustain participation and compliance.

As an endgame strategy, targeted isolation could avert trillions in recessionary losses while effectively controlling the epidemic. Put differently, disease-economy trade-offs are inevitable when the coordination failure cannot be resolved. The coordination failure can be resolved through positive incentives (e.g. payments to individuals to learn their disease status and isolate) or negative incentives (e.g. penalties for individuals who do not learn their disease status and isolate). Amidst the ongoing public policy debate about economic relief, lockdown fatigue, and epidemic control^26^, allocating funds to solving the coordination problem likely passes the cost-benefit test.

## Methods

Here we provide an overview of the key elements of our framework including describing the contact function that links economic activities to contacts, the SIRD (Susceptible-Infectious-Recovered-Dead) model, the dynamic economic model governing choices, and calibration. The core of our approach is a dynamic optimization model of individual behavior coupled with an SIRD model of infectious disease spread. Additional details are found in the SI.

### Contact function

We model daily contacts as a function of economic activities (labor supply, measured in hours, and consumption demand, measured in dollars) creating a detailed mapping between contacts and economic activities. For example, all else equal, if a susceptible individual reduces their labor supply from 8 to 4 h, they reduce their daily contacts at work from 7.5 to 3.75. Epidemiological data is central to calibrating this mapping between epidemiology and economic behavior. Intuitively, the calibration involves calculating the mean number of disease-transmitting contacts occurring at the start of the epidemic and linking it to the number of dollars spent on consumption and hours of labor supplied before the recession begins.

We use an SIRD transmission framework to simulate SARS-CoV-2 transmission for a population of 331 million interacting agents. This is supported by several studies (e.g.,^77,78^) that identify infectiousness prior to symptom onset. We consider three health types *m* ∈ {*S*, *I*, *R*} for individuals, corresponding to epidemiological compartments of susceptible (*S*), infectious (*I*), and recovered (*R*). Individuals of health type *m* engage in various economic activities ${A}_{i}^{m}$, with *i* denoting the activities modeled. One of the ${A}_{i}^{m}$ is assumed to represent unavoidable other non-economic activities, such as sleeping and commuting, which occur during the hours of the day not used for economic activities (see SI 2.3.1). Disease dynamics are driven by contacts between susceptible and infectious types, where the number of susceptible-infectious contacts per person is given by the following linear equation:

1 $${{{{{{{{\mathscr{C}}}}}}}}}^{SI}({{{{{{{\bf{A}}}}}}}})=\mathop{\sum}\limits_{i}{\rho }_{i}{A}_{i}^{S}{A}_{i}^{I}$$

while similar in several respects to prior epi-econ models^15,16,74^, a methodological contribution is that *ρ*_*i*_ converts hours worked and dollars spent into contacts. For example, *ρ*_*c*_ has units of contacts per squared dollar spent at consumption activities, while *ρ*_*l*_ has units of contacts per squared hour worked.

We also consider robustness to different functional forms in Fig. 6F, G as a reduced-form way to consider multiple consumption and labor activities with heterogeneous contact rates. Formally:

2 $${{{{{{{{\mathscr{C}}}}}}}}}^{SI}({{{{{{{\bf{A}}}}}}}})=\mathop{\sum}\limits_{i}{\rho }_{i}{({A}_{i}^{S}{A}_{i}^{I})}^{\alpha },$$

where *α* > 1 (convex) corresponds to a contact function where higher-contact activities are easiest to reduce or individuals with more contacts are easier to isolate. *α* < 1 (concave) corresponds to a contact function where higher-contact activities are hardest to reduce or individuals with fewer contacts are easier to isolate. The baseline case (*α* = 1) implies all consumption or labor activities and individuals have identical contact rates (See SI 2.3.2 for further discussion and intuition).

### Calibrating contacts

To calibrate the contact function, we use US-specific age and location contact matrices generated in ref. ^6^, which provide projected age-specific contact rates at different locations in 2017 (shown in SI section 2.3.1). We group these location-specific contact matrices into matrices for contacts during consumption, labor, and unavoidable other activities. The transmission rate was calibrated to give a value of ${{{{{{{{\mathcal{R}}}}}}}}}_{0}$ = 2.6, reflective of estimates^79^. For this, we use the next-generation matrix^40^. The next-generation matrix describes the “next generation” of infections caused by a single infected individual; the ${{{{{{{{\mathcal{R}}}}}}}}}_{0}$ is the dominant eigenvalue of the next-generation matrix (see SI 2.5.3). This calculation is done at the disease-free steady state of the epidemiological dynamical system, when all the population is susceptible. Specifically, we calculate the benchmark number of contacts from each activity in the pre-epidemic equilibrium (e.g., *ρ*_*c*_*c*^*S*^*c*^*I*^ for consumption from equation (1)), under pre-epidemic consumption and labor supply levels. We then calculate the coefficients *ρ*_*c*_, *ρ*_*l*_, *ρ*_*o*_ (for consumption, labor, unavoidable other) using (1) such that pre-epidemic consumption and labor supply levels equal the benchmark number of contacts. To account for contacts that are not related to economic activities, the “unavoidable other” contact category is normalized to 1, so that the coefficient *ρ*_*o*_ is simply the number of contacts associated with unavoidable other activities. While pre-pandemic contact structures are necessary to calibrate ${{{{{{{{\mathcal{R}}}}}}}}}_{0}$, our model allows contacts to evolve over time as a function of individual choices, which respond to disease dynamics.

The contact matrices in^6^ measure only contacts between individuals in different age groups by activity, without noting which individuals are consuming and which are working. Given the lack of precise data on contacts between individuals engaging in different activities, we simplify by assuming individuals who are consuming only contact others who are consuming, and individuals who are working only contact others who are working. However, in reality individuals who are consuming also interact with individuals who are working (e.g., a bar or restaurant). Future work could collect more detailed contact data describing contacts between individuals engaging in different activities.

### SIRD epidemiological model

The SIRD model is given by:

3 $${S}_{t+1} 	={S}_{t}-\tau {{{{{{{{\mathscr{C}}}}}}}}}^{SI}({{{{{{{\bf{A}}}}}}}}){S}_{t}{I}_{t},\\ {I}_{t+1} 	={I}_{t}+\tau {{{{{{{{\mathscr{C}}}}}}}}}^{SI}({{{{{{{\bf{A}}}}}}}}){S}_{t}{I}_{t}-({P}^{R}+{P}^{D}){I}_{t},\\ {R}_{t+1} 	={R}_{t}+{P}^{R}{I}_{t},\\ {D}_{t+1} 	={D}_{t}+{P}^{D}{I}_{t}.$$

Where *S*, *I*, *R*, *D* represent the fractions of the population in those compartments. Because the contact function ${{{{{{{{\mathscr{C}}}}}}}}}^{SI}({{{{{{{\bf{A}}}}}}}})$ returns the number of contacts per person as a function of activities **A**, then *τ* is a property of the pathogen that determines the infections per contact. This decomposes the classic “*β*” in epidemiological modeling into a biological component that is a function of the pathogen (*τ*) and a behavioral component linked to economic activity (${{{{{{{\mathscr{C}}}}}}}}({{{{{{{\bf{A}}}}}}}})$), such that $\beta ={{{{{{{\mathscr{C}}}}}}}}({{{{{{{\bf{A}}}}}}}})\tau$ (e.g.,^16^).

A key input into individual decision making is the probability of infection for a susceptible individual, which per the SIRD model above depends on the properties of the pathogen, contacts generated through economic activities, and the share of infectious individuals in the population:

4 $${P}_{t}^{I}=\tau {{{{{{{{\mathscr{C}}}}}}}}}^{SI}({{{{{{{\bf{A}}}}}}}}){I}_{t}.$$

If a susceptible individual reduces their activities (and thus contacts) today, they reduce the probability they will get infected, which in turn reduces the growth of the infection. However, if they keep their economic behavior the same, they enjoy those benefits today, but take the risk of becoming infected in the future. Finally, *P*^*R*^ is the rate at which infectious individuals recover, and *P*^*D*^ is the rate at which they die. Both are assumed to be constant over time and independent of economic activities and contacts.

Our framework can be generalized to other structured compartmental models beyond mean-field (homogeneous) SIRD models. The key feature to translate is the contact function. For example, in an age-structured model the contact function would need to reflect age-specific consumption and labor supply patterns.

### Choices

In order to analyze the three control strategies (voluntary isolation, blanket lockdown, targeted isolation), we solve two types of constrained optimization problems: a decentralized problem and a social planner problem. The decentralized problem reflects atomistic behavior by individuals—they aim to maximize their personal utility and make choices regarding economic activity. The decentralized problem is used to analyze the voluntary isolation and blanket lockdown strategies. Conversely, in the social planner problem, a social planner considers the utility of the population as a whole and coordinates economic activity to jointly maximize the utilities of all individuals in the population. Importantly, the social planner internalizes the full economic costs to the population associated with disease transmission. The social planner problem is used to analyze the targeted lockdown strategy.

In the decentralized problem, individuals observe the disease dynamics, know their own health state, and make consumption and labor choices in each period accounting for the risks incurred by contacts with potentially infectious individuals. Individuals’ knowledge of their own daily health state is consistent with a testing system where individuals use a daily test which reveals their health state. Let ${{{{{{{\bf{A}}}}}}}}=\{{c}_{t}^{m},{l}_{t}^{m}\}$ represent the economic activities of consumption and labor chosen in period *t* by individuals of health type *m*. Individuals maximize their lifetime utility by choosing their economic activities, ${c}_{t}^{m}$ and ${l}_{t}^{m}$, accounting for the effects of infection and recovery on their own welfare:

5 $${U}_{t}^{S}=\mathop{\max }\limits_{{c}_{t}^{S},{l}_{t}^{S}}\{u({c}_{t}^{S},{l}_{t}^{S})+\delta ((1-{P}_{t}^{I}){U}_{t+1}^{S}+{P}_{t}^{I}{U}_{t+1}^{I})\},$$

6 $${U}_{t}^{I}=\mathop{\max }\limits_{{c}_{t}^{I},{l}_{t}^{I}}\left\{u({c}_{t}^{I},{l}_{t}^{I})+\delta ((1-{P}^{R}-{P}^{D}){U}_{t+1}^{I}+{P}^{R}{U}_{t+1}^{R}+{P}^{D}{U}_{t+1}^{D})\right\},$$

7 $${U}_{t}^{R}=\mathop{\max }\limits_{{c}_{t}^{R},{l}_{t}^{R}}\{u({c}_{t}^{R},{l}_{t}^{R})+\delta {U}_{t+1}^{R}\},$$

8 $${U}_{t}^{D}={{\Omega }}\,\forall t.$$

Per-period utility $u({c}_{t}^{m},{l}_{t}^{m})$ captures the contemporaneous net benefits from consumption and labor choices. In particular, susceptible individuals in period *t* recognize their personal risk of infection ${P}_{t}^{I}$ is related to their choices regarding economic activity ${c}_{t}^{S},{l}_{t}^{S}$, and if they do become infected in period *t* + 1, they have some risk of death in period *t* + 2. Death imposes a constant utility of Ω, calibrated to reflect the value of a statistical life (see SI 2.1.3). The daily discount factor *δ* reflects individuals’ willingness to trade consumption today for consumption tomorrow.

Finally, individuals exchange labor (which they dislike), for consumption (which they do like) such that their budget balances in each period:

9 $$p{c}_{t}^{m}={w}_{t}{\phi }^{m}{l}_{t}^{m}.$$

The wage rate *w*_*t*_ is paid to all individuals, per effective unit of labor ${\phi }_{t}^{m}{l}_{t}^{m}$, and is calculated from per-capita GDP. We represent the degree to which individuals are able to be productive at work by *ϕ*^*m*^ (labor productivity). We assume that symptomatic individuals are less productive, such that *ϕ*^*S*^ = *ϕ*^*R*^ = 1 and *ϕ*^*I*^ < 1, reflecting the average decrease in productivity of infectious individuals (accounting for the share of asymptomatic and pre-symptomatic individuals, similar to^24^—see SI 2.1.1). Following standard practice, the price of consumption *p* is normalized to 1. Finally, market equations that state how individuals are embedded in a broader economy are described in the SI.

The social planner problem coordinates the economic activities of the individuals described above. Instead of economic activities being individually chosen to maximize personal utility, the social planner coordinates consumption and labor choices of each type (${{{{{{{{\bf{c}}}}}}}}}_{{{{{{{{\bf{t}}}}}}}}}={c}_{t}^{S},{c}_{t}^{I},{c}_{t}^{R}$, ${{{{{{{{\bf{l}}}}}}}}}_{{{{{{{{\bf{t}}}}}}}}}={l}_{t}^{S},{l}_{t}^{I},{l}_{t}^{R}$) to maximize the utility of the population over the planning horizon, subject to the disease dynamics (3) and budget constraints (9):

10 $$\mathop{\max }\limits_{{{{{{{{{\bf{l}}}}}}}}}_{{{{{{{{\bf{t}}}}}}}}},{{{{{{{{\bf{c}}}}}}}}}_{{{{{{{{\bf{t}}}}}}}}}}\mathop{\sum }\limits_{t=0}^{\infty }{\delta }^{t}({S}_{t}u({c}_{t}^{S},{l}_{t}^{S})+{I}_{t}u({c}_{t}^{I},{l}_{t}^{I})+{R}_{t}u({c}_{t}^{R},{l}_{t}^{R})+{D}_{t}{{\Omega }}).$$

Additional structure (e.g., age compartments, job types, geography) can be incorporated here either by creating additional utility functions or by introducing type-specific constraints. For example, with age compartments, each age type would have a set of utility functions like equations (5)–(8). These would then be calibrated to reflect age-specific economic activity levels, structural parameters, and observed risk-averting behaviors.

Both the decentralized problem and the social planner problem are solved for optimal daily consumption and labor supply choices in response to daily state variable updates, and we normalize the total initial population size to 1 for computational convenience. The assumption that individuals use a daily test that reveals their health state is maintained across both the decentralized and the social planner problems. We abstract from the cost of the testing system. Since the cost is common to both problems, it does not affect the relative comparison between the two.

### Utility calibration

Details of the utility function calibration and data sources are found in the SI. Briefly, economic activity levels and structural economic parameters are calibrated to match observed pre-epidemic variables for the US economy. We calibrate risk aversion and the utility cost of death to match the value of a statistical life. This approach ensures both the levels of economic choice variables and their responses to changes in the probability of infection are consistent with observed behaviors in other settings.

### Model applications

We add information frictions and individual non-compliance to our baseline model to study how plausible magnitudes of such distortions may affect our policy conclusions. These are modeled by altering the inputs into agents’ optimal choice rules (known as “policy functions” in dynamic optimization problems, not to be confused with pandemic control policies) that specify their (c^*^, l^*^) choice given the (*S*, *I*, *R*) information they have. The choice rules take the form shown in the equation below, where the only addition to the usual sub/superscripts is [*P*] denoting the policy type {*V*, *T*, *L*} for voluntary isolation, targeted isolation and blanket lockdown policies respectively:

11 $${c}_{[P],t}^{* S}={c}_{[P],t}^{S}({S}_{t},{I}_{t},{R}_{t})$$

12 $${l}_{[P],t}^{* S}={l}_{[P],t}^{S}({S}_{t},{I}_{t},{R}_{t})$$

all three types of agent choose consumption and labor consistent with these choice rules depending on what they know of the state of the world (i.e. (*S*_*t*_, *I*_*t*_, *R*_*t*_)). These choice rules are the main output of the value function iteration process described in SI 3.1. By feeding different information into the choice rules or taking weighted averages under different policies, we can model the frictions described below as different scenarios.

#### Test reporting lags

Test reporting lags force agents to react to population-level infection information from *x* days ago. This is modeled as feeding (*S*_*t*−*x*_, *I*_*t*−*x*_, *R*_*t*−*x*_) into the choice rules above when finding $({c}_{t}^{* },{l}_{t}^{* })$. We select *x* to be roughly consistent with observed lags during the COVID-19 pandemic: initially 8 days at the outset of the pandemic, before falling to 5 days on day 60 and 3 days at day 75. The choice rules become:

13 $${c}_{[P],t}^{* S}={c}_{[P],t}^{S}({S}_{t-x},{I}_{t-x},{R}_{t-x})$$

14 $${l}_{[P],t}^{* S}={l}_{[P],t}^{S}({S}_{t-x},{I}_{t-x},{R}_{t-x})$$

#### Test quality

Tests for individual health status differ in quality throughout the course of a novel pandemic, starting from very low quality before becoming progressively more accurate. We assume that due to test quality *q* (for the specific foundation of this single-metric quality notion related to specificity and sensitivity see SI 3.3.2), individuals take a weighted average of the choice-rule-prescribed action for their true health type and a “no information” action which is averaged uniformly across the actions for each type. This is equivalent to either of the following behavioral microfoundations:

- individuals realize they do not know their type with certainty, so can do no better than using *q* to mix between the choice-rule-prescribed action for their test-reported type and an average across actions for each of the three types; or
- a fraction *q* of agents of a given type trust their test result and follow the associated choice-rule-prescribed action, while the remaining 1 − *q* fraction either do not get tested or do not trust their test and uniformly mix across actions for all health types.

We consider two types of test quality scenarios: first a “limited testing” scenario where test quality is low throughout the whole pandemic, and second a more-realistic “improving test quality” scenario where test quality linearly improves over the course of the pandemic, becoming perfect at day 75. The choice rules become:

15 $${c}_{[P],t}^{* S}=q{c}_{[P],t}^{* S}+(1-q)\left(\frac{1}{3}{c}_{[P],t}^{* S}+\frac{1}{3}{c}_{[P],t}^{* I}+\frac{1}{3}{c}_{[P],t}^{* R}\right)$$

16 $${l}_{[P],t}^{* S}=q{l}_{[P],t}^{* S}+(1-q)\left(\frac{1}{3}{l}_{[P],t}^{* S}+\frac{1}{3}{l}_{[P],t}^{* I}+\frac{1}{3}{l}_{[P],t}^{* R}\right)$$

we examine the robustness of our conclusions to an equilibrium model of behavior under low-quality information or limited cognitive capacity in SI 3.3.4, finding that the qualitative results regarding policy effectiveness are unchanged.

#### Compliance

Some individuals may not comply with policy mandates. We model this as a share of agents $\bar{c}$ of any type that choose the decentralized (i.e. voluntary isolation) action rather than complying with the targeted isolation or blanket lockdown mandates. We consider two types of scenarios, “low compliance” with 10% compliance and “partial compliance” with 75% compliance. The choice rules become:

17 $${c}_{[P],t}^{* S}=\bar{c}* {c}_{[P],t}^{* S}+(1-\bar{c})* {c}_{[D],t}^{* S}$$

18 $${l}_{[P],t}^{* S}=\bar{c}* {l}_{[P],t}^{* S}+(1-\bar{c})* {l}_{[D],t}^{* S}$$

### Reporting summary

Further information on research design is available in the Nature Research Reporting Summary linked to this article.

## Supplementary information

Supplementary Information

Reporting Summary

## Data Availability

All data for replicating the results in this paper can be found at https://github.com/epi-econ/COVID19_ControlStrategies^80^.

## Code availability

All code for replicating the results in this paper can be found at https://github.com/epi-econ/COVID19_ControlStrategies^80^. All simulations were run in R.
