# Author Correction: Disease-economy trade-offs under alternative epidemic control strategies

**DOI:** 10.1038/s41467-022-31307-2

**Published:** 2022-06-22

**Authors:** Thomas Ash, Antonio M. Bento, Daniel Kaffine, Akhil Rao, Ana I. Bento

**Affiliations:** 1 Department of Economics, University of Southern California, Los Angeles, CA 90007 USA; 2 Sol Price School of Public Policy, University of Southern California, Los Angeles, CA 90007 USA; 3 National Bureau of Economic Research, Cambridge, MA 02138 USA; 4 Department of Economics, University of Colorado Boulder, Boulder, CO USA; 5 Department of Economics, Middlebury College, Middlebury, VT USA; 6 Department of Epidemiology & Biostatistics, School of Public Health, Indiana University, Bloomington, IN USA; 7 Pandemic Prevention Institute, Rockefeller Foundation, New York, NY USA

**Keywords:** Environmental economics, Population dynamics, Epidemiology, SARS-CoV-2

Correction to: *Nature Communications* 10.1038/s41467-022-30642-8, published online 09 June 2022.

In this article the primary affiliation Sol Price School of Public Policy, University of Southern California, Los Angeles, CA, 90007 and the National Bureau of Economic Research, Cambridge, MA 02138 affiliation for Antonio M. Bento were missing. The original article has been corrected.

