# Supplementary material for: Disease-economy trade-offs under alternative epidemic control strategies
Source: Nat Commun. 2022 Jun 9;13:3319. doi: 10.1038/s41467-022-30642-8 (PMC9178341; doi:10.1038/s41467-022-30642-8)
Supplement: Supplementary file 1 — Supplementary Information [file 41467_2022_30642_MOESM1_ESM.pdf]

Supporting Information for "Disease-economy trade-offs under alternative epidemic control strategies"

Thomas Ash, Antonio M. Bento*, Daniel Kaffine, Akhil Rao and Ana I. Bento*

April 23, 2022

Contents

1 Model overview **2**

2 Model components and calibration **3**

2.1 Utility functions . . . 3

2.1.1 Productivity and asymptomatic/pre-symptomatic individuals . . . 4

2.1.2 Risk aversion . . . 6

2.1.3 Value of a Statistical Life (VSL) . . . 7

2.2 Markets . . . 9

2.3 Disease dynamics . . . 9

2.3.1 Contact rates . . . 10

2.3.2 The shape of the contact function . . . 11

2.3.3 The next-generation matrix method . . . 13

2.3.4 The reproductive number . . . 14

2.4 Solution concepts . . . 14

2.5 Control strategies and trade-offs . . . 16

2.6 Individual heterogeneity and coordination failures . . . 19

2.7 Parameter values . . . 23

2.8 Empirical validation to observed US economic outcomes during COVID-19 . . . 23

3 Solving the model **25**

3.1 Solution methods and algorithms . . . 26

3.1.1 Grid construction . . . 29

3.2 Correctly representing atomistic individual behavior . . . 29

3.3 Model applications . . . 31
3.3.1 Information frictions: information delays . . . 31
3.3.2 Information frictions: test quality . . . 32
3.3.3 Partial compliance . . . 34
3.3.4 Equilibrium responses to limited test quality . . . 34

4 Sensitivity to parameter variations **38**
4.1 Sensitivity to blanket lockdown implementation . . . 38
4.2 Sensitivity to average case fatality rate (CFR) . . . 41
4.3 Sensitivity to income per capita . . . 41
4.4 Sensitivity to VSL . . . 43
4.5 Sensitivity to risk aversion . . . 43

5 Discussion **44**
5.1 Implications for vaccine delivery . . . 44
5.2 Implied hospitalization rate . . . 45
5.3 Implications of blanket lockdowns on hospitalization rates . . . 47
5.4 Possibility of an economy-wide structural change towards more online activity . . . 48
5.5 Additional considerations . . . 52
5.5.1 Cross-regional comparisons . . . 52
5.5.2 Different economic sectors . . . 52
5.5.3 Behavioral adjustment costs . . . 52
5.5.4 Alternative social welfare functions . . . 53

1 Model overview

We calculate the economic costs and incidence (infected individuals per 100,000) under different control strategies in three steps. First, we calibrate the utility functions, budget constraints, and contact functions to match pre-epidemic economic and contact data. We choose parameters governing the disease dynamics based on estimates from the literature on COVID-19. All calibrated parameters can be found in Tables S3 and S4. Using the calibrated functions, we solve the model and generate daily time paths of the economic and disease outcomes under voluntary isolation, targeted isolation, and blanket lockdowns for approximately 1.5 years (548 days). Finally, we calculate the economic costs as a discounted sum of consumption losses under each scenario and the cases per 100,000 from the final state of each scenario.

This coupled-systems model has two core components:

1. **Utility functions and budget constraints:** Individuals choose to maximize their utility functions subject to their budget constraints. A key component of individuals' decision-making is that they choose activity levels with the awareness of the probability they get infected at different activities, and respond accordingly. Their choices determine the probability of infection at different activity sites.

2. **Disease dynamics:** The disease evolves as a function of individuals' choices and the prevalence of individuals with different disease statuses. These in turn feed back into individuals' choices.

2 Model components and calibration

2.1 Utility functions

The individual per-period utility function $u(c,l)$ in equations 3-5 consists of several pieces. First, the per-period utility from consumption and labor $\hat{u}(c,l)$ has the following functional form:

$$\hat{u}(c,l) = ((\alpha^{\frac{1}{\sigma}} c^{\frac{\sigma-1}{\sigma}} + (1-\alpha)^{\frac{1}{\sigma}} (1-l)^{\frac{\sigma-1}{\sigma}})^{\frac{\sigma}{\sigma-1}})(1+\upsilon). \quad (1)$$

This function represents individual preferences over the economic activities of consumption and leisure/labor and is a standard Constant Elasticity of Substitution (CES) utility function. Individuals value consumption of goods and services c and their leisure (non-labor) time $(1-l)$. This functional form allows us to calibrate how much the individual values consumption relative to leisure (α) and the elasticity of substitution between consumption and leisure (σ). υ measures the utility of unavoidable other contacts, such as those occurring at home. Since unavoidable other contacts do not vary over time and are common to all individuals and health types, we normalize υ to 0 without loss of generality.

Finally, individuals are averse to uncertainty (risk aversion)—they are willing to exchange things they value (e.g., consumption) to reduce the uncertainty associated with becoming infected. Note that this is distinct from their preference for not getting infected; while individuals prefer to remain healthy, risk aversion captures their preference for certainty. In economic terminology, individuals wish to "consumption smooth" across susceptible, infected and other states of the world. This is modeled via the standard Constant Relative Risk Aversion (CRRA) functional form. Putting

equation 1 into the CRRA format gives the full per-period utility function as:

$$u(c,l) = \frac{\hat{u}(c,l)^{1-\eta} - 1}{1-\eta} \tag{2}$$

The risk-aversion parameter η plays an important role in economic models and governs the degree of risk-averting behavior individuals undertake. If $\eta < 0$, individuals seek out the uncertainty associated with risks; if $\eta = 0$, individuals are indifferent to uncertainties associated with risks; and if $\eta > 0$ individuals try to avoid the uncertainties associated with risks. Following a long literature on risk aversion (e.g., [1]), we take $\eta > 0$ in all model simulations. We discuss this further in section 2.1.2.

We perform a grid search to calibrate utility parameters (σ and α) and the hourly wage (w) so as to reproduce benchmark values of consumption demand (c^* in Table S3), labor supply (l^* in Table S3), and uncompensated wage elasticity of labor supply (0.15, consistent with the compensated elasticities of broad income and income effects in [2]). In calibrating the daily labor supply, we assume time spent sleeping is not counted as leisure. The yearly consumption calibration is annuitized to a daily scale, implying individuals are also making labor-leisure tradeoffs on weekends to facilitate consumption.

We set the daily endowment of time usable in consumption, labor, and leisure to 12 hours. The rest of the day is assumed spent sleeping, commuting, and performing other unavoidable non-economic activities. This implies that in the pre-epidemic equilibrium, 8 hours of the day are spent working and the remaining 4 hours are allocated to consumption and leisure. Unavoidable other contacts are assumed to occur during the remaining 12 hours of the day which are not usable for economic activities.

2.1.1 Productivity and asymptomatic/pre-symptomatic individuals

We do not explicitly model asymptomatic status, pre-symptomatic stages, or mild infections in individuals. We assume infected individuals are unable to work only if they present severe symptoms. Infected individuals who are asymptomatic, pre-symptomatic, or have mild infections, consume and work as though they have no knowledge they are infected.[1] The pathogen-specific productivity parameter ϕ_I converts hours worked by average infected individuals into output. We assume

[1] To emphasize, all individuals in our baseline model are aware of their infection status. It has been shown that a vast majority of transmission events occurs with asymptomatic/pre-symptomatic individuals [3, 4]; with full information there is no distinction between symptomatic and asymptomatic/pre-symptomatic infectious individuals in the compartmental structure of the model.

only asymptomatic/pre-symptomatic individuals and those experiencing mild symptoms are able to work, so that ϕ_I reflects the share of the infected population which is able to work at any given time. Thus, ϕ_I determines the quantity of asymptomatic individuals for a given pathogen.

We calculate ϕ_I as a duration-weighted average of time spent in states of differing infection severity. Letting the proportion of the population which is asymptomatic or pre-symptomatic be P_a, the proportion of minor sufferers be P_m, the proportion of major sufferers be P_M, the proportion of infected time spent symptomatic be t_s, and the proportion of infected time spent asymptomatic or pre-symptomatic be t_a, this yields

$$\phi_I = 1 * (P_a + P_m) + (0 * t_s + 1 * t_a) * P_M. \tag{3}$$

Assuming major sufferers are hospitalized gives $P_M = H$ (the percentage of those infected that are hospitalized), and the proportion of infected time spent asymptomatic is $t_a = d_a/d_h$; d_a is the duration spent asymptomatic or pre-symptomatic and d_h is the overall duration infected for an individual that is hospitalized. Applying the values from Table S4 gives $P_M = 0.199$, $P_a + P_m = 1 - 0.199$, $t_a = 0.2737$, $t_s = 1 - 0.2737$, giving the average daily productivity while infected as $\phi_I = 0.8555$.

Note that equation 3 can imply negative asymptomatic and minor sufferer shares when ϕ_I falls below t_a, or shares greater than 1 when ϕ_I goes above $1 - t_a$. That is, inverting equation 3 yields

$$P_a + P_m = \frac{\phi_I - t_a}{1 - t_a}. \tag{4}$$

Given our strategy to calibrate the productivity while infected, the physically-plausible range over which ϕ_I can vary is therefore $[t_a, 1]$, or $[0.2737, 1]$—implying a productivity *loss* that must be in $[0, 0.7263]$.

Negative shares of asymptomatic/ pre-symptomatic and minor sufferers are possible because the share of time spent asymptomatic or pre-symptomatic (t_a) does not vary smoothly with the share of individuals who are likely to transit to that state ($P_a + P_m$). While the share of individuals *currently* in the asymptomatic/pre-symptomatic or minor sufferer states will depend on the share of time spent in those states, P_a and P_m are the probabilities of transiting to those states, not the proportion of individuals in them. To avoid the problem of negative shares, the time spent in asymptomatic/pre-symptomatic or minor sufferer states must go to 0 when the proportion of pre-symptomatic or asymptomatic and minor sufferers are 0 (i.e., $P_a + P_m = 0 \implies t_a = 0$), and vice

versa (i.e., $P_a + P_m = 1 \implies t_a = 1$). Given the current lack of knowledge about how the probability an individual is asymptomatic or a minor sufferer relates to the time they are likely to spend asymptomatic, more detailed functional relationships would require speculation. Future work better mapping the structural features of COVID-19 will be necessary to construct such relationships.

2.1.2 Risk aversion

Risk aversion is the extent to which individuals dislike uncertainty when making choices. For example, risk-averse individuals would be willing to accept a lower payoff overall if it meant they were to face no COVID risk whatsoever. The degree to which individuals have this preference for no risk is governed by the parameter η (the coefficient of relative risk aversion) in equation 2. The sensitivity of model projections to η is explored in section 4.5.

Calibrating η for daily-level decisions under risks with potentially severe stakes (e.g., a probability of death) and probabilities that change daily presents many challenges. The most notable is the "calibration theorem" established in [5]: appreciable degrees of risk aversion over moderate stakes implies near-risk neutrality when the stakes are small, and unrealistically high degrees of risk aversion over larger stakes. That is, a moderate coefficient of relative risk aversion (e.g., η of 1) does not fit a pandemic setting where extreme negative outcomes (e.g., death) exist and the daily probability of infection may vary over orders of magnitude. Consequently, we do not use a coefficient of relative risk aversion near 1, which is consistent with annual or quarterly decision-making and common in the literature on financial risks [6].

Instead, we use a coefficient of relative risk aversion of 0.1 to reflect low levels of risk aversion at a daily scale. We argue such a low value of risk aversion at a daily scale is plausible for two reasons. First, as shown in [6], the coefficient of relative risk aversion is related to the ratio of the income effect to the price effect. Our low η therefore implies that at the daily level, income effects are smaller relative to price effects than they are at a quarterly or annual frequency. Intuitively, this means daily changes in hours worked are more sensitive to wage changes than to changes in unearned income.

Second, research shows that a lower η of 0.1 for daily decisions is consistent with higher η of 1 for less frequent, less familiar decisions (e.g., financial decisions). [1] finds that less-familiar commitments induce local risk aversion (over moderate-scale income fluctuations) which is an order of magnitude larger than global risk aversion (over large-scale income fluctuations). Infection and death are potentially large-scale income fluctuations. This justifies a lower η for daily decisions which could result in infection and death as consistent with a higher value of η for less-frequent,

lower-stakes decisions around financial risks.

However, while we use a low value of η based on the arguments above, the exact value of η does not qualitatively change our results. We calibrate the utility of death to obtain a Value of a Statistical Life (VSL) of $10 million USD (consistent with [7] and [8]). As shown in equation 11, for any plausible value of η there exists a utility of death which achieves the target VSL. That is, it is η **and** the utility of death (the size of the stakes) that jointly determine the resulting risk-averting behavior. Thus for any plausible choice of η, by setting the utility of death to obtain a desired VSL, we are able to calibrate the model to reproduce observed trade-offs between consumption and risk of death.

2.1.3 Value of a Statistical Life (VSL)

Since the relevant risk of death is faced by infected individuals, we calibrate the utility associated with death so that infected individuals exhibit a VSL of $10 million, which is consistent with recent estimates from choices to trade risk of death for money in other settings [7, 8]. Since infected individuals are the ones directly facing the risk of death, the utility parameters reflecting VSL for infectious diseases such as COVID ought to be calibrated using the infected individuals' lifetime utility. This then influences the susceptible individuals' choices through the probability of transitioning to the infected state. We approximate the number of life-days remaining for the average individual in each state as infinity. The lifetime utility of being recovered is

$$U^R = \sum_{t=0}^{\infty} \beta^t u(c_R, l_R) \tag{5}$$

$$= \frac{u(c_R, l_R)}{1-\beta}. \tag{6}$$

Let the utility associated with death due to infection be a constant, Ω.[2] The lifetime utility of being infected is as below. It is composed of the utility obtained today, while infected, plus the discounted remaining lifetime payoffs if another state such as recovered or death is entered. Each

[2]While the utility associated with living states can be measured using choices between streams of consumption in different states, the utility associated with death is more difficult measure. Preferring a particular stream of consumption over death indicates an upper bound, and preferring to die rather than experience a lower stream of consumption indicates a lower bound. Repeated rejections of consumption streams in favor of death can provide more precise estimates but can be hard to observe.

of these are weighted by their probabilities of occurring in that period, e.g., P^R, P^D, etc.

$$U^I = u(c_I, l_I) + \beta \left[P^R U^R + P^D \Omega + (1 - P^R - P^D) U^I \right] \tag{7}$$

$$U^I - \beta(1 - P^R - P^D) U^I = u(c_I, l_I) + \beta \left[P^R U^R + P^D \Omega \right] \tag{8}$$

$$U^I = \frac{u(c_I, l_I) + \beta \left[P^R U^R + P^D \Omega \right]}{1 - \beta(1 - P^R - P^D)} \equiv U^I(c_I, P^D, \Omega). \tag{9}$$

The VSL is a ratio of additional consumption and additional risk of death, $\frac{\Delta c}{\Delta p}$, satisfying the following equation. This follows since the monetary value of life is inferred from how much individuals are willing to pay (measured as a reduction in consumption, Δc) to avoid an increased chance of death (Δp).

$$U^I(c + \Delta c, P^D + \Delta p, \Omega) = U^I(c_S, P^D, \Omega). \tag{10}$$

A VSL of \$10 million implies a compensation of \$1,000 for an additional 1/10,000 chance of death. We then set the utility associated with death, Ω, to be consistent with this:

$$\Omega : U^I(c_I + 1000, P^D + 1/10000, \Omega) = U^I(c_I, P^D, \Omega). \tag{11}$$

The Ω which satisfies equation 11 will depend on utility parameters, including the coefficient of relative risk aversion. With $\eta = 0.1$ the utility of death implied by a VSL of \$10m is approximately $-672,827$ utils. For any plausible value of η (between 0 and 1.25, based on elasticity estimates in [6]), there exists a value of Ω which satisfies equation 11.

The dependence between η and Ω can be striking. To see the implications, consider the difference between the VSL implied by using $(\Omega, \eta) = (0, 1)$ (a commonly-used combination in this literature, e.g., [9]) and the VSL implied by using $(\Omega, \eta) = (0, 0.1)$. In the former case, the VSL implied by solving equation 10 for Δc (given $\Delta p = 1/10000$) is around \$174.5m. In the latter, the implied VSL is around \$2.6m. While neither is near the range of recent VSL estimates, the VSL produced by $(\Omega, \eta) = (0, 1)$ is an order of magnitude larger than the current consensus estimate of \$10m. We explore the implications of this dependence for individual recessionary losses averted and case ratios between the decentralized and planner's management in section 4.5.

2.2 Markets

Individuals in the model are embedded in a broader economy. Similar to [9] and other models of choice in general equilibrium, we assume individuals in this economy supply labor, which is used to produce a good which they consume. Markets clear such that:

$$\begin{aligned} C_t &= \theta L_t \\ \Pi_t &= C_t - w_t L_t = 0 \\ S_t c_t^S + I_t c_t^I + R_t c_t^R &= C_t \\ S_t l_t^S + I_t l_t^I + R_t l_t^R &= L_t \end{aligned} \tag{12}$$

The first two equations in 12 represent standard economic assumptions that firms demand labor L_t to convert into consumption goods C_t at rate θ, and that firms sell those goods and pay for labor until zero economic profits are realized. Since markets are competitive, $w = \theta$ for healthy workers and $w = \phi_I \theta$ for infected workers (i.e., workers are paid their marginal product, and healthy workers are on average more productive than infected workers).

The final two equations in 12 state that total consumption demanded across health types ($\sum_{h\in\{S,I,R\}} h_t c_t^h$) equals total consumption supplied (C_t), and that total labor supplied across health types ($\sum_{h\in\{S,I,R\}} h_t l_t^h$) equals total labor demanded (L_t).

2.3 Disease dynamics

Disease dynamics follow a homogeneous (mean-field) SIRD compartmental model, where individuals transition from susceptible (S) to infected/infectious (I) states if they get infected by contact with an infected person, and from infected either to recovered (R) or dead (D) states. The parameter values for the SIRD model are drawn from the literature, and reflect mean estimates for COVID-19. The SIRD model is given by:

$$\begin{aligned} S_{t+1} &= S_t - \tau \mathscr{C}^{SI}(\mathbf{A}) S_t I_t, \\ I_{t+1} &= I_t + \tau \mathscr{C}^{SI}(\mathbf{A}) S_t I_t - (P^R + P^D) I_t, \\ R_{t+1} &= R_t + P^R I_t, \\ D_{t+1} &= D_t + P^D I_t. \end{aligned} \tag{13}$$

To calibrate the spread of the disease, we set two types of parameters: the contact rates at activities (one parameter per activity), and the number of infections per contact (a single parameter across all activities). We initialize our model with one infected individual per million. We

use parameter estimates of the infection as listed in Table S3, in particular using estimates from [10, 11] and mortality data for the United States from the Centre for Evidence-based Medicine [12].

2.3.1 Contact rates

The contact function converts economic activity levels (e.g., amounts of consumption and labor) between susceptible and infected individuals into daily contacts (number of people encountered in the course of these activities, representing opportunities for infection). This function provides a ready-to-calibrate mapping between economic choices and the resulting outcomes relevant for disease transmission (contacts), and is the key methodological innovation of this paper. Re-writing equation 1 in the main text:

$$\mathscr{C}^{SI}(c,l,o) = \rho_c c_S c_I + \rho_l l_S l_I + \rho_o. \tag{14}$$

The function has three parameters: ρ_c (contacts per squared dollar spent), ρ_l (contacts per squared hour worked), ρ_o (daily unavoidable contacts). For example, suppose an infected individual consumes \$10 in a given day and a susceptible individual consumes \$20. The product of these two provides a measure of how frequently these two groups interact in the course of the consumption activity. The parameter ρ_c converts this into units of contacts. These parameters are common for contacts between any two types, e.g., between susceptibles or between susceptible and infected individuals. We set these parameters to reflect the age-structured daily average contacts from the matrices in [13] as described below.

The matrices in [13] are synthetic contact matrices that estimate age- and location-specific contacts for several countries. We use the matrices generated for the US population in 2017. They cover contacts at work, school, home, and other sites for individuals in 5 year intervals 0-80 then 80 and older. We classify "work" and "school" contacts as occurring during labor activity, "other" contacts as occurring during consumption activity, and "home" contacts as the daily unavoidable residual contacts. We classify "school" contacts as labor since it is a work site for teachers, staff, and other workers.[3] Using synthetic contact matrices allows us to reflect the additional contacts and mixing induced by students as described below. Figure S1 shows the consumption, labor, and unavoidable contact matrices we construct from those given in [13].

[3]It seems reasonable to determine the classification of school sites by the way employees relate to them. Students typically do not attend school when on-site school employees are absent, while school employees do show up at school premises when students are absent.

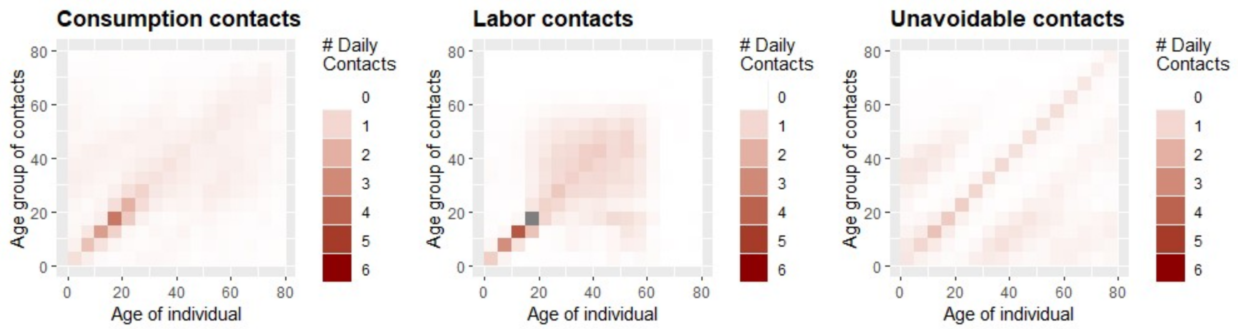

Figure S1: Contact matrices: daily contacts by economic activity and by age group. Constructed from [13].

In the pre-epidemic equilibrium, the number of contacts between two susceptible individuals (since at this point no infected individuals exist) at consumption sites, labor sites, and unavoidable activities are

$$\mathscr{C}^{SS}(c) = \rho_c c_S c_S \tag{15}$$
$$\mathscr{C}^{SS}(l) = \rho_l l_S l_S \tag{16}$$
$$\mathscr{C}^{SS}(o) = \rho_o. \tag{17}$$

We estimate pre-epidemic $C^{SS}(a), \forall a \in \{c,l,o\}$ using the matrices from [13] and set pre-epidemic c_S, l_S from our calibration in section 2.1. Using these, we calculate the contact coefficients ρ_c, ρ_l, ρ_o consistent with pre-epidemic economic activity levels and contact rates. Movement of the model's key mechanism, the mapping between economic choices and contacts, is therefore conditioned on relevant and detailed epidemiological data.

We use the next generation matrix method to calculate the mixing-adjusted average number of contacts (e.g., $C^{SS}(c)$) at each type of site as the dominant eigenvalue of the sum of the associated matrices. This approach ensures the contact rates are epidemiologically appropriate for calibrating $\mathscr{R}_0$. In section 2.3.3 we provide a brief primer on this approach—we refer readers to [14] for a more thorough discussion of the method.

2.3.2 The shape of the contact function

Our main results assume a standard linear contact function, shown in equation 14, which implies all activities and individuals have the same rate of contacts per unit of activity. However, individual heterogeneity in contact patterns by activity can affect both disease transmission and economic outcomes. For example, superspreading events occur when specific activities or individuals lead

to much higher degrees of transmission than others. As described in the Materials and Methods section of the main text, we vary the shape of the contact function in order to model such heterogeneity. The shapes we consider are described by equation 18.

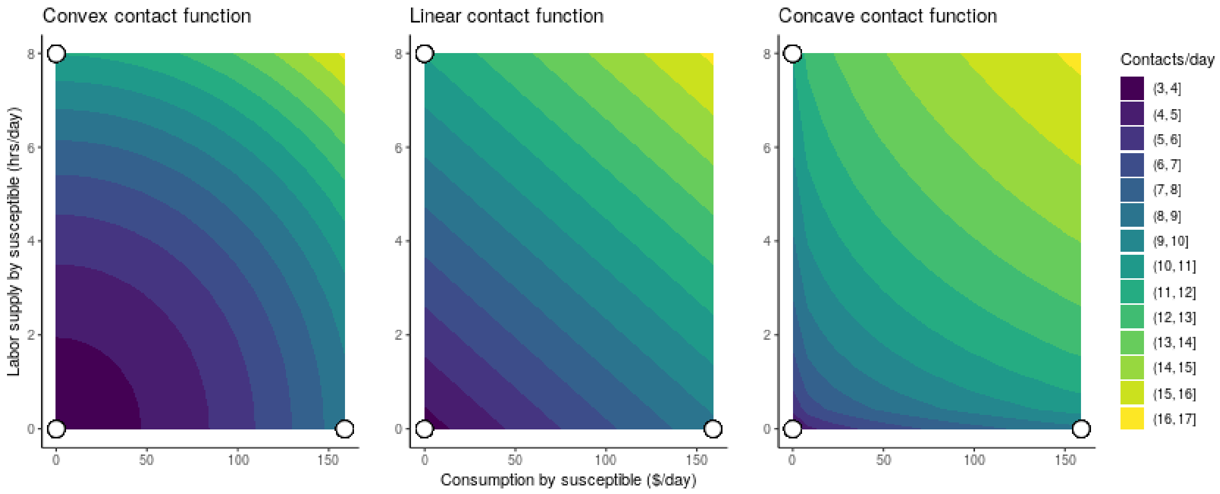

Figure S2: Calibrated contact surfaces showing the mapping between economic activity levels and contacts per day. The three white points show where contact matrix data (shown in Figure S1) was used to calibrate the surfaces. Consumption data was used to calibrate the point in the lower right, labor supply data the point in the upper left, and data at unavoidable activities used to calibrate the point at the origin.

$$\mathscr{C}^{SI}(\mathbf{A}) = \rho_c(c^S c^I)^\alpha + \rho_l(l^S l^I)^\alpha + \rho_o. \tag{18}$$

Equation 14 is the case when $\alpha = 1$. The contact function is convex when $\alpha > 1$, and concave when $\alpha < 1$. Either convex or concave forms can represent heterogeneity across activities or individuals, where a few activities or individuals account for the majority of contacts (e.g., a power law distribution of individual contacts). The degree of heterogeneity is captured in how far α is from 1. The key distinction between the convex and concave forms is how the individuals or activities are prioritized for isolation/reduction. A concave form implies high-contact activities or individuals are the last ones to be reduced or isolated, while a convex form implies high-contact activities or individuals are the first ones to be reduced or isolated.

If the costs of isolating individuals or reducing activities were homogeneous, then a social planner would likely prioritize isolation or reduction, inducing a convex contact function. As shown in the main text, such prioritization would increase the gains from targeted isolation. Intuitively, this result arises because with a convex contact function any given reduction in contact levels can be achieved with lower levels of isolation or activity reduction than under a linear contact function.

Calibrating the exponent α in equation 18 is challenging. As shown in figure S2, the pre-pandemic data points we observe can be fit equally well by many surfaces. Additional data on contacts at lower levels of activity prior to the pandemic is required to calibrate α. Such data could be obtained by incorporating questions about consumption and labor supply into contact surveys. More importantly, however, the calibration of all contact function parameters will likely need to change over time in order to reflect pandemic-induced changes in the structure of the economy.

Structural changes in the economy in response to the pandemic—such as increasing use of contactless delivery and drop-off, staggered time use of shared office spaces, and the availability of "quarantine hotels"—are likely to shift, tilt, or deform the contact function. To the extent that the pre-pandemic economy features uniformly more contacts, the use of pre-pandemic data (regardless of how many points are used) is likely to overstate the number of contacts associated with higher levels of activity. This is an instance of the Lucas Critique [15]—data prior to a structural change will conflate the impact of policies addressing the change with the effects of the change itself. While we study the sensitivity of our conclusions to such variations in Figure 4 of the main text, we note that correcting for such bias requires either ongoing contact survey data collection throughout the pandemic or a theoretically-principled way of allowing the contact parameters $\rho_c, \rho_l, \rho_o, \alpha$ to vary over time.

2.3.3 The next-generation matrix method

The term "next-generation" comes from the use of "generations" to describe waves of secondary infections which flow from each prior infection. Thus if $\mathscr{R}_t$ describes the reproduction number of the tth generation, then $\mathscr{R}_0$ is simply the number of infections generated by the first case (i.e., generation zero) in an entirely-susceptible population. Defining $\mathscr{R}_0$ in a structured compartmental epidemic model involves calculating the expected number of new infections across all infection types. Though we only use a single type of infected individual in our SIR(D) model, this method allows us to account for individuals with different ages and at different activities.

Assume we have a system with multiple discrete types of infected individuals - e.g., male, female, age classes, high risk low risk. We define the *next-generation matrix* as the square matrix $\mathbf{G}$ in which the i, jth element of $\mathbf{G}$, g_{ij} is the expected number of secondary infections of type i caused by a single infected individual of type j assuming the population of type i is entirely susceptible. That is, each element of $\mathbf{G}$ is a reproduction number which accounts for who infected whom.

The next-generation matrix has a number of desirable mathematical properties. In particular, since it is a non-negative square matrix, it is guaranteed to have a unique eigenvalue which is pos-

itive, real, and strictly larger than all others. This dominant eigenvalue (or spectral radius) is the basic reproduction number, $\mathscr{R}_0$.

To illustrate, consider a simplified directly transmitted disease in a completely susceptible population with two groups, young and old. Define y as the expected number of infected young individuals and o as the expected number of infected old individuals, given contact with a single infected member of the other group. The next-generation matrix is then

$$\begin{bmatrix} y & 0 \\ 0 & o \end{bmatrix}$$

and $\mathscr{R}_0$ is $\sqrt{yo}$. It is worth noting that this is also the geometric mean of the expected number of young and old secondary cases.

2.3.4 The reproductive number

To set the initial reproduction number $\mathscr{R}_0$, we calculate the number of infections per contact, τ, such that

$$\mathscr{R}_0 = \tau \mathscr{C}^{SI}(c,l,o)d, \tag{19}$$

where d is the mean serial interval and $\mathscr{R}_0$ is the target reproductive number. We initialize our model with the initial prevalence, ε, at one in a million. The transmission rate τ is tuned using the next-generation matrix implied by the calibrated contact function (equation 14) to give a target value of $\mathscr{R}_0$ of 2.6, consistent with estimates [16, 17].

2.4 Solution concepts

We apply two solution concepts: an "equilibrium" and an "optimal plan". As shown in Materials and Methods, the constrained maximization problems which susceptible, infected, and recovered individuals solve in each period in the decentralized cases (voluntary isolation and blanket lockdown) are

$$U_t^S = \max_{c_t^S, l_t^S} \{u(c_t^S, l_t^S) + \delta((1-P_t^I)U_{t+1}^S + P_t^I U_{t+1}^I)\}, \tag{20}$$
$$\text{s.t. } pc_t^S \leq w^S l_t^S$$

$$U_t^I = \max_{c_t^I, l_t^I} \{u(c_t^I, l_t^I) + \delta((1-P^R-P^D)U_{t+1}^I + P^R U_{t+1}^R + P^D U_{t+1}^D)\}, \tag{21}$$
$$\text{s.t. } pc_t^I \leq w^I l_t^I$$

$$U_t^R = \max_{c_t^R, l_t^R} \{u(c_t^R, l_t^R) + \delta U_{t+1}^R\}, \tag{22}$$
$$\text{s.t. } pc_t^R \leq w^R l_t^R$$

The utility of dead individuals is simply

$$U_t^D = \Omega \quad \forall t. \tag{23}$$

The planner's problem is

$$\max_{\mathbf{l_t}, \mathbf{c_t}} \sum_{t=0}^{\infty} \delta^t (S_t u(c_t^S, l_t^S) + I_t u(c_t^I, l_t^I) + R_t u(c_t^R, l_t^R) + D_t \Omega) \tag{24}$$
$$\text{s.t., } \forall t, pc_t^S \leq w^S l_t^S \tag{25}$$
$$pc_t^I \leq w^I l_t^I \tag{26}$$
$$pc_t^R \leq w^R l_t^R. \tag{27}$$

All of the maximization problems are also subject to the disease dynamics (system of equations 13).

An equilibrium is a vector of choices for each type, (c^S, l^S),(c^I, l^I),(c^R, l^R), such that individual utilities are maximized subject to budget constraints and disease dynamics, and markets clear (i.e., a joint solution to the systems in 20, 21, 22, 23, subject to system 13 and total consumption equalling total production: $C_t = \theta L_t$, as shown in system of equations 12). This equilibrium is the voluntary isolation strategy. When we study the blanket lockdown scenario, equilibrium behavior is used to generate disease and economic dynamics while the lockdown doesn't bind. Section 4.1 describes how the blanket lockdown scenario is constructed in more detail.

An optimal plan is a vector of choices for each type, $(c^S, l^S, c^I, l^I, c^R, l^R)$, such that aggregate population utility is maximized subject to budget constraints and disease dynamics, and markets clear (i.e., a solution to the system in 24, subject to system 13 and total consumption equalling total production). This optimal plan is the targeted isolation scenario.

The coordination failure is precisely the difference between solving the decentralized problems (which yield the equilibrium) and solving the social planner's problem (which yield the optimal plan). The equilibrium involves each individual type choosing their labor supply and consumption demand having accounted for their personal benefits and costs from those choices, and ignoring how their choices impact others. The optimal plan instead has the planner coordinating individuals' choices to maximize their collective utility, i.e. accounting for how behavior by one impacts benefits and costs received by others.

There is no coordination failure (i.e., no difference between behavior in the equilibrium vs the optimal plan) for recovered individuals because they neither contribute to disease spread nor are impacted by contact with infectious individuals. Infectious and susceptible individuals are where the differences arise. Infectious individuals' equilibrium behavior ignores how their presence in common spaces impacts susceptible individuals, who withdraw activity in response to infection risk. Since susceptible individuals are the majority of the population in a novel epidemic, the optimal plan prioritizes activity by susceptibles and has infectious individuals withdraw activity instead, thus maximizing the population's aggregate welfare. Social welfare functions which do not aggregate individual utilities may produce different results; we discuss this briefly in section 5.5.4, but exploring this point is beyond the scope of this analysis.

2.5 Control strategies and trade-offs

Control strategies are typically modeled as approaches to control infection spread subject to economic and political feasibility constraints. A key goal of our approach is to identify the disease-economy trade-offs of different policy approaches induced by individual adaptive responses to infection state and evolution. Importantly, we do not operationalize voluntary and targeted isolation as pre-chosen control strategies. Rather, given individual risk-reward preferences, voluntary and targeted isolation control strategies are emergent adaptive responses under different institutional constraints. Voluntary isolation emerges when susceptible and infectious individuals solve their individual constrained optimization problems, while targeted isolation emerges when susceptible and infectious individuals are able to coordinate on solving their aggregated constrained optimiza-

tion problems. Voluntary isolation behaviors have been observed in other epidemics as well, e.g. H1N1 [18, 19].

Since individual adaptive behavior generates a voluntary isolation strategy, modeling a "no control" approach to epidemic control would require individuals to (unrealistically) not respond to the infection state and its evolution. Formally, this means susceptible individuals behave as though the probability of infection $P_t^I \equiv 0$ in equation 20, i.e. they do not react to the evolution of the epidemic or recognize that their actions can affect their risk exposure. Figure S3 shows how this type of "no control" model compares to voluntary and targeted isolation—the total cases are considerably greater than under voluntary or targeted isolation, while the recession magnitude is only slightly higher than under targeted isolation. As noted in [18, 19, 20], individuals in the real world do in fact exhibit voluntary isolation behaviors, indicating they internalize their ability to alter the P_t^I they experience.

The behavioral responses underpinning voluntary isolation have important implications for control strategy design. For example, empirical results in [20] indicate that the causal impact of lockdown-type policies on activity levels in the United States was considerably smaller than predicted from non-coupled models. Coupled models like ours can provide insight into such findings: in the absence of a lockdown, susceptible individuals would have engaged in voluntary isolation to some degree. Thus, the appropriate counterfactual for control strategy design is not one in which susceptible individuals behave as though $P_t^I \equiv 0$, but one in which they act with awareness that through their actions they can reduce the P_t^I they experience (per equation 20).

This means that blanket lockdowns impose a disease-economy trade-off, and suffer from rebound effects, precisely because of voluntary isolation behaviors and the lack of coordination which generates them. Absent coordination which can support targeted isolation, epidemic control via voluntary isolation or lockdowns imposes a large economic cost because it requires large portions of the population to isolate and limit their economic activities. Targeted isolation offers a way to alleviate the disease-economy trade-off by limiting infection spread while isolating only a small portion of the population.

It is important to recognize that the similarity of infection trajectories under voluntary and targeted isolation does not imply that further case reductions are not *possible* under targeted isolation. Rather, the infection trajectory (and recession trajectory) under targeted isolation emerges as optimal given individual risk-reward preferences. Further case reductions are possible under a targeted isolation policy, but they involve steep recessionary losses for incremental case reductions. For the

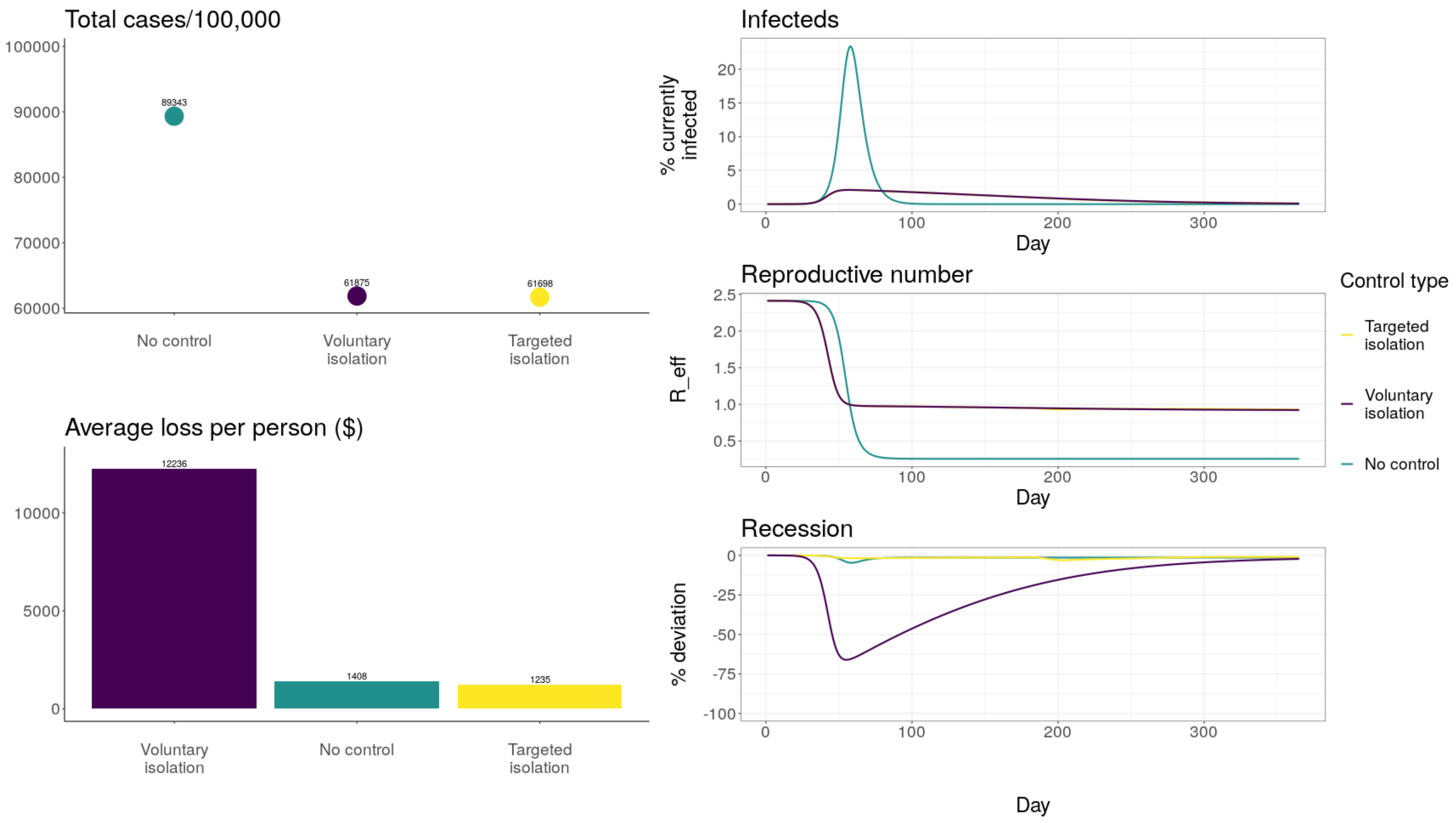

Figure S3: Comparison of model outcomes under voluntary isolation, targeted isolation, and a "no control" approach in which individuals do not react to the evolution of the epidemic or recognize that their actions can affect their risk exposure.

social planner to find it optimal to produce further case reductions, the planner must consider cases to be harmful above and beyond the costs they impose through individual preferences, e.g., due to concerns regarding hospital capacity. This implies that cases impose an additional externality on society which neither susceptible nor infectious individuals internalize—i.e., an additional coordination failure. This can be modelled by incorporating a "case penalty"—a nonnegative function $\Lambda(I_t))$ satisfying $\frac{\partial \Lambda}{\partial I_t} > 0$—into the planner's objective function as shown in equation 28:

$$\max_{\mathbf{l_t},\mathbf{c_t}} \sum_{t=0}^{\infty} \delta^t (S_t u(c_t^S, l_t^S) + I_t u(c_t^I, l_t^I) + R_t u(c_t^R, l_t^R) + D_t \Omega - \Lambda(I_t)) \tag{28}$$

$$\text{s.t., } \forall t, pc_t^S \leq w^S l_t^S \tag{29}$$

$$pc_t^I \leq w^I l_t^I \tag{30}$$

$$pc_t^R \leq w^R l_t^R, \tag{31}$$

subject to the disease dynamics (system of equations 13). Figure S4 shows an example of the potential case reductions under a (purely illustrative) case penalty of $\Lambda(I_t) = 1000(S_t u(c_t^S, l_t^S) + I_t u(c_t^I, l_t^I))I_t$. This specific choice of $\Lambda(I_t)$ implies that an infectious individual at time t costs society roughly 1000 times the utility experienced by susceptible and infectious individuals at time t. That is, a single case costs society roughly the well-being experienced from consumption and leisure by *all* susceptible and infectious individuals for three years, above and beyond the individual costs of infection—roughly \$150,000 in additional total costs to society per case per day, a very large incremental cost. This case penalty produces fewer peak and total cases than the no control or targeted isolation policies, but at a steep economic cost relative to targeted isolation or no control.

While the specific choice and functional form of the case penalty is purely illustrative, it underscores two important points regarding disease-economy trade-offs and control strategies. First, further reductions in cases beyond what targeted isolation produces require greater emphasis to be placed on case control than observed individual risk-reward preferences dictate. Second, while such emphasis can be justified by appealing to additional coordination failures, the optimizations inherent to targeted isolation means additional case control must come with economic costs.

2.6 Individual heterogeneity and coordination failures

For analytical tractability and to highlight the effects of the coordination failure, we study a mean-field SIRD model. However, adding individual heterogeneity, e.g., multiple age classes, does not change the fundamental coordination failure or the use of targeted isolation to address it. We

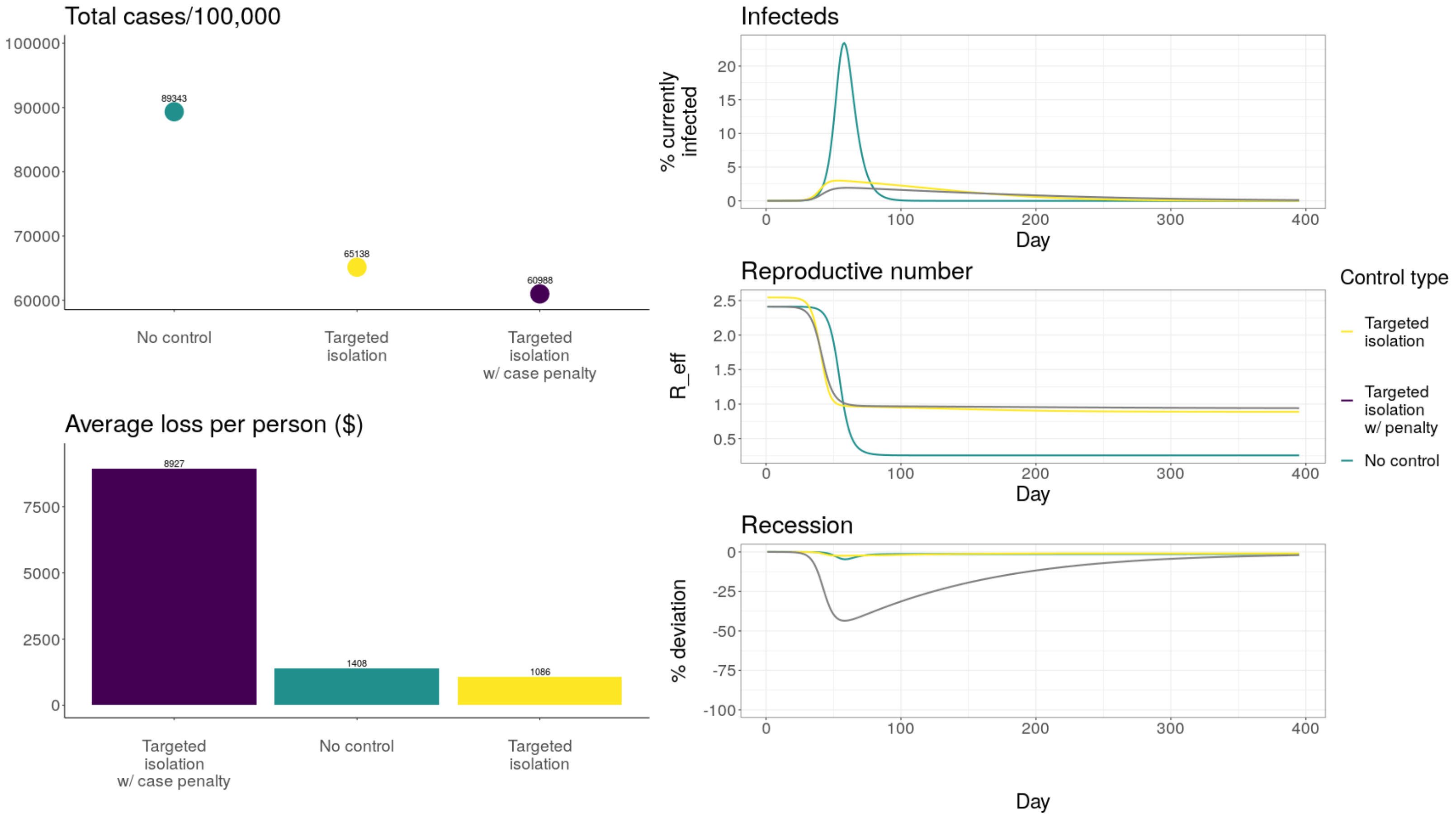

Figure S4: Comparison of model outcomes under a "no control" approach in which individuals do not react to the evolution of the epidemic or recognize that their actions can affect their risk exposure, targeted isolation, and targeted isolation with a "case penalty" which implies the planner finds it optimal to prioritize case reductions above and beyond what individual risk-reward preferences dictate.

illustrate this point below, using a static discrete-choice game theoretic framework for ease of exposition. We focus on Nash equilibria, which are profiles of choices such that no individual can gain by unilaterally changing their choice. We assume throughout that side payments (i.e., transfers between individuals) are possible.

To start, consider a simple stylized game between representative susceptible and infected individuals deciding whether to engage in contactful economic activities (activity level H) or remain home (activity level L). The susceptible individual gains utility from engaging in activities when the infected individual is not present in the common space (so that there is no risk of infection), while the infected individual gains utility from engaging in activities regardless of what the susceptible individual does. The infected individual's sickness prevents them from enjoying the activity as fully as if they were not infected, so letting the susceptible individual's payoff from activity without risk of infection be 1, the infected individual's payoff is $0 < \phi < 1$. ϕ can also be interpreted as productivity loss reflecting the share of asymptomatic individuals as in equation 3. When the individuals do not engage in activity, they get payoffs of 0, and when the susceptible individual risks infection they are worse off than if they had not engaged in activity at all. Letting the susceptible individual's payoff from risking infection to engage in activity be -1 and the populations be of equal size, the game is described by the bimatrix in Table S1.

Table S1: Mean-field two-player SI activity game

		I	
		L	H
S	L	0,0	0,ϕ
	H	1,0	-1,ϕ

Denoting the susceptible individual's activity level by a^S and the infected individual's activity level by a^I, the Nash equilibrium is $(a^S, a^I) = (L, H)$. As long as $\phi < 1$ the Pareto optimum is (H, L). Intuitively, it is always in the infected individual's interest to engage in activity so they always choose H, forcing the susceptible individual to remain home and choose L. However, they would be collectively better off if they could coordinate on having the susceptible individual engage in activity while the infected individual remains home. That is, the Pareto optimum is targeted isolation. This remains true even when we allow for different population sizes and disease dynamics, as long as there are more susceptible individuals than infected individuals (e.g., in the initial phase of a novel epidemic).

Next, expand the game by segmenting the population into two age classes, young (Y) and old

(O). There are four representative individuals, the young susceptible (S^Y), the old susceptible (S^O), the young infected (I^Y), and the old infected (I^O). As before, the infected individuals' payoffs (ϕ^Y, ϕ^O) from engaging in activity are strictly less than the susceptible individuals'. To mimic one feature of COVID-19, suppose $0 < \phi^O < \phi^Y < 1$, so that the old infected individual receives a lower payoff than the young infected individual. The susceptible individuals get a payoff of 1 from engaging in activity as long as no infected individuals are around, and -1 from engaging in activity with any infected individuals around. Letting the row player be S^O and the column player be extended to include the three remaining players (with each combination of strategies being reflected as a unique strategy for the composite column player), the game is as described in Table S2.

Table S2: Age-structured four-player SI activity game

S^Y \ I^Y, S^O, I^O	L, L, L	H, L, L	L, H, L	L, L, H	L, H, H	H, H, L	H, L, H	H, H, H
L	0,0,0,0	0,ϕ^Y,0,0	0,0,1,0	0,0,0,ϕ^O	0,0,-1,ϕ^O	0,ϕ^Y,-1,0	0,ϕ^Y,0,ϕ^O	0,ϕ^Y,-1,ϕ^O
H	1,0,0,0	-1,ϕ^Y,0,0	1,0,1,0	-1,0,0,ϕ^O	-1,0,-1,ϕ^O	-1,ϕ^Y,-1,0	-1,ϕ^Y,0,ϕ^O	-1,ϕ^Y,-1,ϕ^O

Inspection reveals that $(a^{SY}, a^{IY}, a^{SO}, a^{IO}) = (L, H, L, H)$—the susceptible individuals remain home while the infected individuals engage in activity—is a Nash equilibrium. However, the Pareto optimum is $(a^{SY}, a^{IY}, a^{SO}, a^{IO}) = (H, L, H, L)$—the infected individuals remain home while the susceptible individuals engage in activity. The intuition is the same as the two-individual case: infected individuals are better off engaging in activity whether susceptible individuals are present or not, while susceptible individuals are worse off from engaging in activity near infected individuals. Similar results can be derived with n categories of susceptible and infected individuals.

While disease dynamics, varying population sizes, and additional activity choices may lead to different magnitudes of voluntary and targeted isolation for different groups, additional compartmental structure does not change the fundamental nature of the coordination failure. Infected individuals, particularly those who are not suffering from severe symptoms, face incentives to engage in activities. Susceptible individuals have incentives to avoid infected individuals in common spaces. In equilibrium, too many susceptible individuals will remain home, and too many infected individuals will engage in activity. Targeted isolation solves the coordination failure by keeping infected individuals at home while allowing susceptible individuals to engage in activity.

2.7 Parameter values

Tables S3 and S4 list the calibration targets and calibrated parameter values. Table S3 focuses on the targeted equilibrium economic choices, the contact rates, and disease durations. Table S4 focuses on disease and health system parameters.

Table S3: Pre-epidemic equilibrium economic choices, implied utility parameters, and contact rates

Parameter	Description	Source
**Time endowment, pre-epidemic economic choices, & calibration targets**		
$\bar{l} = 12$	Daily hours available for labor and leisure	Consistent with [21] and [22].
$l^* = 0.3333$	% day worked	Consistent with [21] and [22].
$c^* = 58{,}000/365$	Daily consumption $	US 2016 GDP per capita / days in year
$\varepsilon_l = 0.15$	Uncompensated elasticity of labor supply	
**Calibrated utility and budget parameters**		
$\beta = 0.96^{1/365}$	Discount factor (daily)	Implies long-run interest rate of 0.04, as in Goulder et al. (2019).
$\sigma = 1.6417$	Elasticity of substitution between consumption and leisure	Calibration targeting l^* and c^*;
$\alpha = 0.2266$	Utility share of consumption relative to leisure	calculations described in section 2.1
$\eta = 0.1$	Baseline coefficient of relative risk aversion	Described in section 2.1.2.
$\Omega = -672827$	Utility of death	Described in section 2.1.3.
$w = 19.865$	Wage for uninfected individual	Implied by l^* and c^*.
$\phi_I = 0.8555$	Productivity of average infected individual *(as share of non-infected productivity)*	Calculations described in section 2.1.1.
$\phi_S, \phi_R = 1$	Productivity of average uninfected individuals	No disease impact on susceptibles and recovereds.
**Contact parameters**		
$\mathscr{C}(c) = 5.1664$	Average daily contacts at consumption activities	Calculations described in section 2.3.1.
$\mathscr{C}(l) = 7.5131$	Average daily contacts at labor activities	
$\mathscr{C}(o) = 3.5485$	Average daily unavoidable contacts	
$\rho_c = 0.0002$	Consumption contact conversion factor	
$\rho_l = 0.1174$	Labor contact conversion factor	
$\rho_o = 3.5485$	Unavoidable contact conversion factor	

2.8 Empirical validation to observed US economic outcomes during COVID-19

Our model predicts a 66% peak-to-trough decline in GDP under voluntary isolation (shown in Figure 2 of the main text). While our model reports the daily evolution of the economy, most economic data is reported at a quarterly frequency. To compare our results with empirically observed economic data for the US during COVID-19, we aggregate the daily data and present the implied quarterly downturns from our model. We assume the starting level of COVID-19 prevalence in our

Table S4: Disease and health system parameters

Parameter	Description	Source
**Durations**		
$d = 5.1$	Duration of infectiousness (days)	Estimate of mean serial interval from [10]
$d_a = 5.2$	Duration asymptomatic (days)	Estimate of mean incubation period from [10].
$d_h = 19$	Total duration if hospitalized (days)	$d_h = d_a$ + time from symptom onset to hospitalization (3.8 days from Table 2 of Zhang et al. (2020)) + time until hospital discharge (10 days from [23]) = 19 days.
**Probabilities**		
$P^{D*} = 0.015$	Probability of dying conditional on being infected	Estimate of the Infection Fatality Rate provided by the Centre for Evidence-Based Medicine [11].[4] Based on meta-analyses of the best estimates of case numbers and fatalities. Range given of [5.73,5.81]; we take the mean estimate.
$P^{R*} = 0.9423$	Probability of recovering from infection	$1 - P^{R*}$
$P^D = 0.0029$	Probability infected individual dies on a given day	=Prob(leaving I state in given day)AND Prob(dying from infection) = $(1/d)P^{D*}$
$P^R = 0.1931$	Probability of recovering on a given day	=Prob(leaving I state on given day)AND Prob(recovering from infection) = $(1/d)P^{R*}$
$1 - P^R - P^D = 0.8039$	Probability of remaining in infected state on a given day	$1 - P^R - P^D = 1 - 1/d$.
**Other**		
$\varepsilon = 1 * e^{-6}$	Starting population infected (%)	Initial condition. Implies US infection spread began with around 300 people.
$\tau = 0.0314$	Infection per contact	Calculation described in section 2.3.4.
$H = 19.9\%$	% hospitalized	13.8% severe disease + 6.1% critical [24].

model (1 in a million) was roughly the level of COVID-19 prevalence in the US around February 20th, 2020.[5] The comparisons of model-predicted and observed peak-to-trough values are shown in the table below. All actual US economic data is in real terms and taken from the Bureau of Economic Analysis' quarterly "Real Gross Domestic Product and Related Measures" tables.

GDP in our model is closest to "Consumption" in the US economic accounts, since we do not model Investment, Government Spending or Net Exports, which are added to Consumption to form GDP. We also show consumption of services, since we do not explicitly model durable goods purchases. Still, in all cases our model produces quarterly economic outcomes that are broadly consistent with what was observed, despite the simplifying assumptions we make. This suggests our model captures the key features of the epidemic and associated economic downturn.

Table S5: Model versus actual economic outcomes: peak-to-trough (quarterly frequency)

**Actual US economy (BEA)**	
GDP	-35%
Consumption	-38%
Consumption: services	-48%
**Model economic outcomes**	
Voluntary isolation (Quarterly)	-33%

We plot our model's economic outputs at a quarterly frequency alongside observed US economic data in Figure S5. Again, our model provides a good description of the economic reality observed with COVID-19. The economic contraction in our model falls slightly faster than actual data, reflecting the difficulty in knowing precisely when infection prevalence in the real world reached the model's initial condition, as well as the fact that we are comparing with voluntary isolation rather than a blanket lockdown scenario (since blanket lockdowns were sporadically in place in parts of the US).

3 Solving the model

In this section we describe some details of how the model is solved. These include the algorithms used to calculate the equilibrium and optimal plan, the construction of the grid on which the model

[5] Varying the starting date changes the comparison, but does not change the qualitative patterns.

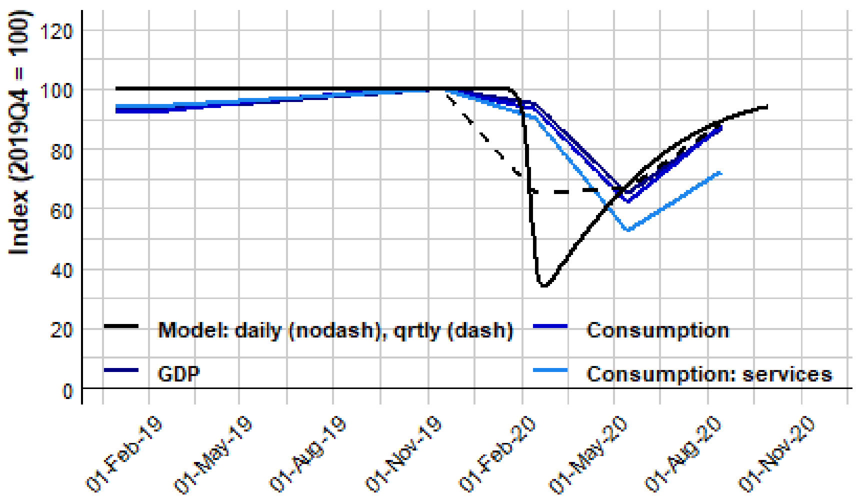

Figure S5: Actual economic outcomes under COVID-19 (blue lines) with model equivalents (black lines). Since our model is at a daily frequency, and actual economic data is not produced at daily frequency, we show daily economic outcomes from our model (straight black line) and quarterly economic outcomes (dashed black line).

is solved, and a correction to the first-order conditions of the susceptible individual's maximization problem to ensure their choices reflect atomistic behavior.

3.1 Solution methods and algorithms

Decisions about how much to consume and work today depend on the current state of the disease and affect the evolution of disease outcomes. We solve the individual's maximization problem using a dynamic programming algorithm.

The algorithm for finding equilibrium can be broken into three steps. First, we construct an initial guess of the terminal value functions (U_0^S, U_0^I, U_0^R) and use the initial state (S_0, I_0, R_0) with the value function guess to determine the optimal c, l for each type. Within the same time period, we iterate over choices to ensure that all individuals are best-responding to each other—we refer to this as the "equilibrium inner loop". Second, these economic variables are used to determine the contact rate between susceptible and infected individuals according to $\mathscr{C}^{SI}(\mathbf{A}) = \sum_i \rho_i A_i^S A_i^I$. Third, the contacts are plugged into the disease dynamics equations (system 13) to find the next-period state, $\{S_1, I_1, R_1\}$. This procedure is repeated until the value function converges. A more formal description is shown in Algorithm 1.

Algorithm 1: Algorithm to calculate equilibrium

1 Set

$$U_0(S,I,R) = guess(S,I,R), \tag{32}$$
$$c_0 = c^*, l_0 = l^*, \tag{33}$$

for each type.

2 Set $i = 1$ and $\delta = 100$ *(some large initial value).*

3 **while** $\delta > \varepsilon$ **do**

4 Set $j = 1$ and $\delta_c = 100$ *(some large initial value)*

5 **while** $\delta_c > \varepsilon_c$ **do**

6 **for** $h \in \{S,I,R\}$ **do**

7 At each grid point in $(grid_S, grid_I, grid_R)$, use a numerical routine to choose (c^h_{ij}, l^h_{ij}) to maximize $U^h_i(S,I,R)$ subject to their budget constraint (as shown in systems 20, 21, 22) and disease dynamics (system 13). When $i = 1$, $U^h_0(S',I',R')$ are used as continuation values; when $i > 1$, $U^h_{i-1}(S',I',R')$ are used. Linear interpolation is used to compute $U^h_{i-1}(S',I',R')$ between grid points.

8 $\delta_c \leftarrow ||\sum_h(l^h_{ij}(S,I,R) - l^h_{ij-1}(S,I,R))||$

9 $j \leftarrow j+1$

10 **end**

11 **end**

12 $\delta \leftarrow ||U_i(S,I,R) - U_{i-1}(S,I,R)||_\infty$.

13 i $\leftarrow$ i+1

14 **end**

The algorithm for finding the optimal plan is simpler, as no equilibrium inner loop is required. It can be broken into similar three steps: First we construct initial value function guesses for each type as in the equilibrium algorithm, then aggregate up to the social welfare function. Using the social welfare function, we then jointly solve for the optimal (social welfare-maximizing) consumption demands and labor supplies for each type. Next we use the choices to determine contacts, and finally use contacts to calculate the next-period state. The procedure is repeated until the social welfare function converges. A more formal description is shown in Algorithm 2.

We set ε to 0.05% of the level of the susceptible type's value function for the decentralized problem (algorithm 1), and to 0.001% of the initial social welfare function for the social planner problem (algorithm 2). The equilibrium inner loop occurs in steps 5-11 of algorithm 1 and typically converges within 1-2 iterations. We use the post-epidemic steady-state lifetime utility for each type as the initial value function guesses for each type in both algorithms.

Algorithm 2: Algorithm to calculate optimal plan

Set

$$U_0(S,I,R) = guess(S,I,R), \tag{34}$$
$$c_0 = c^*, l_0 = l^*, \tag{35}$$

for each type. The social welfare function is then

$$W_0(S,I,R) = S_0 U_0^S + I_0 U_0^I + R_0 U_0^R. \tag{36}$$

Set $i = 1$ and $\delta = 100$ *(some large initial value)*.

while $\delta > \varepsilon$ **do**

  for $h \in \{S,I,R\}$ **do**

    At each grid point in $(grid_S, grid_I, grid_R)$, use a numerical routine to choose (c_i, l_i) to maximize $W_i(S,I,R)$ subject to budget constraints (as shown in system 24) and disease dynamics (system 13). $W_i(S,I,R)$ is given by

$$W_i(S,I,R) = \sum_{h \in \{S,I,R\}} hu(c^h, l^h) + \delta W_{i-1}(S', I', R')$$

    Linear interpolation is used to compute $W_{i-1}(S', I', R')$ between grid points.

  end

  $\delta \leftarrow ||W_i(S,I,R) - W_{i-1}(S,I,R)||_\infty$.

  i ← i+1

end

3.1.1 Grid construction

The appropriate choice of grid points is particularly important in this model, since much of the curvature in the susceptible type's choices occur at low levels of infection prevalence. The grid design balances two considerations: an excessively coarse grid provides low fidelity in the initial stages of the epidemic when $S \approx 1$ and $I,R \approx 0$ (resulting in an epidemic which ends very quickly and a smaller recession), but an excessively fine grid incurs substantial computational overhead due to the curse of dimensionality.

We mitigate this tradeoff by using an expanded Chebyshev grid, which has many attractive properties for economic optimization problems [25]. The key property for our purposes is that it provides greater fidelity near the boundaries of the grid (where it is needed) in exchange for lower fidelity in the interior of the grid (where it is less necessary). The formula for the kth expanded Chebyshev node on an interval $[a,b]$ with n points is

$$x_k = \frac{1}{2}(a+b) + \frac{1}{2}(b-a)\sec\left(\frac{\pi}{2n}\right)\cos\left(\frac{k}{n} - \frac{1}{2n}\right).$$

The only physically-meaningful grid points are within the unit 3-simplex $\{(S,I,R,D) : S+I+R \leq 1, \text{ where } S,I,R,D \geq 0, D \leq 1\}$, or equivalently on the face of the unit 4-simplex, $\{(S,I,R,D) : S+I+R+D = 1, \text{ where } S,I,R,D \geq 0\}$. Typical initial conditions for novel epidemics imply an even smaller subset of points is likely to be visited. For simplicity we solve the model on the entire unit hypercube, $\mathbb{R}^4_{[0,1]}$, though more complex methods can exploit the geometry of the problem for more efficient computation. Methods which avoid off-simplex computations will be increasingly valuable as the model is extended to additional dimensions, e.g. including multiple types within each health status.

3.2 Correctly representing atomistic individual behavior

Susceptible individuals solve the following optimization problem:

$$U^S(S,I,R) = \max_{c,l}\{u(c,l) + \beta[P^I(c,l)U^I + (1-P^I(c,l))U^S(S',I',R')]\} \tag{37}$$

$$\text{s.t.} \quad pc \leq wl \tag{38}$$

$$S' = S(1-P^I(c,l)) \tag{39}$$

$$I' = I(1-P^R-P^D) + P^I(c,l)S \tag{40}$$

$$R' = R + P^R I, \tag{41}$$

where we ignore the dead state for brevity since it does not affect the solution, and

$$P^I(c,l) = \tau(\rho_c c c_I + \rho_l l l_I + \rho_o) I$$

is the probability of getting infected interacting with I-type individuals at consumption, labor, and unavoidable activities.

The representative individual solving the problem above only faces an externality from I types through P^I. There is no externality from the choices of S types since the representative individual controls them all and internalizes this knowledge. This can be seen formally in the first-order condition to the above problem:

$$c^* : 0 = u_c + \beta P_c^I (U^I - U^S) + \beta (1 - P_c^I) \frac{\partial U^S}{\partial c} - \lambda \tag{42}$$

$$\implies u_c = \beta P_c^I (U^S - U^I) + \beta (1 - P_c^I) S P_c^I (U_S^S - U_I^S) + \lambda \tag{43}$$

and

$$l^* : \lambda = w^{-1} \left(-u_l + \beta P_l^I (U^S - U^I) + \beta (1 - P_l^I) S P_l^I (U_S^S - U_I^S) \right) \tag{44}$$

$$\implies (c^*, l^*) : u_c = -\frac{u_l}{w} + \beta (P_c^I + \frac{P_l^I}{w})(U^S - U^I) + \beta S \left(P_c^I (1 - P_c^I) + w^{-1} P_l^I (1 - P_l^I) \right) (U_S^S - U_I^S), \tag{45}$$

where λ is the Lagrange multiplier on the budget constraint and subscripts on P^I and U^S denote partial derivatives. However, the final term in equation 45,

$$\beta S \left(P_c^I (1 - P_c^I) + w^{-1} P_l^I (1 - P_l^I) \right) (U_S^S - U_I^S),$$

contains value function derivatives U_S^S and U_I^S (along with variance terms $P_c^I(1 - P_c^I) + P_l^I(1 - P_l^I)$). These value function derivatives reflect the representative individual's awareness that changes in the population sizes of susceptible and infected individuals affect the marginal value of remaining susceptible. These terms imply susceptible individuals internalize how their choices might affect each other, which is incompatible with atomistic behavior.

To ensure consistency with atomistic behavior, we instead assume the representative susceptible individual maximizes their value *ignoring* the effect of population size changes on the marginal value of remaining susceptible. This yields the following first-order condition instead:

$$(c^*, l^*) : u_c = -\frac{u_l}{w} + \beta(P_c^I + \frac{P_l^I}{w})(U^S - U^I). \quad (46)$$

Equation 46 treats the representative susceptible individual as ignoring how population size changes (which they consider beyond their control) affect the marginal value of remaining susceptible. The above is the activity-structured analog of equation (7) in [26], which does not feature consumption and labor as specific activity classes.

3.3 Model applications

The baseline model is highly stylized, abstracting from many issues likely to complicate the use of targeted isolation strategies. Two particularly relevant issues are information frictions, such as delays in test reporting or low test quality, and non-compliance with policy directives. We explore these issues using solved value and policy functions from algorithms 1 and 2 to generate choices in a given period, subject to the information frictions or partial compliance.

3.3.1 Information frictions: information delays

Health status information is often received with a lag, as (a) individuals may not get tested as soon as they have been exposed to an infectious individual or begin to experience symptoms, and (b) the testing entities may take some time to process and report the results. We implement information delays by having agents react to the lagged state of the environment, abstracting from the sources of the lag. Recent work in [27], [28] has established that typical lags were on the order of 8 days during the early stages of the pandemic and 5 days later on. Based on these estimates, we assume that an exogenous innovation on day 65 of the epidemic improves the lag from 8 days to 5 days.

Information delays accentuate the peak of the epidemic and depth of the recession, and introduce oscillatory dynamics into the evolution of the infection. Intuitively, early on during the epidemic the lags make individuals less responsive to infection spread than they would have been with up-to-date information. This enables the size of the infectious population to grow rapidly. Once individuals begin to observe the height of the peak, their reactions drive a deeper recession than would have occurred with up-to-date information. The ensuing reduction in contacts drives the infection prevalence lower, but individuals don't observe their success immediately. Once they observe the reduction in infection prevalence they begin to increase their activities, but again the lags prevent them from reacting quickly to increasing spread. This dynamic—delayed awareness forcing bigger reactions, which in turn drive large reduction in prevalence, allowing for increased contacts—repeats in a cycle as the epidemic evolves, with the cycles decreasing in amplitude as

susceptible depletion reduces the spread of infection. While these cycles are costly, the gains from reducing the lag from 8 to 5 days appear to be small.

Figure S6 shows how constant information delays of different lengths affect disease and economy outcomes, assuming tests always return the individual's true health state and holding the blanket lockdown design constant. Total cases are relatively insensitive to most delay lengths across all three policies. Economic loss per person is relatively constant across delay lengths under targeted isolation and the blanket lockdown, but declining in delay length under voluntary isolation. Notably, longer information delays create sharper peaks under voluntary and targeted isolation as well as increase the standard deviation of the daily caseload.

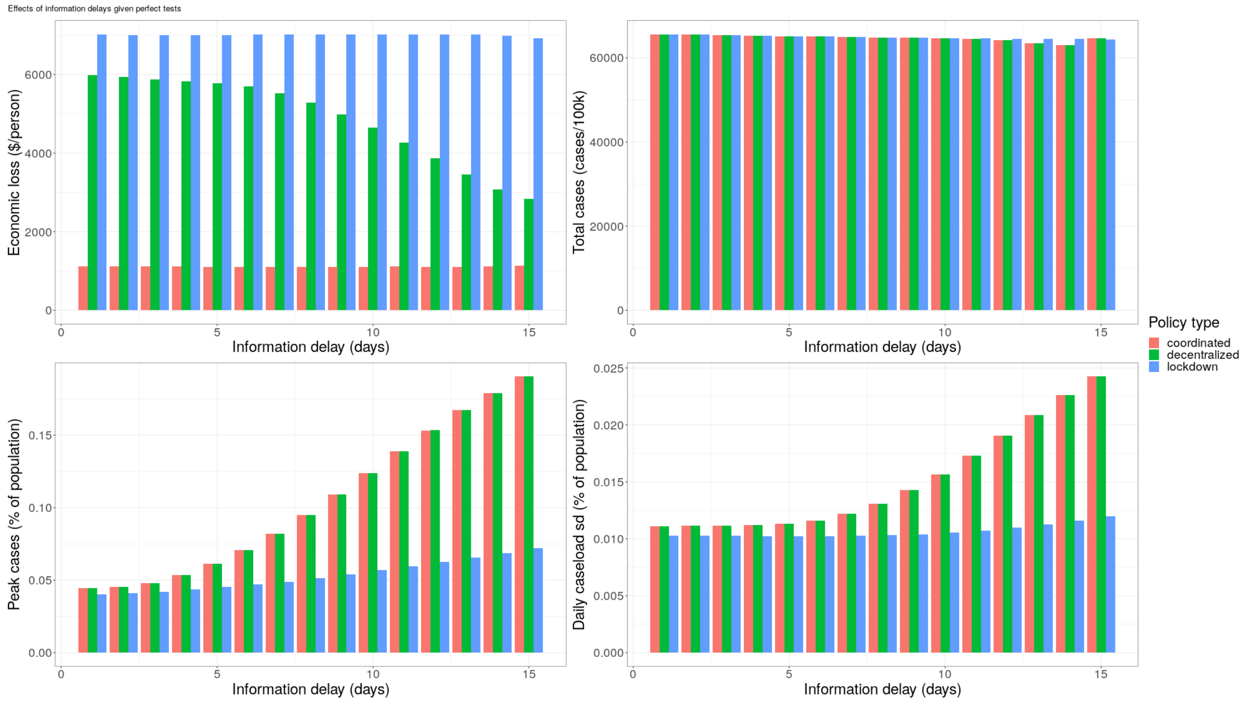

Figure S6: Effects of different information delay lengths on disease and economy outcomes. "Daily caseload sd" is the standard deviation of the fraction of the population infectious at time t.

3.3.2 Information frictions: test quality

Tests may not always report individuals' true health status. Infectious individuals may receive test results claiming they are not infectious, or individuals who are susceptible may receive results claiming they are infectious, and so on. Such errors are an inevitable feature of testing—though they can be reduced significantly, they often cannot be totally removed.

There is substantial heterogeneity in the types of tests available for SARS-CoV-2. The majority of extant SARS-CoV-2 tests are designed to distinguish between two states, e.g., infected or not, recovered or not. The quality of all tests which distinguish between two states can be understood in terms of their *sensitivity* and *specificity*. Sensitivity measures a test's capacity to correctly identify infectious individuals (i.e., the true positive rate) and specificity measures a test's capacity to correctly identify non-infectious individuals (i.e., the true negative rate). As in the case of statistical hypothesis testing, there is often a trade-off between sensitivity and specificity. This trade-off is often measured using the area under the receiver operating characteristic curve, or the AUC. The AUC ranges from 0 to 1, with higher values indicating a better test. An AUC of 1 indicates that the test can perfectly distinguish between types, and an AUC of 0 indicates that the test gets each diagnosis exactly wrong (i.e., 1s are identified as 0s and vice versa).

We abstract from the details of how each test is designed and model a single type of test which identifies an individual's health status.[6] We construct a simple measure of test quality based on the idea of AUC. A test with quality equal to 0 is equally likely to predict any type, regardless of the individuals' true health status. This is equivalent to a test with an AUC of 0.5. A test with quality equal to 1 is perfectly able to identify an individual's true health status, equivalent to an AUC of 1.

We consider two test quality scenarios for each policy strategy: a worst-case where test quality is consistently low (0.1) throughout the epidemic, and a more realistic case where test quality linearly improves over the epidemic and becomes perfect on day 75. In our main specification of test quality effects, we assume individuals take their test result as true and play the optimal action for the type they were assigned. For example, a test with quality 0 is equally likely to tell an infectious individual that they are susceptible, infectious, or recovered. Suppose an infectious individual gets tested and receives a result that they are susceptible. Under the voluntary isolation strategy, they will act as though they are susceptible and withdraw their labor supply in response to rising cases. If instead they were told they were recovered, they would act accordingly. This assumption allows us to construct the aggregate choice of each health type within a given period as a test-quality-weighted convex combination of the zero-quality mixture of actions and the perfect-quality mixture. This is shown for a susceptible type's choice of labor supply in equation 47:

[6]Our notion of testing here encompasses both formal diagnostic testing and individual self-diagnosis based on symptoms and contacts. In the early days of the epidemic the typical symptoms of SARS-CoV-2 infection were not well-understood, leaving many individuals unable to determine if they were infectious even while they were symptomatic. As the signs of infection became better known, individuals became able to infer their own health status despite limited test quality by introspecting on their recent contacts and symptoms. Such self-assessment complements formal testing in producing accurate inference over health status. "Test quality" should therefore be interpreted broadly to include the degree to which individuals can assess their own health status.

$$l_S^q = ql_S + (1-q)\left(\frac{1}{3}l_S + \frac{1}{3}l_I + \frac{1}{3}l_R\right), \tag{47}$$

where l_S, l_I, l_R are the labor supplies of each health type under perfect test quality, q is the current test quality, and l_S^q is the aggregate labor supply for susceptible agents under a test of quality q. We consider robustness of our results regarding test quality to strategic play and feedback between choices in section 3.3.4.

3.3.3 Partial compliance

Not all individuals choose to comply with policy directives, particularly those which they may find costly (like blanket lockdowns or targeted isolation). We examine the effects of limited compliance on the gains from targeted isolation by introducing a "compliant share" parameter. When the compliant share is 1, all individuals behave as the policy (whether a blanket lockdown or targeted isolation) directs. When the compliant share is 0, all individuals behave according to their privately-optimal decentralized behavior (i.e., the solutions to algorithm 1). When the compliant share is c, a $100c\%$ of individuals (of any type) comply with the policy. We therefore write the individuals' choices of activity levels as a compliance-share-weighted convex combination of their choices under the decentralized equilibrium and the policy, shown in equation 48 for a susceptible individual's choice of labor supply:

$$l_S^c = cl_S^p + (1-c)l_S^d, \tag{48}$$

where c is the share of the population which are compliant, l_S^d is their decentralized equilibrium labor supply at the current state, l_S^p is the policy-recommended labor supply at the current state, and l_S^c is the aggregate labor supply for susceptible agents under compliant share c. For simplicity, we assume the compliant share is common to all health types.

3.3.4 Equilibrium responses to limited test quality

We check robustness of our test quality scenario results to different forms of response function (equation 47) using a quantal response equilibrium (QRE). In our main scenario we assume, given a test quality q, that a proportion q of individuals follow the correct action for their health type (whether under a blanket lockdown, targeted isolation, or privately-optimal decentralized behavior) and the remaining $1-q$ randomize with equal weight across the prescribed actions for all three health types. However, agents could instead respond to others' behavior while mixing between actions according to test quality. We examine such equilibrium behavior in this section. We find that under such behavior the benefits of targeted isolation are even more pronounced.

The QRE concept reflects the observation that people's choices get noisier as the intensity of the stimuli become more similar, and has gained traction in experimental and behavioral economics as a model of how choices may display randomness and deviate from Nash equilibrium play [29, 30, 31, 32]. The QRE concept is theoretically consistent with popular random-utility econometric approaches to modeling discrete choices [33, 34], and is equivalent to Bayesian games derived from games of complete information with perturbed payoffs [35].

In a QRE, players choose actions according to a quantal response function (QRF) which specifies their probability of playing each action. Actions with higher payoffs relative to others are more likely to be chosen. A QRF is therefore a mapping from expected payoffs from each action to a mixed strategy over actions. The simplest QRF, called the "Luce ratio", sets the probability of choosing an action equal to the relative magnitude of its payoff compared to payoffs from other actions, i.e., when choosing between three actions A, B, and C we set $Pr(A) = \frac{EU(A)}{EU(A)+EU(B)+EU(C)}$, where $EU(A)$ is the expected utility from playing action A. The logit QRF, shown in equation 49, is more commonly used in economic analysis for its theoretical properties and empirical flexibility:

$$Pr(A) = \frac{e^{\lambda EU(A)}}{e^{\lambda EU(A)} + e^{\lambda EU(B)} + e^{\lambda EU(C)}} \tag{49}$$

The logit QRF includes one tunable parameter, $\lambda \in [0, \infty)$, which represents the strength of the signal the individual receives (alternately, an inverse measure of noise): when $\lambda = 0$ the signal is completely useless (maximal noise) and individuals choose an available action uniformly at random, and as $\lambda \to \infty$ the signal becomes increasingly distinguishable (less noise) and individuals approach their Nash equilibrium choices. Similar to random-utility models of discrete choice, a logit QRF arises when individual errors are drawn IID from a Type 1 Extreme Value distribution.

How should the behaviors embodied in a QRE be interpreted? One interpretation, relevant to our application here, is regarding errors in assessing expected payoffs from each action. Players may make errors in assessing their expected payoff from each action.[7] Their errors in assessing payoffs translate to errors in choosing actions which will maximize their payoffs. In equilibrium, players are aware that they and others will be making mistakes and attempt to mutually "better

[7] Alternately, we can frame these "errors" as best responses by players in an underlying game of incomplete information where players' actual payoffs are private information. The complete information game of the baseline model is then an approximation of the underlying incomplete information game, and the actual payoffs are equal to the modeled lifetime utility up to a privately-known-to-i additive disturbance from a known distribution. See [35] for more discussion on this interpretation. We note that this interpretation is consistent with settings where individuals hold private information regarding their symptoms and contacts and play a Bayesian game with each other to choose actions in response to testing signals.

respond" (rather than best respond as in a Nash equilibrium) to each others' choices. Though logit QRFs imply that a QRE reduces to Nash equilibrium choices when there is no noise, the QRE is not the same as simply playing Nash equilibrium strategies with some noise. In a QRE, there is feedback between the players' errors, with individuals better-responding to each others' mistakes. Unlike a Nash equilibrium, players may fail to maximize their expected payoffs—though they are better responding to others to the extent that their noisy signals permit, with less noise they could choose an even better mixed strategy.

We implement a logit QRE for limited test quality as follows. Within each period, we calculate the optimal noise-free actions for each health type using the policy functions obtained from solving their maximization problems under perfect testing. We then construct their logit QRFs over the possible options using their value functions under the actions available to all health types, subject to noise from limited test quality (λ, described below). Finally, we solve for the fixed point of the agents' logit QRFs and use the resulting probabilities to construct and assign mixed strategies over the available actions for each health type. Each agent receives their own mixing distribution over the actions for all three health types.

We set the noise parameter in each period as $\lambda_t = \frac{1}{1-q_t} - 1$, where q_t is the test quality available on that day. This ensures that $\lim_{q_t \to 0} \lambda_t = 0$ and $\lim_{q_t \to 1} \lambda_t = \infty$. Agents are aware of the optimal choices for each health type, but struggle to determine which action will maximize their own lifetime utility.[8] As information quality improves, agents become better able to assess their payoff from each action and choose the appropriate action for their health type with increasing probability. When tests become perfect, they choose their own health type's action with probability 1. The system of logit QRFs a given period is shown below, with current period subscripts dropped and $'$

[8]We allow agents to use their own type-specific utility functions to avoid having agents play as though they are recovered (the type with the highest lifetime utility) as tests improve.

denoting variables dated $t+1$.

$$
\begin{aligned}
&\forall j, i \in \{S, I, R\} \\
Pr(j \text{ chooses } i|q) \equiv p^j_{i|q} &= \frac{\exp\Big(\lambda(q) v^j(c^i_q, l^i_q)\Big)}{\sum_{i\in\{S,I,R\}} \exp\Big(\lambda(q) v^j(c^i_q, l^i_q)\Big)} \qquad (50)\\
\lambda(q) &= \frac{1}{1-q} - 1 \\
c^j_q &= \sum_{i\in\{S,I,R\}} p^j_{i|q} c^i_q \\
l^j_q &= \sum_{i\in\{S,I,R\}} p^j_{i|q} l^i_q \\
v^S(c^S_q, l^S_q) &= u(c^S_q, l^S_q) + \beta[P^I(c^S_q, l^S_q)U^{I\prime} + (1 - P^I(c^S_q, l^S_q))U^{S\prime}] \\
v^I(c^I_q, l^I_q) &= u(c^I_q, l^I_q) + \beta[(1 - P^R - P^D)U^{I\prime} + P^R U^{R\prime} + P^D \Omega] \\
v^R(c^R_q, l^R_q) &= u(c^R_q, l^R_q) + \beta U^{R\prime} \\
&\text{s.t. } c^j, l^j, U^{j\prime} \text{ solve constrained optimization programs 20-22.}
\end{aligned}
$$

A QRE in a given period is a vector of probabilities, $(p^j_{i|q})_{j,i\in\{S,I,R\}}$, which solve system 50. This equilibrium is a set of mixed strategies for each type, conditional on their own and others' inferential errors, such that no one would choose to alter their strategies even if they learned the strategies of others.

Figure S7 summarizes the effects of QRE play on the effectiveness of blanket lockdowns and targeted isolation. Though the magnitudes of the gains are lower than without QRE play, the benefits of targeted isolation remain substantial. Perhaps notably, individual responses to the epidemic are much sharper across the board, flattening the epidemic curve. The sharpness of individuals' equilibrium responses can be interpreted as an optimal response to noisy signals and uncertainty over one's own health status: faced with noisy signals and attempting to "better respond" to others' choices in the face of noisy signals, many individuals play a precautionary strategy and withdraw their labor supplies. This dramatically increases the depth of the recession relative to the baseline model, though once accurate tests become available individuals begin to play their type-appropriate choices (albeit under different disease dynamics than in the absence of QRE play).

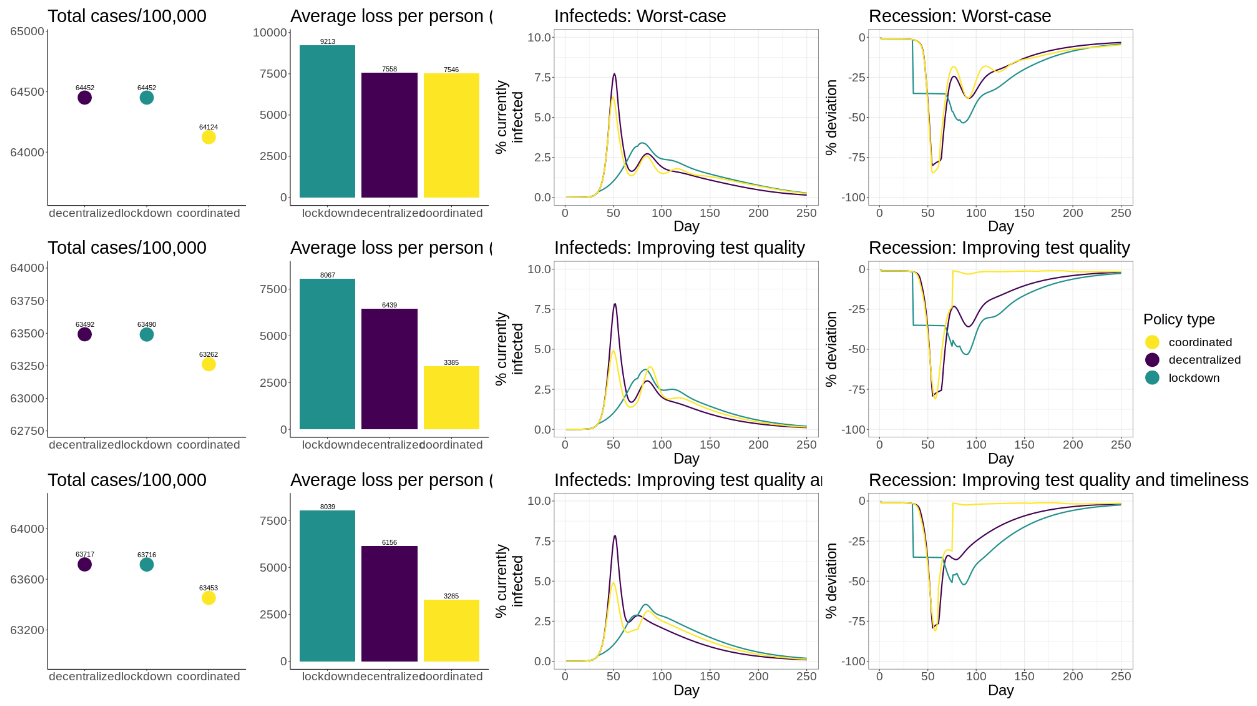

Figure S7: QRE information frictions.

4 Sensitivity to parameter variations

In this section we consider model sensitivity to a host of parameter variations. To assess the maximum possible impact of these variations on the benefits of targeted isolation, we conduct these sensitivity analyses in the baseline model setting without any information frictions and with full compliance.

4.1 Sensitivity to blanket lockdown implementation

As is well known, the design of blanket lockdowns matters to their efficacy, e.g. [36, 37]. To study how lockdown design might affect our conclusions regarding voluntary and targeted isolation, we conduct a sensitivity analysis over 2,500 plausible lockdowns informed by US state-level data on lockdowns.

We define lockdowns as restrictions on businesses or stay-at-home orders, and define the start of a lockdown as the first date such restrictions are put in place. In all US states, restrictions on businesses weakly preceded stay-at-home orders. We classify the lockdown as lifted when the first reopening order is issued.

Since our focus is to explore the space of plausible lockdowns and the observed lockdowns in

US states exhibit relatively little variation, we do not draw from the empirical distribution of lockdown parameters. Instead, we generate "plausible" lockdowns by drawing the duration, timing, and severity as described below.

- Duration of lockdowns: We draw the lockdown duration from a truncated exponential distribution calibrated to match the observed distribution of lockdowns in US states: the lower bound is the minimum observed time until a state reopened, the upper bound is the maximum observed time until a state reopened, and the mean is the average time to reopening.
- Timing of lockdowns: We assume the probability a policymaker implements a lockdown at time t is proportional to the number of cases projected at time t by a standard SIR model with no adaptive economic behavior. This produces a distribution of lockdown implementation dates which is more dispersed than what is observed, though the simulated mean is close to the empirical mean. The empirical mean is calculated as days from the first case recorded in the US.
- Severity of lockdowns: Since the degree to which lockdowns constrain consumption and labor supply is not observed (i.e., the degree to which consumers reduced consumption spending or labor hours supplied due to the lockdown is difficult to disentangle from adaptive responses to changing conditions), we draw *severity* from a uniform distribution over $[0,1]$, with $severity = 1$ representing no lockdown and $severity = 0$ indicating a total lockdown (no consumption or labor activity allowed).

Summary statistics of the data and the simulation parameters are shown in Table S6. Figure S8 shows the results for an ensemble of 2500 plausible lockdown against those of the self and targeted model outcomes. More intense lockdown (where "intensity" is the product of lockdown severity and lockdown duration) produce lower total case rates, but at a high economic cost. Across all simulated lockdown scenarios, blanket lockdowns informed by a mechanistic SIR model can produce fewer cases than decentralized or coordinated behavior, though the total cost in nearly all cases exceeds the cost of the decentralized outcome.

To provide a comparison with a single blanket lockdown with voluntary and targeted isolation policies in the main text, we select the blanket lockdown with the lowest number of cases. This criterion is consistent with the logic that the main goal of blanket lockdown is shift cases forward through time until other control measures become available, reducing the number of cases overall. The case-minimizing blanket lockdown begins at day 91 of the epidemic, lasting for 75 days and applied to 85% of the population. However, we note that the qualitative results hold for all lockdown we draw—blanket lockdowns produce slightly fewer cases than voluntary isolation (and

targeted isolation, for a small subset of the most severe blanket lockdown) but produce a larger economic cost (compared to targeted isolation, a vastly larger economic cost).

Table S6: Summary statistics for lockdown parameters

Variable	N	Mean	St. Dev.	Min	Max
empirical duration	51	48.686	9.501	31	77
simulated duration	2,500	50.396	13.024	31	77
empirical timing	51	57.137	2.967	55	74
simulated timing	2,500	57.292	8.931	23	98
simulated severity	2,500	0.505	0.285	0.001	0.999

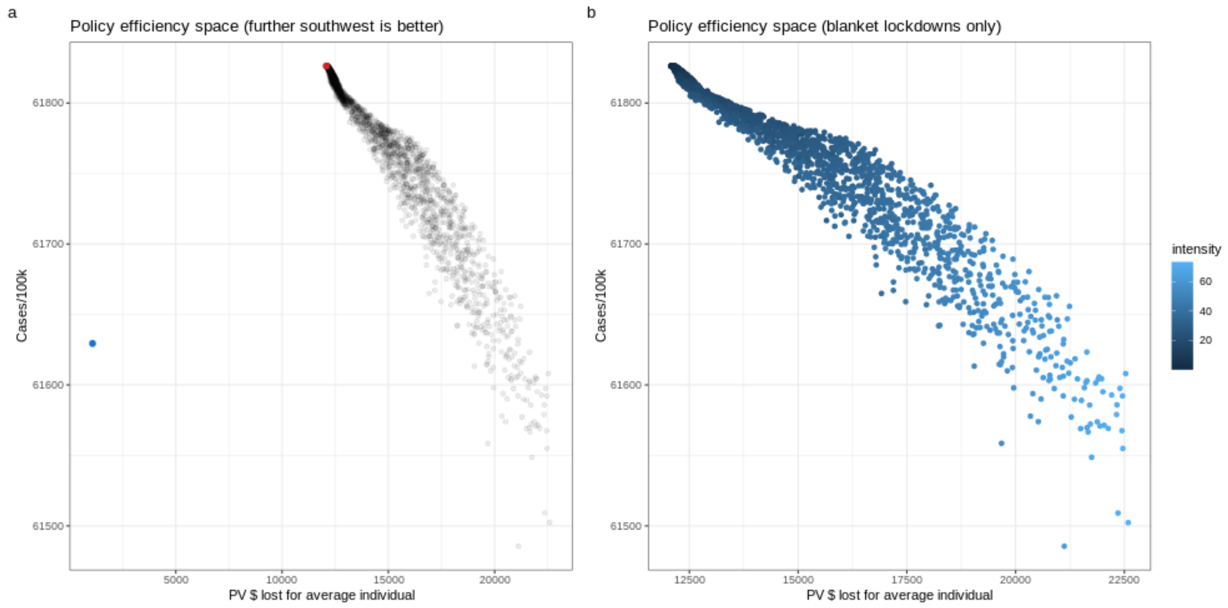

Figure S8: Sensitivity analysis over plausible blanket lockdown scenarios. In panel a, the red dot shows the voluntary isolation outcome while the blue dot shows the targeted isolation outcome. "intensity" in panel b is the product of *severity* and *duration*.

Overall, we find blanket lockdowns tend to induce a disease-economy trade-off. This trade-off is visible in the relationship between disease and economic outcomes traced out by the simulated lockdowns in figure S8. Voluntary isolation is at one end of this trade-off, favoring the economy. Targeted isolation outperforms many blanket lockdowns in terms of both disease and economic outcomes.

4.2 Sensitivity to average case fatality rate (CFR)

In the remaining subsections, we describe tests of the sensitivity of our results on the difference between targeted and decentralized cases to various parameter assumptions. We find that none substantively alter our main conclusions. The results for all of the tests described in this and the following subsections are shown in Figure S9. Note that in the figure all results are presented as percentages of the measure in the targeted isolation case relative to the voluntary isolation case case (e.g. for disease outcomes, a value in the horizontal axis of 0.5 would indicate total cases in the targeted isolation case are half of the voluntary isolation case; for economic outcomes, a value of 0.8 would indicate that the targeted isolation case avoids 80% of the economic cost of the voluntary isolation case).

To study the effects of different age distributions on our findings regarding economic savings, we simulate the model at different case fatality rates covering the range of plausible values from [11]. The lower end of the range of CFRs reflects a society with a mean age near 30, while the upper end reflects a society with mean age over 80. The baseline CFR of 0.015 reflects a society with mean age between 50-59.

In general, we find that higher case fatality rates reduce the economic savings from implementing targeted isolation policies. Intuitively, this is because more severe infections produce greater permanent consequences, inducing both the planner and the decentralized individuals to avert contacts much more drastically. Averting these additional contacts is relatively costlier for the planner, because under targeted isolation the additional isolation must begin to draw from the pool of susceptibles and incur a greater economic cost.

4.3 Sensitivity to income per capita

In this section we test whether our main conclusions are sensitive to GDP/income per capita changes. The income per capita affects the daily income an individual in the model stands to make from working, as well as how much the individual would therefore have available for consumption. In this sense it captures what economic gains an individual can obtain from ignoring the disease and engaging in economic activities. It is therefore central to the overall relationship between the economy and disease.

The figure shows that income per capita does not appreciably affect the ratios – both economy and disease ratios barely move over the realistic range we test. However, income per capita does affect the overall magnitudes for both targeted isolation and voluntary isolation strategies as would be expected given the importance of this parameter for the relationship between the economy and

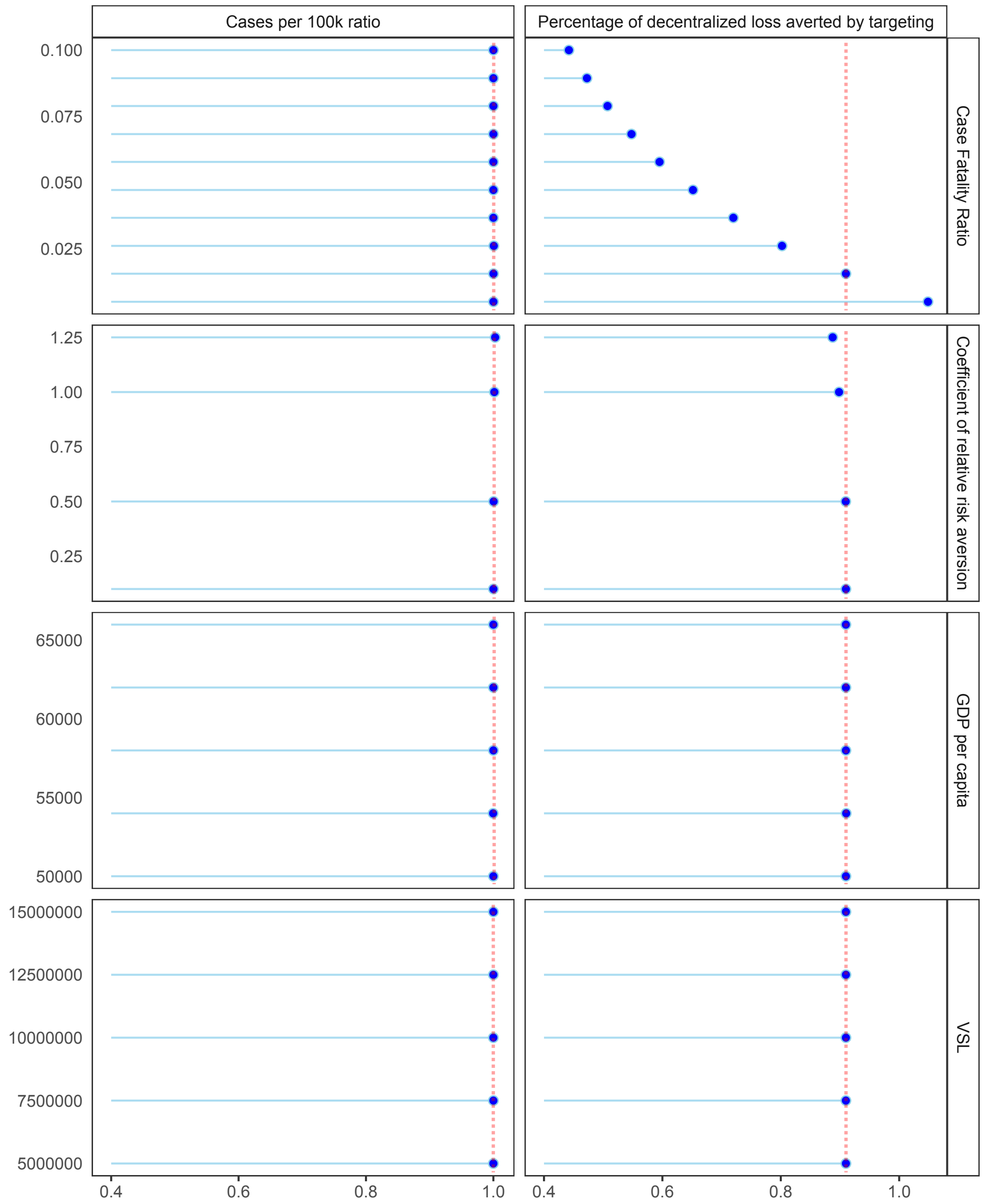

Figure S9: Sensitivity analyses of difference between targeted and voluntary isolation (each 2-box row relates to a separate sensitivity; each sensitivity has the labelled vertical axis). For the horizontal axes, each sensitivity shows (a) left column boxes: "Cases per 100,000" ratio (i.e. "Cases per 100,000" in the targeted isolation case divided by that same measure in the voluntary isolation case); and (b) right column boxes: percentage of decentralized loss averted by targeting (i.e. economic losses in the targeted isolation case divided by economic loss in the voluntary isolation case, then 1 minus this value). **Top row:** Plausible case fatality rates reflecting different population compositions—the dashed line shows the averted loss under the baseline model, where the CFR is taken to be 1.5%.; **Second row:** Sensitivity analysis over coefficient of relative risk aversion, η, adjusting the utility associated with death to hold the VSL constant—the dashed line shows the averted loss under the baseline model, where η is taken to be 0.1; **Third row:** Over GDP per capita influencing daily income and therefore the cost of not working—the dashed line shows the averted loss under baseline GDP per capita of \$ 58,000; **Bottom row:** Sensitivity analysis over the Value of a Statistical Life, adjusting the utility associated with death to hold the CRRA constant—the dashed line shows the averted loss under the baseline model, where VSL is \$ 10m.

disease.

4.4 Sensitivity to VSL

Variation over this range does not appreciably affect the ratios. It does affect the magnitudes of economic losses under both planner and decentralized. We show in the next subsection (section 4.5) that variation in VSL over a much larger range (implied by holding Ω constant while altering η) does induce some change in the ratios, but is still small.

4.5 Sensitivity to risk aversion

To study the sensitivity to different levels of risk aversion, we varied the coefficient of relative risk aversion (η) while holding either the utility associated with death (Ω) or the Value of a Statistical Life constant. Holding either constant while altering η implies adjustment in the other. We consider four values of η: 0.1 (used in the main model simulations), 0.5, 1 (a standard value used in financial contexts), and 1.25 (the upper bound calculated in [1]).

Holding the VSL constant: In this case, the utility associated with death adjusts when solving equation 11 to ensure that the trade-off between consumption and risk of death local to the pre-epidemic equilibrium reflects a VSL of \$10m USD. At higher levels of risk aversion, as the risk of infection rises the planner increases the degree of isolation prescribed for susceptible individuals to reflect their consumption/risk preferences. This results in slightly lower individual recessionary savings under targeted isolation. However, the decentralized individuals do not appreciably change their behavior, since the burden of isolation was already being pushed onto the susceptible individuals. Increases in risk aversion produce no change in the decentralized behavior of recovered individuals (who face no risk) or infected individuals (who face no incentive to isolate in the absence of policy). Figure S9 summarizes these results.

Holding the utility of death constant: When holding the utility associated with death (Ω) constant, more risk-averse individuals require greater compensation to accept the same additional risk. This additional compensation implies a higher VSL. The higher VSL induces the planner to isolate some susceptible individuals as the infection peaks to further reduce deaths, incurring greater economic costs and reducing the average individual recessionary savings under targeted isolation strategies. We show this effect by setting $\Omega = 0$ and varying η over $(0.1, 0.5, 1, 1.25)$.[9] These values of η imply VSLs of around \$2.7m, \$6.2m, \$174.5m, and over \$192b. Note that the total

[9]The utility assocaited with death required to produce a VSL of \$10m when $\eta = 0.1$ produces even higher VSLs when $\eta > 0.1$.

case ratio under targeted isolation relative to voluntary isolation remains unchanged—under voluntary isolation strategies, the additional adjustment is borne by the same number of additional susceptible individuals, producing the same reduction in cases and deaths.

5 Discussion

5.1 Implications for vaccine delivery

While we do not explicitly consider vaccines or vaccine targeting, the planner's solution here provides a starting point for thinking about the rollout of vaccines.[10] Our model implies that as more individuals are vaccinated, in the decentralized equilibrium susceptible individuals will supply more labor and demand more consumption (i.e., engage in more economic activity). To the extent that vaccination reduces the effective reproductive number below 1, this vaccine-induced activity will occur until the effective reproductive number is returned to 1 (following the logic described in [38]). While maintaining $R \approx 1$ will imply similar disease dynamics as in the non-vaccine setting—a relative increase in disease prevalence relative to a non-behavioral model of vaccine delivery—it is possible that vaccination patterns will alter the burden of disease in ways our model cannot describe (e.g., [39]).

The planner would behave similarly to the decentralized equilibrium in allowing more economic activity as vaccination rates increase, but focusing on infected rather than susceptible individuals. That is, since the planner's targeted isolation strategy shields susceptible individual by isolating infected individuals, increasing rates of vaccination would enable the planner to allow asymptomatic infected individuals to supply labor and consume more such that $R \approx 1$ again. As in the voluntary isolation case, it is possible that such actions may worsen disease-related inequities in ways which are beyond our model's scope.

In general, the optimal vaccine rollout plan in models like ours will depend critically on both the nature of the heterogeneity in susceptible individuals as well as the objective function being maximized. For models where the social planner's objective function is an aggregation of individuals' objectives, it is highly likely that the vaccine targeting plan will involve maximizing the economic value generated given a disease progression consistent with $R \approx 1$. Thus, if the heterogeneity involves two types of susceptible individuals, for example those with high contact rates and those with low contact rates, a social planner like ours will likely concentrate vaccination efforts on

[10] Incorporating vaccine targeting explicitly would require additional individual heterogeneity. In particular, it would require at least two types of susceptible individuals with different health or contact parameters.

those with high contact rates, as this will enable additional economic activity per vaccine delivered.

It is important to emphasize that there are multiple dimensions to vaccination targeting. As noted in [39], prioritizing those with high risk versus those with high contacts will induce different mortality/morbidity tradeoffs. These dimensions are somewhat independent of the logic of vaccinating to maximize the economic value of activity, in that they will only affect the targeting plan of a social planner like ours through how individuals value morbidity and mortality. Of course, policymakers may have different objectives. Regardless of the objective being maximized, further research with behavioral heterogeneous-agent models is urgently needed to shed light on these dimensions and potential tradeoffs.

5.2 Implied hospitalization rate

We can calculate the hospitalization rate implied by the infection dynamics of our model. However this is simply an illustration, not the main focus of our analysis and we do not attempt to make detailed statements about hospital burden.

Following [23], we assume the average hospital stay is 10 days long, and calibrate the rates at which individuals recover, die, or remain infected without hospitalization to be consistent with the aggregate dynamics from the baseline model. Formally, this amounts to introducing a new "hospitalized" (H) compartment in the *SIR* model, yielding

$$
\begin{aligned}
S_{t+1} &= S_t - \tau \mathscr{C}^{SI}(\mathbf{A}) S_t I_t, && (51)\\
I_{t+1} &= \bar{P}^I I_t + \tau \mathscr{C}^{SI}(\mathbf{A}) S_t I_t - (P^H + \bar{P}^R + \bar{P}^D) I_t, && (52)\\
H_{t+1} &= P^{HH} H_t + P^{IH} I_t - (P^{HR} + P^{HD}) H_t, && (53)\\
R_{t+1} &= R_t + \bar{P}^R I_t + P^{HR} H_t, && (54)\\
D_{t+1} &= D_t + \bar{P}^D I_t + P^{HD} H_t, && (55)
\end{aligned}
$$

where $P^{IH} = 0.199$, $P^{HH} = 1/10$, $P^{HR} = (1 - P^{HH}) * P^{D*}$, $P^{HD} = (1 - P^{HH}) * (1 - P^{D*})$, and $(\bar{P}^I, \bar{P}^R, \bar{P}^D) = (0.784, 0.017, 0.00025)$ are calibrated to be consistent with the aggregate dynamics. Following [11], we take the baseline case fatality rate (P^{D*}) as 0.015. The percentage of cases hospitalized (P^{IH}) is calculated from a World Health Organization report measuring the percentage of cases that are severe or critical.

Figure S10 shows the results of this illustration exercise. We project that the maximum hospi-

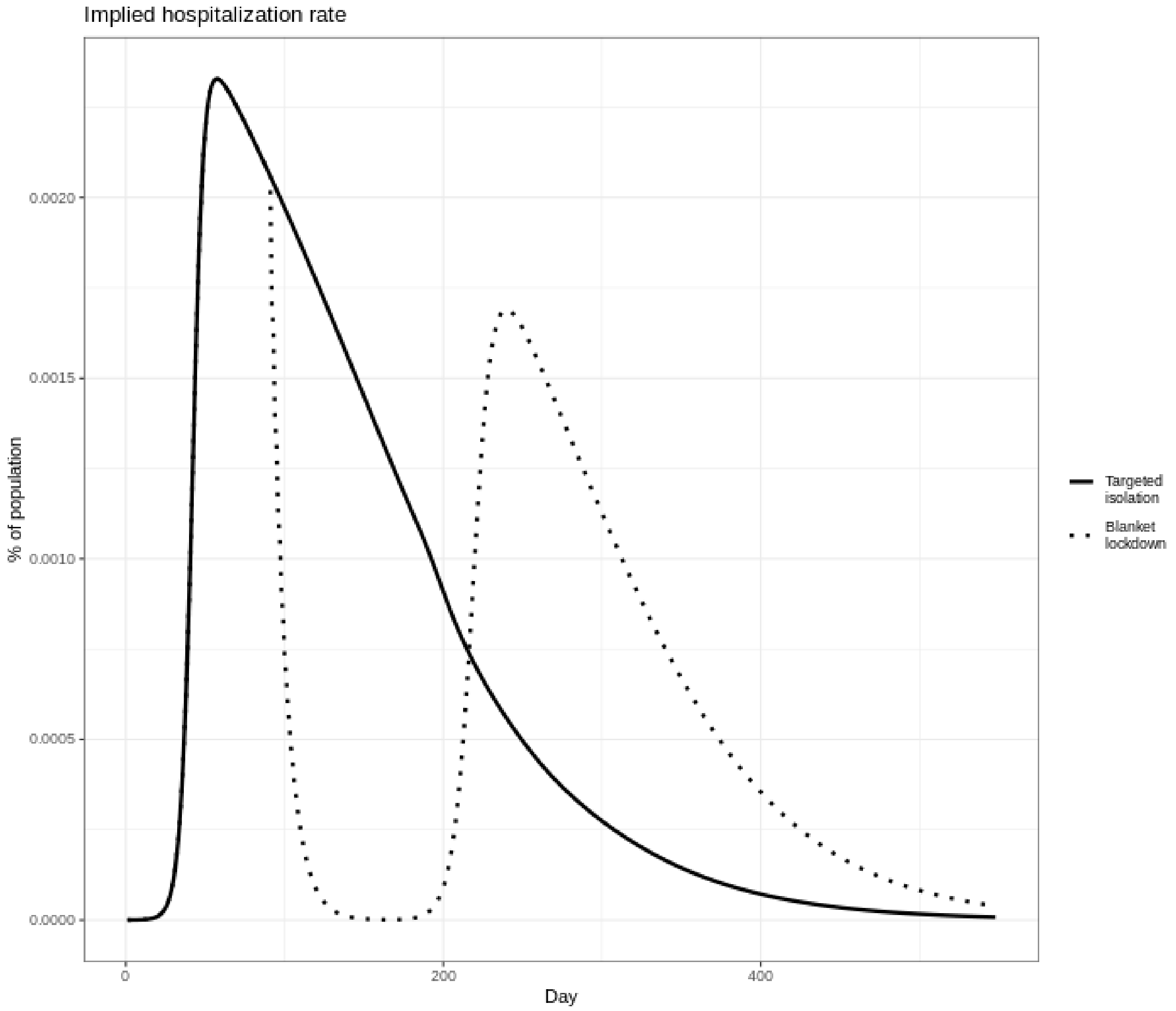

Figure S10: Implied hospitalization rate under targeted isolation and the case-minimizing blanket lockdown shown in Figure 2 of the main text.

tal burden will increase with the share of unavoidable contacts, consistent with evidence regarding household transmission [40, 11]. Similarly, we project that the maximum hospital burden will be highest when consumption contacts are low relative to labor contacts. In such cases, individuals face lower incentives to reduce economic activities and voluntarily isolate, generating higher infection profiles as a result.

The hospitalization rates implied by our model are consistent with estimates from the literature, e.g., [11]. We do not model the costs of approaching or exceeding hospital capacity. Including a convex cost for exceeding the hospital capacity constraint would induce the planner to maintain lower infection rates, with the magnitude of the additional infection avoidance depending on the value of the penalty [41]. What is less clear is how such a penalty would change decentralized behavior. This will depend on the nature of healthcare markets and how effectively crowding costs at hospitals are channeled to individuals, and the degree to which individuals are able to anticipate these crowding costs and act accordingly. It is not obvious what proportion of these costs are or should be internalized by decentralized individuals.

5.3 Implications of blanket lockdowns on hospitalization rates

While our analysis suggests that blanket lockdowns are generally costlier in economic terms than voluntary isolation under universal testing, ignoring the costs of exceeding hospital capacity [11] ignores a major channel for potential savings from blanket lockdowns. If blanket lockdowns are able to shift infections forward to periods when there is lower strain on the healthcare system, then blanket lockdowns may generate nontrivial savings relative to voluntary isolation under universal testing. Figure S11 shows the time paths of GDP deviation and infection prevalence under the ensemble of plausible blanket lockdowns we simulate (see section 4.1), along with the associated marginal distributions of total economic losses and cases per 100,000.

We observe that lockdowns which begin earlier and last longer lead to lower peak infection rates. However, offsetting this potential benefit, relaxing blanket lockdowns tends to cause rebound effects. At the end of a blanket lockdown, susceptible individuals take advantage of the lower risk of infection and engage in contactful activities, causing a spike in cases. Such rebound effects are observed in empirical data from COVID-19 as well as in other environmental settings where policies induce individuals to shift behavior through time (e.g. investment rebounds where policy uncertainty is resolved, pent-up demand, etc.). However, if the lockdown lasts long enough, there will be few enough infected individuals left that the rebound effect will produce a lower peak. These results suggest that blanket lockdowns aimed at preventing hospital capacity from

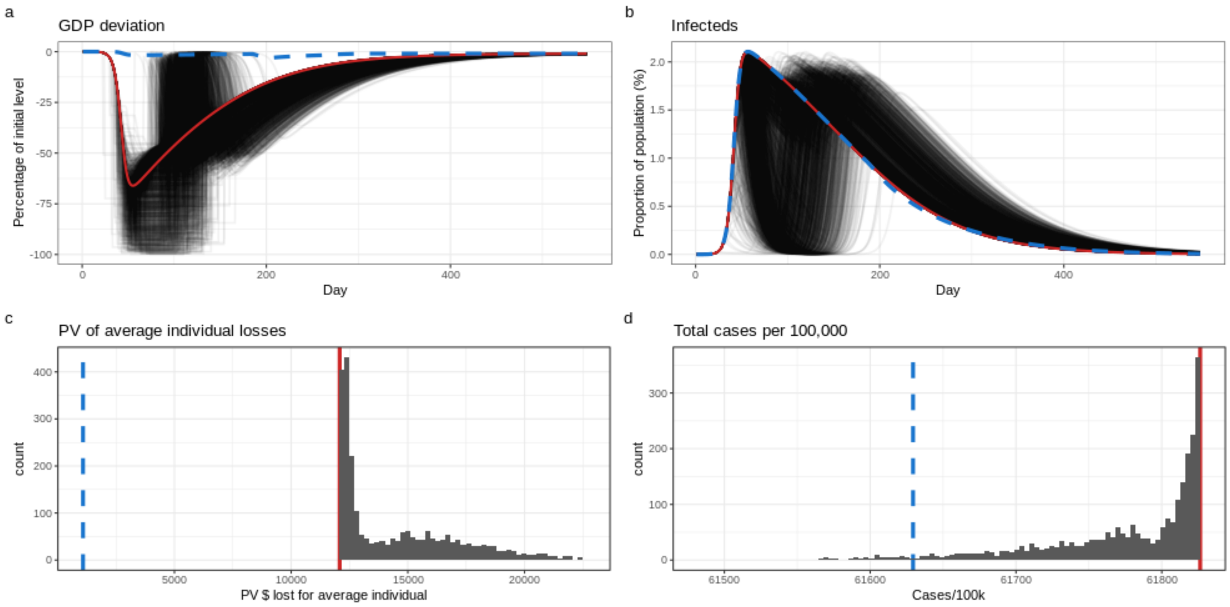

Figure S11: Sensitivity analysis over plausible blanket lockdown scenarios. In panels a and b, the solid red line shows the voluntary isolation path and the dashed blue line shows the targeted isolation path, while the light black lines show alternative blanket lockdown scenarios. In panels c and d, the solid red line shows the voluntary isolation outcome while the dashed blue line shows the targeted isolation outcome.

being overwhelmed must be used with caution. The blanket lockdown must begin early enough to prevent the no-intervention peak in cases, and must last long enough that the rebound effect will not produce a similar peak.

5.4 Possibility of an economy-wide structural change towards more online activity

We test the sensitivity of our main results to the possibility that the pandemic caused a shift to a more online economy, mitigating pandemic infection rates and economic losses. US daily case rates and economic data clearly show that such structural economic changes were unable to completely offset pandemic costs. However, the data and modeling in this section show that this shift to more online activity did at least partly enable some adaptation to the pandemic.

However, we also show that this does not interfere with our main findings about targeted isolation. In principle, an adaptive economic shift may dampen the benefits of targeting to an unknown extent. We show that such a structural change to the economy, of the orders of magnitude observed during the Covid-19 pandemic, still leaves sizeable gains to targeting.

Empirical evidence: We first collect data on the central economic outcomes affected by the

shift to more online activity. The main structural components in our model that represent such an economic structural change are the contact parameters i.e. the contacts associated with a given amount of consumption activity fall because more consumption is conducted online, and the contacts associated with an hour worked fall because more people work from home.

For consumption spending, we observe that the overall shift to online spending was relatively small. Between 2020Q1 (before quarantines and general awareness of the pandemic) and 2020Q2 actual e-commerce retail sales rose from around \$160mn to \$200mn, or from 10% to 16% of overall retail sales. Therefore our change to the consumption contact coefficient is relatively modest.

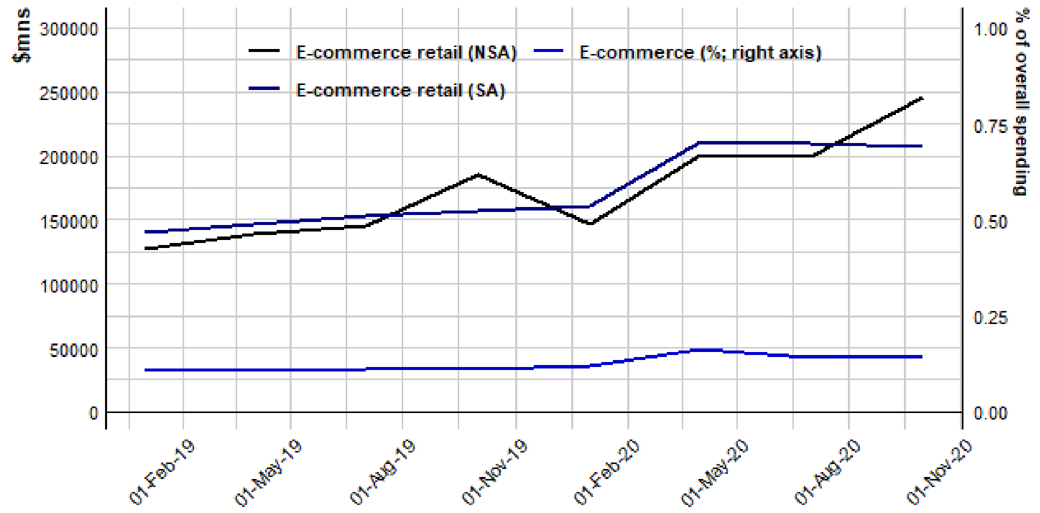

Figure S12: Actual changes to retail sales during the covid-19 pandemic.

By contrast, for working from home hours, we observe that there was a greater shift. Most estimates of the percentage of the population working remotely is based on survey data. The main such survey to have been conducted following the covid-19 pandemic is Bloom (2020) [42], which finds that in the summer of the pandemic 41.9% of workers in the United States were working remotely most of the time. The Bureau of Labor Statistics (BLS) American Time Use survey (job flexibility and work schedules) reports how many respondents reported working remotely in a given week before the pandemic, finding that 24.8% of workers worked from home at that time.

However, comparing the Bloom (2020) estimate to pre-pandemic remote work is challenging because the nature of remote work has changed substantially. Prior to the pandemic remote work was more typically several days a week, whereas during the pandemic remote work became a continuous every day of the week situation. Therefore, here we conservatively assume that the

pre-pandemic level of working from home was 10%, substantially lower than the BLS data, to ensure that we capture all economic changes that occurred due to the pandemic. This however is likely to be too conservative, since Bloom (2020) also notes that working from home likely fell back after its peak in Summer 2020, rather than staying permanent.

Calculating model changes: To loosely convert these changes in data to changes in our model's contact parameters, we apply an extended version of our contact function in equation 15. However, instead of assuming contacts are just a function of overall consumption or overall labor, we assume contacts are a function of online and offline consumption and labor:

$$\mathscr{C}^{SS}(c_{online}, c_{offline}) = \rho_{c,on} c_{S,on} c_{S,on} + \rho_{c,off} c_{S,off} c_{S,off} \tag{56}$$

$$\mathscr{C}^{SS}(l_{online}, l_{offline}) = \rho_{l,on} l_{S,on} l_{S,on} + \rho_{l,off} l_{S,off} l_{S,off} \tag{57}$$

Using the data shown in table S7, we then calculate these coefficients in the pre-pandemic equilibrium (assuming that the online coefficient is zero since online activity does not generate contacts). We then assume these new coefficients stay fixed, while activity levels (consumption and labor, on and offline) change according to the empirical data in the pandemic. This provides a new level of daily contacts pre-pandemic, but with new more-online economy structure. These daily contacts can then be used along with pre-pandemic *overall* consumption and labor to find the coefficients ρ_c, ρ_l as in section 2.3.1. The resulting new coefficients for the new economy with the structural change are shown at the bottom of each panel of table S7.

Results: We use the changes in contact parameters above to simulate the effect of a structural change in the economy from day 40 of the pandemic. This is likely conservative, since in reality it took more than a month for the economy to reach the final stage of this structural change, which we assume holds from this point forward.

The results from this exercise are shown in figure S13. As in our main results, targeting (labelled "plan" in the figure) does lead to a substantial reduction in economic loss (compare the two blue bars in right panel of the bar charts), while not changing disease outcomes (blue bars in left panel of bar charts). This remains true in the "shift" scenario (compare two yellow bars in each bar chart) where economic losses are $5,246 per person in the decentralized case, falling to $925 per person under targeting.

	Pre-pandemic	Post-structural change
**Consumption (online spending)**		
% spending online (from data)	11.0%	↑ 16.5%
Overall consumer spending	158.9	158.9
Spending (online)	17.5	↑ 26.2
Spending (offline)	141.4	↓ 132.7
Contacts (main report)	5.17	
Est contacts (post struct change)		↓ 4.55
ρ_c	0.00020	↓ 0.00018
**Labor (working from home)**		
% working from home (from dat	10.0%	↑ 41.9%
Overall labor hours	8.0	8.0
Hours (wfh)	0.8	↑ 3.4
Hours (office)	7.2	↓ 4.6
Contacts (main report)	7.51	
Est contacts (post struct change)		↓ 3.13
ρ_l	0.117	↓ 0.049

Table S7: Estimates of changes to contact parameters for consumption and labor activities from a structural change in the economy towards more online activity.

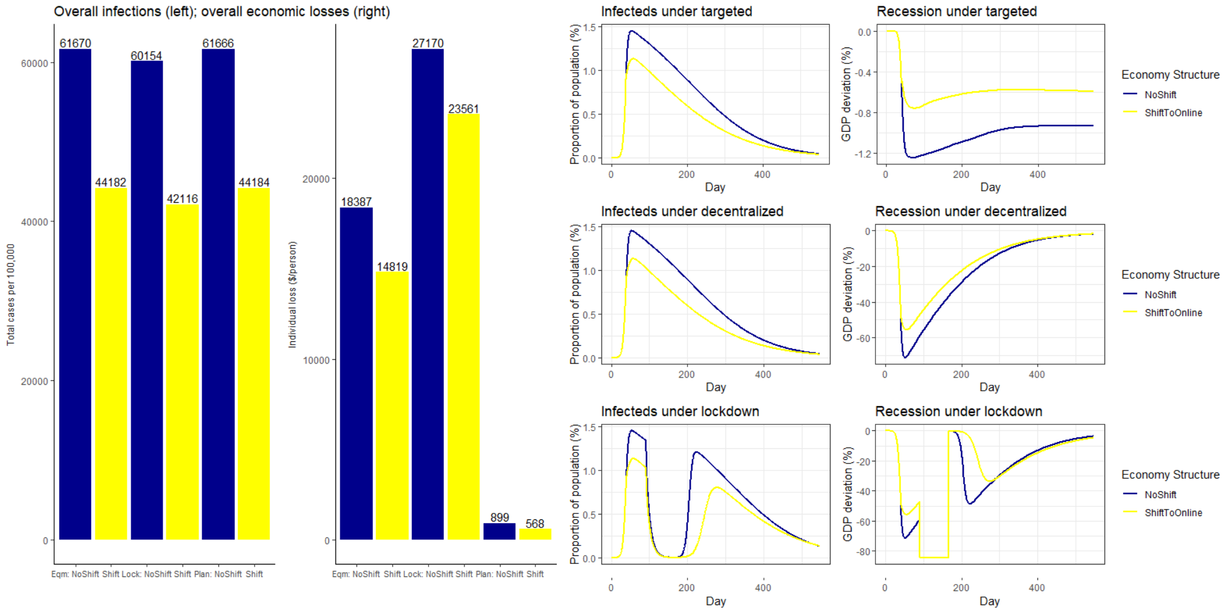

Figure S13: Sensitivity of results to a structural change in the economy towards more online activity. Bar charts left show that although infections and economic losses fall (relative to no structural change) in the case with a shift to more online activity (yellow bars) there is still a sizeable gap between the decentralized (eqm) and coordinated (plan) outcomes.

5.5 Additional considerations

Below we list and briefly describe some additional considerations in developing, interpreting, and extending coupled-systems models such as ours.

5.5.1 Cross-regional comparisons

The importance of model calibration points to the fact that cross-regional (e.g. inter-state or inter-country) comparisons need to be made “ceteris paribus”. That is, the model has a range of parameters that represent a given population. These parameters will be different across different countries and states, meaning that states should be judged against a (potentially counterfactual) state with the same parameterization but a different policy approach. Comparing results of policy outcomes across regions with different parameterizations will result in inferences which mix the effects of the policy approaches with the effects of different parameter values.

5.5.2 Different economic sectors

The possibility of remote work varies by sector. Workers without this option face a choice between whether they go into work and risk contracting the infection or stay at home and lose out financially from being unable to work. Our contact function reflects this dynamic: labor supply is associated with positive contact levels. A smaller labor coefficient in our contact function would imply workers could continue working without many contacts, reflecting the ability to work from home. By contrast a high labor coefficient would imply changing hours worked causes contacts to change a lot, reflecting less ability to work from home. This same approach can be taken to modeling contactless consumption, where low contact coefficients on consumption reflect the ability to consume contactlessly (i.e. changes in consumption demand have a smaller effect on contacts). Figure 4 in the main text shows our main result is qualitatively unchanged when these coefficients are adjusted.

5.5.3 Behavioral adjustment costs

Individuals in the real world often take time to adjust their behavior in response to changing conditions. Whatever the reason, such adjustment costs might shift the dynamics of infection in a small time horizon until individuals’ behavior is adjusted. However, adjustment costs would not qualitatively change the dynamics of the model or the rationale underlying the coordination failure, since individuals would eventually make the adjustments.

5.5.4 Alternative social welfare functions

The targeted isolation strategy is the solution to an optimization problem where individual consumption and labor supply by each health type is chosen to maximize a population-weighted sum of utilities of each health type. Alternative social welfare functions may produce different strategies. For example, a different weighting scheme for type-specific utilities or current cases may alter the degree of targeted isolation as seen in Fig. S4.

We also examined the solution to the unweighted problem (i.e. using a social welfare function with type-specific utilities unweighted by population size, implying the sum of all infected individuals are given equal consideration as the sum of all susceptible individuals) and found that the targeted isolation strategy was still followed, indicating that the welfare losses to the average susceptible individual from reducing activity outweighed the welfare losses to the average infected individual. Alternative social welfare functions, such as a purely-mortality focused objective, may produce substantially different strategies (e.g. a welfare function which considered only total deaths may produce a strategy which suppresses the infection immediately despite a large economic cost) but are beyond our scope here. Fundamentally, any objective function implies some weighting over the interests of individuals with different health types, and any policy involves trading off the interests of these different groups. Population weighting implies that each individual's interests are considered equally regardless of health type.

References

[1] Raj Chetty. Consumption commitments, unemployment durations, and local risk aversion. Technical report, National Bureau of Economic Research, 2004.

[2] Robert McClelland and Shannon Mok. A review of recent research on labor supply elasticities. Technical report, 2012.

[3] Oyungerel Byambasuren, Magnolia Cardona, Katy Bell, Justin Clark, Mary-Louise McLaws, and Paul Glasziou. Estimating the extent of asymptomatic covid-19 and its potential for community transmission: systematic review and meta-analysis. *Official Journal of the Association of Medical Microbiology and Infectious Disease Canada*, 5(4):223–234, 2020.

[4] Jingjing He, Yifei Guo, Richeng Mao, and Jiming Zhang. Proportion of asymptomatic coronavirus disease 2019: A systematic review and meta-analysis. *Journal of medical virology*, 93(2):820–830, 2021.

[5] Matthew Rabin. Risk aversion and expected-utility theory: A calibration theorem. *Econometrica*, 68(5):1281–1292, 2000.

[6] Raj Chetty. A new method of estimating risk aversion. *American Economic Review*, 96(5):1821–1834, 2006.

[7] W Kip Viscusi. Best estimate selection bias in the value of a statistical life. *Journal of Benefit-Cost Analysis*, 9(2):205–246, 2018.

[8] US Environmental Protection Agency. Mortality risk valuation: What value of statistical life does epa use? Technical report, 2020.

[9] Martin S Eichenbaum, Sergio Rebelo, and Mathias Trabandt. The macroeconomics of epidemics. Working Paper 26882, National Bureau of Economic Research, 2020.

[10] Juanjuan Zhang, Maria Litvinova, Wei Wang, Yan Wang, Xiaowei Deng, Xinghui Chen, Mei Li, Wen Zheng, Lan Yi, Xinhua Chen, Qianhui Wu, Yuxia Liang, Xiling Wang, Juan Yang, Kaiyuan Sun, Ira M. Longini, M. Elizabeth Halloran, Peng Wu, Benjamin J. Cowling, Stefano Merler, Cecile Viboud, Alessandro Vespignani, Marco Ajelli, and Hongjie Yu. Evolving epidemiology and transmission dynamics of coronavirus disease 2019 outside Hubei province, China: a descriptive and modelling study. *The Lancet Infectious Diseases*, 20(7):793–802, July 2020.

[11] Robert Verity, Lucy C Okell, Ilaria Dorigatti, Peter Winskill, Charles Whittaker, Natsuko Imai, Gina Cuomo-Dannenburg, Hayley Thompson, Patrick GT Walker, Han Fu, et al. Estimates of the severity of coronavirus disease 2019: a model-based analysis. *The Lancet infectious diseases*, 2020.

[12] The Centre for Evidence-Based Medicine. Global covid-19 case fatality rates. https://www.cebm.net/covid-19/global-covid-19-case-fatality-rates/.

[13] Kiesha Prem, Alex Cook, and Mark Jit. Projecting social contact matrices in 152 countries using contact surveys and demographic data. *PLOS Computational Biology*, 13(9), 2017.

[14] O Diekmann, JAP Heesterbeek, and MG Roberts. The construction of next-generation matrices for compartmental epidemic models. *Journal of the Royal Society, Interface*, 7(47):873–885, 2010.

[15] Robert E Lucas Jr. Econometric policy evaluation: A critique. In *Carnegie-Rochester con-*

ference series on public policy, volume 1, pages 19–46. North-Holland, 1976.

[16] Christopher I Jarvis, Kevin Van Zandvoort, Amy Gimma, Kiesha Prem, Petra Klepac, G James Rubin, and W John Edmunds. Quantifying the impact of physical distance measures on the transmission of covid-19 in the uk. *BMC medicine*, 18:1–10, 2020.

[17] Benjamin M Althouse, Edward A Wenger, Joel C Miller, Samuel V Scarpino, Antoine Allard, Laurent Hébert-Dufresne, and Hao Hu. Stochasticity and heterogeneity in the transmission dynamics of sars-cov-2. *arXiv preprint arXiv:2005.13689*, 2020.

[18] Eli P Fenichel, Nicolai V Kuminoff, and Gerardo Chowell. Skip the trip: Air travelers' behavioral responses to pandemic influenza. *PloS one*, 8(3), 2013.

[19] Jude Bayham, Nicolai V Kuminoff, Quentin Gunn, and Eli P Fenichel. Measured voluntary avoidance behaviour during the 2009 a/h1n1 epidemic. *Proceedings of the Royal Society B: Biological Sciences*, 282(1818):20150814, 2015.

[20] Austan Goolsbee and Chad Syverson. Fear, lockdown, and diversion: Comparing drivers of pandemic economic decline 2020. *Journal of Public Economics*, 193:104311, 2021.

[21] Lawrence H Goulder, Marc AC Hafstead, GyuRim Kim, and Xianling Long. Impacts of a carbon tax across us household income groups: What are the equity-efficiency trade-offs? *Journal of Public Economics*, 175:44–64, 2019.

[22] Bureau of Labor Statistics. American time use, 2019. Data retrieved from BLS website, series TUU10101AA01002867 https://data.bls.gov/timeseries/TUU10101AA01002867.

[23] Dawei Wang, Bo Hu, Chang Hu, Fangfang Zhu, Xing Liu, Jing Zhang, Binbin Wang, Hui Xiang, Zhenshun Cheng, Yong Xiong, Yan Zhao, Yirong Li, Xinghuan Wang, and Zhiyong Peng. Clinical Characteristics of 138 Hospitalized Patients With 2019 Novel Coronavirus–Infected Pneumonia in Wuhan, China. *Journal of the American Medical Association*, 323(11):1061–1069, 03 2020.

[24] World Health Organization. Report of the who–china joint mission on coronavirus disease 2019 (covid-19). Technical report, 2020.

[25] Yongyang Cai and Kenneth L Judd. Stable and efficient computational methods for dynamic programming. *Journal of the European Economic Association*, 8(2-3):626–634, 2010.

[26] Eli P Fenichel, Carlos Castillo-Chavez, M Graziano Ceddia, Gerardo Chowell, Paula A Gonzalez Parra, Graham J Hickling, Garth Holloway, Richard Horan, Benjamin Morin, Charles Perrings, et al. Adaptive human behavior in epidemiological models. *Proceedings of the National Academy of Sciences*, 108(15):6306–6311, 2011.

[27] Sam Abott Sebastian Funk and Stefan Flasche. Reporting delays and temporal variation in transmission in china during the 2019-ncov outbreak. *CMMID Repository*, January 2020.

[28] Qiushi Lin Taojun Hu Shicheng Yu Jing Qin, Chong You and Xiao-Hua Zhou. Estimation of incubation period distribution of covid-19 using disease onset forward time: A novel cross-sectional and forward follow-up study. *Science Advances*, August 2020.

[29] Richard D McKelvey and Thomas R Palfrey. An experimental study of the centipede game. *Econometrica: Journal of the Econometric Society*, pages 803–836, 1992.

[30] Jacob K Goeree, Charles A Holt, and Thomas R Palfrey. Regular quantal response equilibrium. *Experimental Economics*, 8(4):347–367, 2005.

[31] Brian W Rogers, Thomas R Palfrey, and Colin F Camerer. Heterogeneous quantal response equilibrium and cognitive hierarchies. *Journal of Economic Theory*, 144(4):1440–1467, 2009.

[32] Jacob K Goeree, Charles A Holt, and Thomas R Palfrey. Quantal response equilibria. In *Behavioural and Experimental Economics*, pages 234–242. Springer, 2010.

[33] Charles F Manski and Daniel McFadden. Alternative estimators and sample designs for discrete choice analysis. *Structural analysis of discrete data with econometric applications*, 2:2–50, 1981.

[34] Charles F Manski. Daniel mcfadden and the econometric analysis of discrete choice. *The Scandinavian Journal of Economics*, 103(2):217–229, 2001.

[35] Richard D McKelvey and Thomas R Palfrey. A statistical theory of equilibrium in games. *The Japanese Economic Review*, 47(2):186–209, 1996.

[36] Benjamin Roche, Andres Garchitorena, and David Roiz. The impact of lockdown strategies targeting age groups on the burden of covid-19 in france. *Epidemics*, 33:100424, 2020.

[37] Ritabrata Dutta, Susana N Gomes, Dante Kalise, and Lorenzo Pacchiardi. Using mobility data in the design of optimal lockdown strategies for the covid-19 pandemic. *PLoS Computational Biology*, 17(8):e1009236, 2021.

[38] Joshua S Gans. The economic consequences of r = 1: Towards a workable behavioural epidemiological model of pandemics. Working Paper 27632, National Bureau of Economic Research, July 2020.

[39] Shweta Bansal, Babak Pourbohloul, and Lauren Ancel Meyers. A comparative analysis of influenza vaccination programs. *PLoS Med*, 3(10):e387, 2006.

[40] Kiesha Prem, Yang Liu, Timothy W Russell, Adam J Kucharski, Rosalind M Eggo, Nicholas Davies, Stefan Flasche, Samuel Clifford, Carl AB Pearson, James D Munday, et al. The effect of control strategies to reduce social mixing on outcomes of the covid-19 epidemic in wuhan, china: a modelling study. *The Lancet Public Health*, 2020.

[41] Daron Acemoglu, Victor Chernozhukov, Iván Werning, and Michael D Whinston. A multi-risk sir model with optimally targeted lockdown. Working Paper 27102, National Bureau of Economic Research, 2020.

[42] Nicholas Bloom. How working from home works out. *Stanford Institute for Economic Policy Research, policy brief*, June 2020.
