# Supplementary material for: Disease-economy trade-offs under alternative epidemic control strategies
Source: Nat Commun. 2022 Jun 9;13:3319. doi: 10.1038/s41467-022-30642-8 (PMC9178341; doi:10.1038/s41467-022-30642-8)
Supplement: Supplementary file 2 — Reporting Summary [file 41467_2022_30642_MOESM2_ESM.pdf]

nature portfolio

Corresponding author(s): Ana I. Bento and Antonio M. Bento

Last updated by author(s): 2021/08/12

Reporting Summary

Nature Portfolio wishes to improve the reproducibility of the work that we publish. This form provides structure for consistency and transparency in reporting. For further information on Nature Portfolio policies, see our Editorial Policies and the Editorial Policy Checklist.

Statistics

For all statistical analyses, confirm that the following items are present in the figure legend, table legend, main text, or Methods section.

n/a	Confirmed	
☐	☒	The exact sample size (n) for each experimental group/condition, given as a discrete number and unit of measurement
☒	☐	A statement on whether measurements were taken from distinct samples or whether the same sample was measured repeatedly
☒	☐	The statistical test(s) used AND whether they are one- or two-sided<br>*Only common tests should be described solely by name; describe more complex techniques in the Methods section.*
☒	☐	A description of all covariates tested
☒	☐	A description of any assumptions or corrections, such as tests of normality and adjustment for multiple comparisons
☒	☐	A full description of the statistical parameters including central tendency (e.g. means) or other basic estimates (e.g. regression coefficient) AND variation (e.g. standard deviation) or associated estimates of uncertainty (e.g. confidence intervals)
☒	☐	For null hypothesis testing, the test statistic (e.g. F, t, r) with confidence intervals, effect sizes, degrees of freedom and P value noted<br>*Give P values as exact values whenever suitable.*
☒	☐	For Bayesian analysis, information on the choice of priors and Markov chain Monte Carlo settings
☒	☐	For hierarchical and complex designs, identification of the appropriate level for tests and full reporting of outcomes
☒	☐	Estimates of effect sizes (e.g. Cohen's d, Pearson's r), indicating how they were calculated

Our web collection on statistics for biologists contains articles on many of the points above.

Software and code

Policy information about availability of computer code

Data collection	No software was used to collect data. All data are publicly available
Data analysis	R versions v 3.6.0, 3.6.3 and 4.0.3 were used to analyze the generated data. All simulated data were generated using dynamic programming algorithms as described in the SI section 3. Code publicly available on github https://github.com/epi-econ/COVID19_ControlStrategies

For manuscripts utilizing custom algorithms or software that are central to the research but not yet described in published literature, software must be made available to editors and reviewers. We strongly encourage code deposition in a community repository (e.g. GitHub). See the Nature Portfolio guidelines for submitting code & software for further information.

Data

Policy information about availability of data

All manuscripts must include a data availability statement. This statement should provide the following information, where applicable:

- Accession codes, unique identifiers, or web links for publicly available datasets
- A description of any restrictions on data availability
- For clinical datasets or third party data, please ensure that the statement adheres to our policy

The datasets generated during and or analysed during our study are publicly available on github https://github.com/epi-econ/COVID19_ControlStrategies

Field-specific reporting

Please select the one below that is the best fit for your research. If you are not sure, read the appropriate sections before making your selection.

☐ Life sciences ☐ Behavioural & social sciences ☒ Ecological, evolutionary & environmental sciences

For a reference copy of the document with all sections, see nature.com/documents/nr-reporting-summary-flat.pdf

Ecological, evolutionary & environmental sciences study design

All studies must disclose on these points even when the disclosure is negative.

Study description	This study uses a coupled systems mathematical model and calibrated simulations
Research sample	We do not use any research sample
Sampling strategy	We do not conduct any sampling
Data collection	All data used for parameter calibration are taken from publicly available aggregate statistics and both economic and epidemiological parameters are available from published literature. TA, AR and AIB collected data from published epidemiological literature, Bureau of Labor Statistics, and Federal Reserve Bank at St. Louis (FRED) using a spreadsheet. The collected values and sources are listed in SI Tables S3, S4, S5.
Timing and spatial scale	Epidemiological parameters were collected from available literature between January 2020 and January 2021. Contact matrices were used from the literature from 2017 and from 2021. Economic parameters reflect aggregate statistics of the US economy between 2005-2021.
Data exclusions	No data were excluded from the analysis
Reproducibility	All code necessary to reproduce our model and subsequent analysis are publicly available at https://github.com/epi-econ/COVID19_ControlStrategies
Randomization	No randomization was needed for our simulation study design
Blinding	No bliding was needed for our simulation study design

Did the study involve field work? ☐ Yes ☒ No

Reporting for specific materials, systems and methods

We require information from authors about some types of materials, experimental systems and methods used in many studies. Here, indicate whether each material, system or method listed is relevant to your study. If you are not sure if a list item applies to your research, read the appropriate section before selecting a response.

Materials & experimental systems

n/a	Involved in the study	
☒	☐	Antibodies
☒	☐	Eukaryotic cell lines
☒	☐	Palaeontology and archaeology
☒	☐	Animals and other organisms
☒	☐	Human research participants
☒	☐	Clinical data
☒	☐	Dual use research of concern

Methods

n/a	Involved in the study	
☒	☐	ChIP-seq
☒	☐	Flow cytometry
☒	☐	MRI-based neuroimaging
